# AI-Enforced Ultra-Large Virtual Screening Discovers Potent CD28 Binders

**DOI:** 10.1021/acs.jcim.6c00859

**Published:** 2026-06-11

**Authors:** Saurabh Upadhyay, Michele Roggia, Shaoren Yuan, Sandro Cosconati, Moustafa T. Gabr

**Affiliations:** † Department of Radiology, Molecular Imaging Innovations Institute (MI3), 12295 Weill Cornell Medicine, New York, New York 10065, United States; ‡ DiSTABiF, University of Campania Luigi Vanvitelli, 81100 Caserta, Italy

## Abstract

Targeting protein–protein interactions (PPIs) with small molecules is historically challenging due to shallow, solvent-exposed interfaces that lack classical binding pockets. Furthermore, employing traditional structure-based virtual screening (SBVS) across ultralarge chemical spaces to find novel chemotypes imposes prohibitive computational bottlenecks. Here, we report the first prospective, real-world application of the PyRMD2Dock platform, an AI-enforced SBVS workflow that integrates machine learning and standard docking available within the PyRMD Studio suite. To target the structurally demanding immune receptor CD28, a chemically diverse subset of 2.4 million molecules from the Enamine REAL Diversity Space was docked into a cleft adjacent to the canonical ligand interface. These data were used to train 672 classification models, and the optimized model rapidly screened the remaining ∼46 million compounds. Following interaction filtering and clustering, 232 highly prioritized ligands were identified. Experimental validation of 150 purchased candidates yielded a strong hit rate, identifying multiple direct CD28 binders. Lead compounds **100** and **104** exhibited submicromolar affinity (*K*_d_ = 343.8 nM and 407.1 nM, respectively), potent CD28-CD80 disruption, and functional blockade in cellular reporter assays. Furthermore, these compounds successfully reduced cytokine secretion in primary human tumor-PBMC and epithelial tissue coculture models. This study validates PyRMD2Dock as a highly scalable, effective protocol for mining massive chemical libraries to discover small-molecule modulators of challenging immune receptor interfaces.

## Graphical abstract

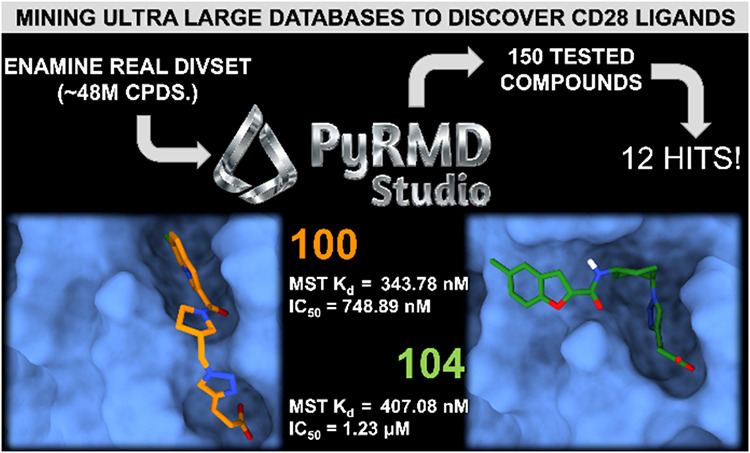

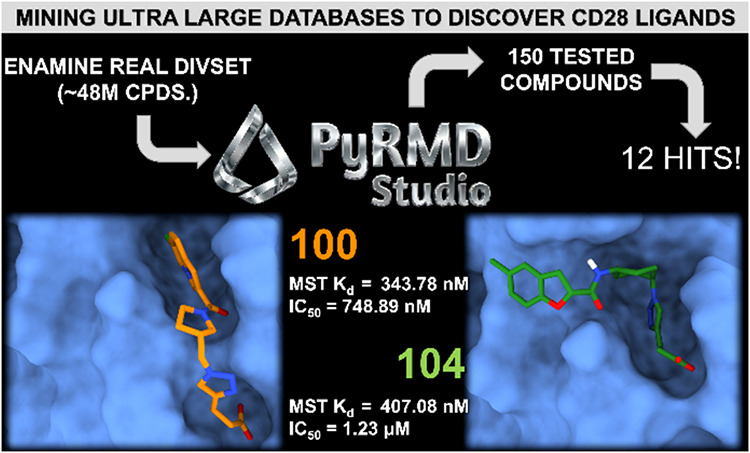

## Introduction

Targeting protein–protein interactions (PPIs) using small molecules remains a formidable challenge in computational drug discovery due to the prevalence of broad, solvent-exposed, and topographically shallow binding interfaces.^1,2^ Unlike enzymes or receptors that typically feature well-defined, deep orthosteric cavities, PPI surfaces frequently lack deep concave pockets suitable for classical structure-based ligand design.^3,4^

The costimulatory immune receptor CD28 serves as a prime example of such a structurally demanding PPI target. CD28 is a homodimeric immunoglobulin-like receptor that engages the ligands CD80 and CD86 through a largely planar and solvent-accessible extracellular β-sandwich surface.^5,6^ The absence of a deeply recessed cavity in this canonical interface makes CD28 an instructive model system for evaluating computational strategies aimed at exploiting shallow surface depressions and partially lipophilic microenvironments.^7^ These architectural constraints have historically limited the development of small-molecule modulators and position CD28 as a structurally informative model for structure-guided targeting of flat immune receptor interfaces.^8,9^

To productively target such intractable interfaces with small molecules, systematic structural interrogation is essential to identify transient or subtle ligandable microenvironments.^10^ Concurrent with these structural analyses, recent computational advances emphasize that screening ultralarge molecular databases significantly increases the probability of discovering novel chemotypes for challenging targets.^11^ However, deploying traditional structure-based virtual screening (SBVS) workflows across billions of compounds imposes prohibitive computational bottlenecks. To overcome these scaling limitations, we utilized PyRMD2Dock within the PyRMD Studio suite.^12^ This comprehensive graphical user interface (GUI) streamlines sophisticated AI workflows, allowing researchers to perform both ligand-based and structure-based virtual screening without requiring advanced coding expertise. PyRMD2Dock bridges the gap between AI-driven ligand-based virtual screening (LBVS) and SBVS by coupling the machine learning (ML) classifier PyRMD with the high-performance docking engine AutoDock-GPU (AD4-GPU).^13,14^ Furthermore, PyRMD Studio incorporates critical code optimizations that increase screening speed by more than 3-fold compared with earlier versions.

By training its predictive machine learning model on the docking scores of a small subset of the chemical library, PyRMD learns to identify chemical features correlated with favorable binding. This methodology enables rapid, effective screening of ultralarge chemical spaces without the immense computational power required to dock every compound explicitly. In this study, we showcase the specific utility of the PyRMD2Dock platform in identifying potent small-molecule disruptors of the structurally challenging CD28–B7 interaction.

To experimentally assess structure-guided ligand prioritization, computationally selected CD28 ligands were evaluated using a tiered validation framework in which complementary biophysical, biochemical, and cellular assays were applied sequentially to assess compound activity at increasing levels of functional complexity. Primary screening was conducted using a TRIC-based assay to identify candidate binders, followed by microscale thermophoresis (MST) to confirm direct molecular engagement and determine binding affinity. Functional consequences of binding were then assessed using competitive CD28–CD80 ELISA and cellular reporter assays, and further validated in primary immune cell coculture systems to establish physiological relevance. This integrated approach enables discrimination between compounds that bind CD28 without functional consequence and those that effectively perturb CD28-mediated signaling, thereby providing a robust framework for functional validation independent of residue-level structural confirmation, while recognizing that such approaches would further refine mechanistic understanding.

Compounds demonstrating reproducible binding and competitive inhibition were further interrogated in CD28-dependent signaling models to establish downstream biological consequences. Reporter-based blockade bioassays quantified suppression of costimulatory signaling, while human immune cell–based systems—including tumor–PBMC and mucosal epithelial–PBMC coculture platforms—enabled evaluation of cytokine modulation under physiologically relevant activation conditions. By linking our AI-enforced virtual screening cascade directly to measurable immunomodulation, these findings not only validate the PyRMD2Dock protocol but also demonstrate that shallow, solvent-exposed immune receptor interfaces can be successfully targeted by small molecules, thus broadening the tractable landscape of complex PPIs.

## Results and Discussion

### AI-Enforced Structure-Based VS against CD28

To discover novel CD28 binders, the PyRMD Studio suite, through its PyRMD2Dock^12^ protocol, was employed as shown in Figure 1.

**Figure 1:**
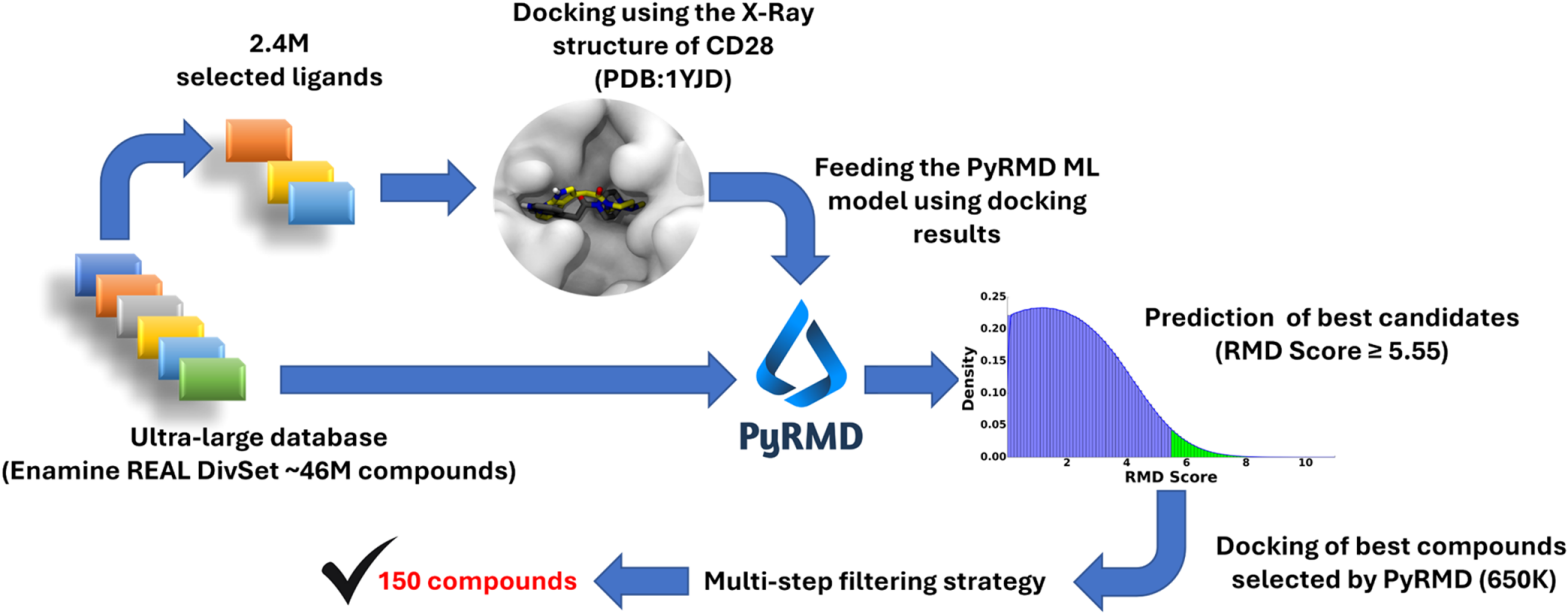
Adopted PyRMD2Dock workflow.

It combines the Ligand-Based VS (LBVS) tool PyRMD^13^ with one of the widely used docking software, AutoDock-GPU (AD4-GPU).^13,14^ By using the PyRMD2Dock pipeline, we established a robust workflow capable of rapid screening across massive chemical libraries to pinpoint molecules with the highest predicted binding affinity to a target protein. The initial phase of this protocol involved the preparation and molecular docking of a subset of 2.4 million compounds for training purposes from the Enamine REAL DivSet database. To ensure a highly representative and chemically diverse data set, these compounds were selected using an RDKit-based diversity picking approach. Specifically, molecular 2D structures were generated from SMILES strings, and Extended Connectivity Fingerprints with a radius of 3 (ECFP6) were computed. A MaxMin algorithm,^15^ relying on Tanimoto distances, was then applied to extract the optimal, maximally diverse subset. This subset was then docked into the X-ray structure of human CD28 in complex with the Fab fragment of a mitogenic antibody (PDB ID: 1YJD).^6^ Here, the primary ligand-binding site of this glycoprotein is defined by the MYPPPY motif (Figure 2A), spanning amino acids 99–104. However, a close inspection of the CD28 3D structure revealed that this canonical binding region is relatively flat and lacks a cavity deep enough to effectively accommodate and stably bind small-molecule drugs. Thus, our structural analysis was redirected toward a distinct pocket adjacent to the natural ligand-binding region. This cavity is delimited by a specific set of amino acids, namely H38, F93, K95, D106, N107, K109, and S110 (Figure 2A,B).

**Figure 2:**
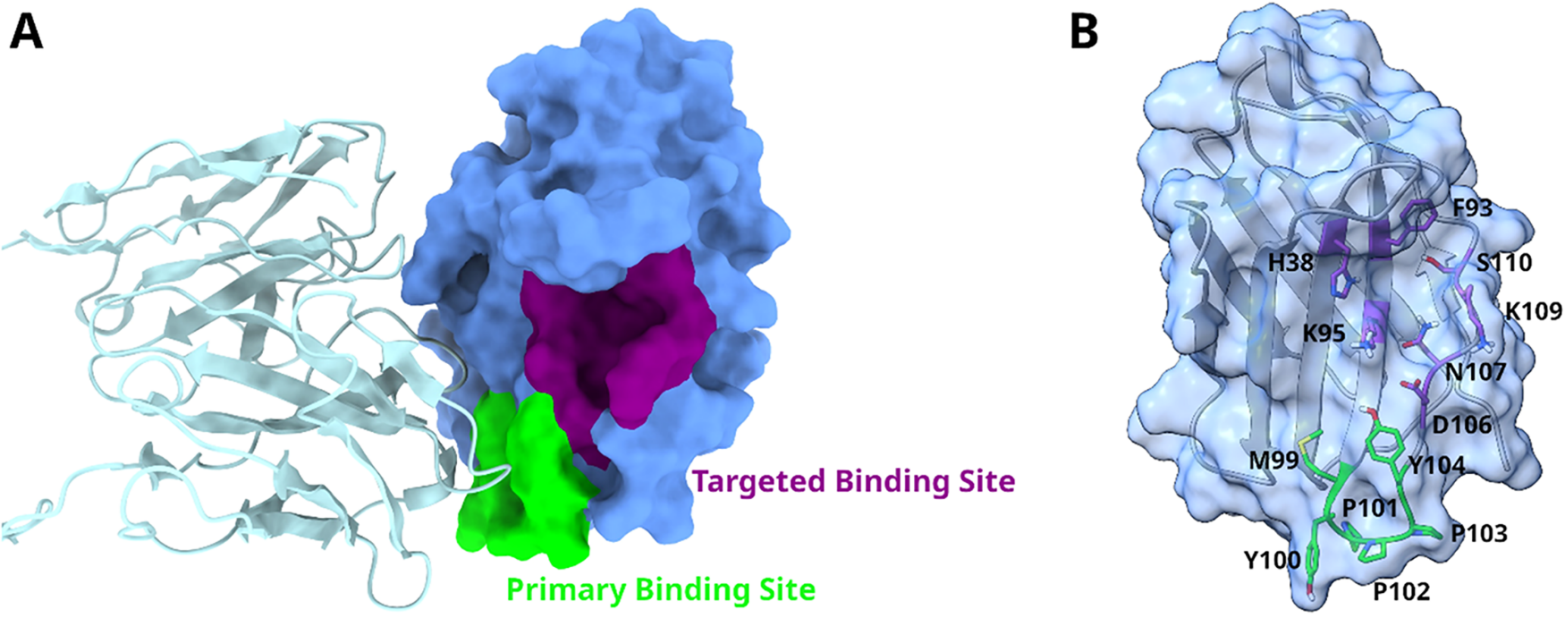
(A) Overall structure of CD28 (blue surface) in complex with the Fab fragment (sky blue ribbons) where the primary binding site (green), and the targeted cleft (violet) are highlighted. (B) A detailed, semitransparent view of the CD28 monomer revealing the specific amino acid residues involved in these regions.

The selection of this targeted binding site is grounded in both crystallographic evidence and structural homology. The existence of this specific cavity is a structural reality documented in the original crystallographic analysis of in complex with the Fab fragment of a mitogenic antibody (PDB ID: 1YJD),^6^ which explicitly highlighted a distinct, CD28-specific pocket adjacent to the canonical ligand-binding region. This cleft emerges from a break in β-strand G and is delineated by key residues, including His38, Phe93, Lys95, Asp106, and Lys109, perfectly aligning with the grid we defined for our virtual screening.^6^ To further evaluate the functional relevance and small-molecule tractability of this pocket, we investigated the features of CTLA-4, a receptor sharing high structural homology with CD28. Specifically, we analyzed the 3D structure of CTLA-4 in complex with the Fab fragment of the monoclonal antibody tremelimumab (PDB ID: 5GGV).^16^ A structural superposition of CD28 onto this complex (Figure 3) revealed that the putative cleft we elected as our target region precisely corresponds to the CTLA-4 area engaged by residues 92–93 and the HCDR3 loop (residues 101–110) of the antibody. Notably, prior structural analyses of the CTLA-4/tremelimumab complex have indicated that the extensive, localized interactions established by the HCDR3 loop of the tremelimumab alone are theoretically sufficient to prevent B7 ligand binding, suggesting that cyclic peptides mimicking this loop could act as functional inhibitors.^16^ This mechanistic insight might imply that massive steric occlusion by a large antibody is not strictly required to achieve a functional blockade. Rather, localized occupancy of this specific microenvironment by smaller structures might be enough to disrupt the broader protein–protein interaction. Based on this crystallographic validation and the homologous functional footprint, the corresponding pocket identified on CD28 was selected as a viable and mechanistically relevant target for our virtual screening campaign.

**Figure 3:**
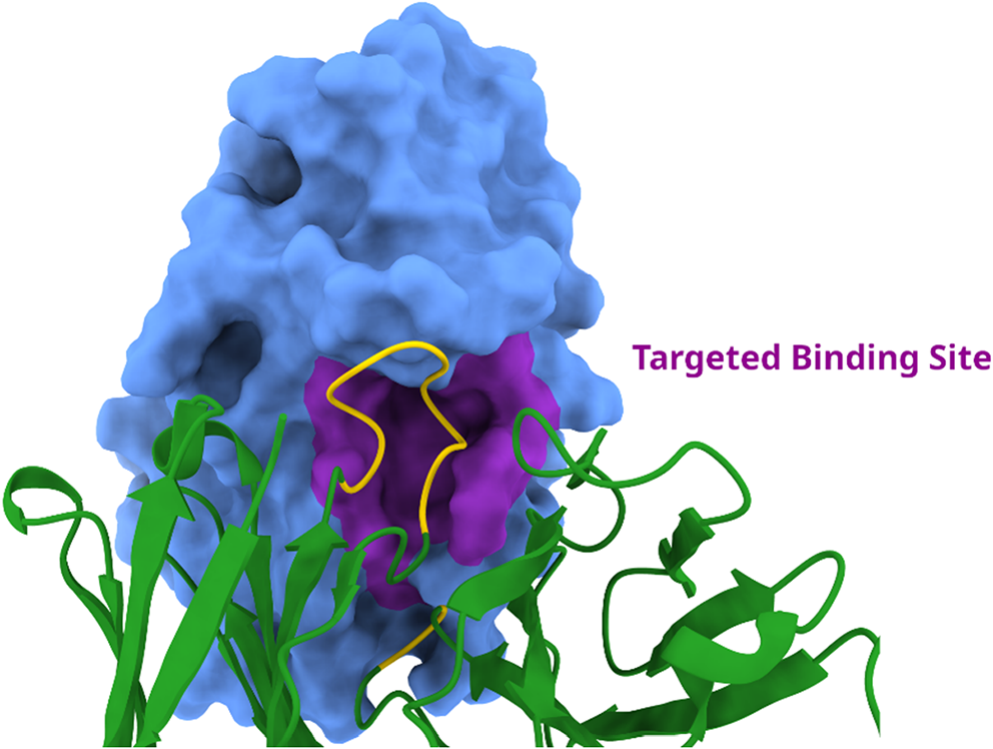
Superposition between CTLA-4 in complex with tremelimumab and the CD28 crystal structure employed for the VS. Tremelimumab is depicted as green ribbons and its HCDR3 loop is highlighted in yellow while the CD28 protein is shown as blue surface.

Following the docking of the training set, for all the compounds the predicted lowest binding free energy (Δ*G*_AD4_) was extracted, and the results were analyzed based on their Δ*G*_AD4_ values distribution. Here, the compounds were separated as “active” and “inactive” depending on their docking score. Specifically, four distinct activity thresholds for the active compounds were established: −8.25, −8.0, −7.75, and −7.50 kcal/mol. Any compound scoring at or below these limits was assigned to the “active” group. Conversely, three thresholds were selected for the inactive compounds: −4.75, −4.50, and −4.25. Molecules with a predicted Δ*G*_AD4_ at or above these cutoffs were classified as “inactive”. By using these parameters, 12 possible data set combinations (4 active thresholds × 3 inactive thresholds) were generated to conduct a thorough benchmarking analysis.

A critical technical consideration in defining these boundaries is the inherent predictive uncertainty of the structure-based methods that we employed (AutoDock), which exhibit an estimated error of approximately ±2 kcal/mol in the prediction of free energies of binding.^17^ To account for this limitation and minimize the impact of docking-related noise on the machine learning model, the classification thresholds were deliberately designed so that the energetic difference between the “active” and “inactive” subsets was always greater than the 2 kcal/mol error margin. By enforcing this wide gap, compounds with ambiguous intermediate scores were intentionally discarded. This strategy aim is to isolate a clearer pharmacophoric signal, enabling the RMD algorithm’s inherent denoising capability to disregard irrelevant chemical features and focus exclusively on the molecular determinants that truly correlate with favorable Δ*G*_AD4_ values.

Additionally, a critical step for the success of the entire workflow is the optimization of the training parameters, particularly the ε cutoff values for both “active” and “inactive” classes. These cutoffs define the maximum allowed projection distance for an unclassified compound within the model’s linear subspace, thus heavily influencing the final classification accuracy. To define these cut-offs, the hyperparameter search was deliberately focused on high ε values, ranging from 0.99 down to 0.70 for the active set, and from 0.95 to 0.60 for the inactive set (with a step of 0.05). This design choice is rooted in the theoretical necessity to grant the model sufficient structural flexibility. Because docking-derived training labels inherently carry computational noise, a high cutoff ensures that the classification function can identify potential binders possessing the core pharmacophoric signal, even if they exhibit significant structural variations compared to the training set. The exploration of highly restrictive, low ε values was intentionally avoided; such values would shrink the active subspace boundary too tightly around the specific training molecules, forcing the model into severe overfitting. Consequently, this would strip the algorithm of its capability to recognize novel chemotypes as potential actives, thereby drastically reducing the True Positive Rate (TPR) and rendering the screening of an ultralarge chemical space ineffective.

To identify the optimal settings, all the possible combinations were systematically evaluated (i.e., 672 different models were evaluated). Here, the employment of the PyRMD Studio suite was instrumental, as it implements a cross-validation adapted version of the StratifiedGroupKFold splitting utility, coupled with Butina clustering. This robust validation strategy ensures that no examples from the same structural cluster are present in both the training and test sets, thereby effectively mitigating data leakage and preventing overly optimistic performance estimates. Thus, we evaluated the performance of all generated models by analyzing a combination of key classification metrics. While maximizing the TPR/FPR trade-off was a primary criterion, it was equally crucial to ensure high Precision and a strong F-score. Therefore, we designed our selection strategy to identify the configuration that offered the best overall balance, prioritizing the model capable of providing an excellent TPR/FPR trade-off alongside robust Precision and F-score metrics. This balance was achieved by the model defined by an “active” threshold of −7.75 kcal/mol, an “inactive” threshold of −4.75 kcal/mol, and ε cutoffs of 0.95 and 0.70 for “actives” and “inactives”, respectively (Supporting Information file “benchmark_results_all.xlsx”). Consequently, this specific setup was selected for the large-scale screening phase.

Using the optimized PyRMD model trained on these docking results, the Enamine REAL Diversity Set (ERDS), a large-scale database comprising 48 million compounds, was screened. The PyRMD2Dock screening module of PyRMD Studio successfully processed this library, ultimately identifying 20,163,675 compounds classified as potential CD28 binders. Notably, refactoring critical internal operations to vectorized equivalents in PyRMD Studio enabled this massive screening to be completed with an approximately 3.3-fold increase in processing speed relative to the previous software version. This screening outcome is the result of two primary factors related to the structural nature of the target and the composition of the chemical space. First, an analysis of the training set (Figure 4A) revealed that the “active” and “inactive” classes, derived from docking scores on the challenging and flat CD28 interface, exhibit a high degree of structural overlap. This intrinsic noise in the training labels creates a blurred decision boundary for any machine-learning classifier. Second, a nearest-neighbor (NN) Tanimoto similarity analysis between the training set and the screened library (Figure 4B) revealed a mean similarity of only 0.38, indicating that the algorithm was challenged with a not related chemical space.

**Figure 4:**
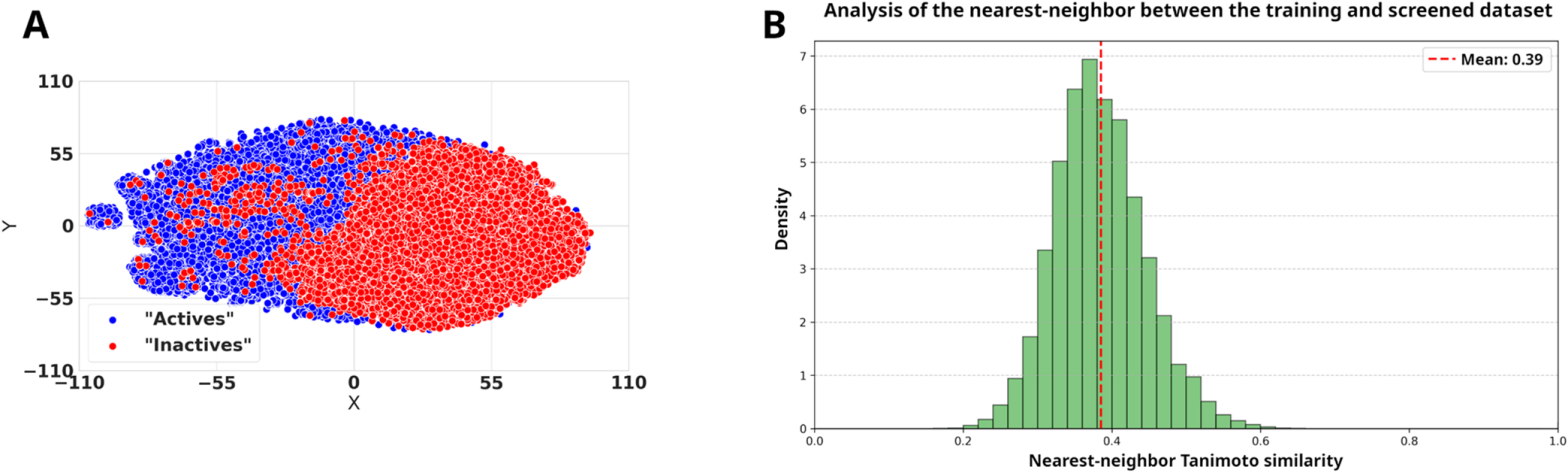
(A) Chemical space of the training set depicted as blue dots (“actives”) and red dots (“inactives”) (B) nearest-neighbor analysis between the training set and the same number of compounds (∼100k) extracted randomly from the Enamine REAL DivSet.

In this scenario, where a classification function trained on overlapping data is applied to not related chemotypes, the binary separation between actives and inactives becomes less distinct. As a direct consequence, the algorithm initially returned a massive number of compounds categorized as “active”. To refine this broad selection, the compounds were ranked based on their calculated RMD score. As a mathematically derived metric, the RMD score does not estimate physical binding affinity; rather, it quantifies the statistical confidence of the prediction by measuring the distance between a compound’s molecular fingerprint and the linear subspaces defined by the “active” and “inactive” eigenvectors. As shown in Figure 5B, compounds yielding low RMD scores exhibit a predicted docking score distribution nearly identical to a random subset. Geometrically, this indicates that their chemical features position them at a similar distance from both training subspaces, resulting in the absence of a clear, resolvable pharmacophoric signal. Conversely, higher RMD scores reflect a closer mathematical proximity to the active subspace, which directly correlates with a significant enrichment of compounds possessing favorable predicted binding energies. The analysis of the top-ranked molecules predicted by PyRMD Studio revealed a clear enrichment in low Δ*G*_AD4_-scores. As expected, an overlap between the distribution curves is present, occurring in proximity to the −7.00 kcal/mol value. This is due to the intrinsic error connected with the docking scoring function, which affects the training process. Interestingly, the mean and the mode of the Δ*G*_AD4_ values obtained when docking the PyRMD-selected ligands (−7.48 and −7.43 kcal/mol, respectively) well approximate the active cutoff Δ*G*_AD4_ value employed in the training process (−7.75 kcal/mol). This outcome is again expected as during the learning training process, the compounds defined as “actives” provided the model with the specific chemical features responsible that in docking calculations contribute to a Δ*G*_AD4_ ≤ −7.75 kcal/mol. Consequently, PyRMD naturally selects new compounds possessing those same structural determinants, resulting in a large population of molecules that achieve similar docking scores while still successfully enriching the overall selection with better-scoring compounds.

**Figure 5:**
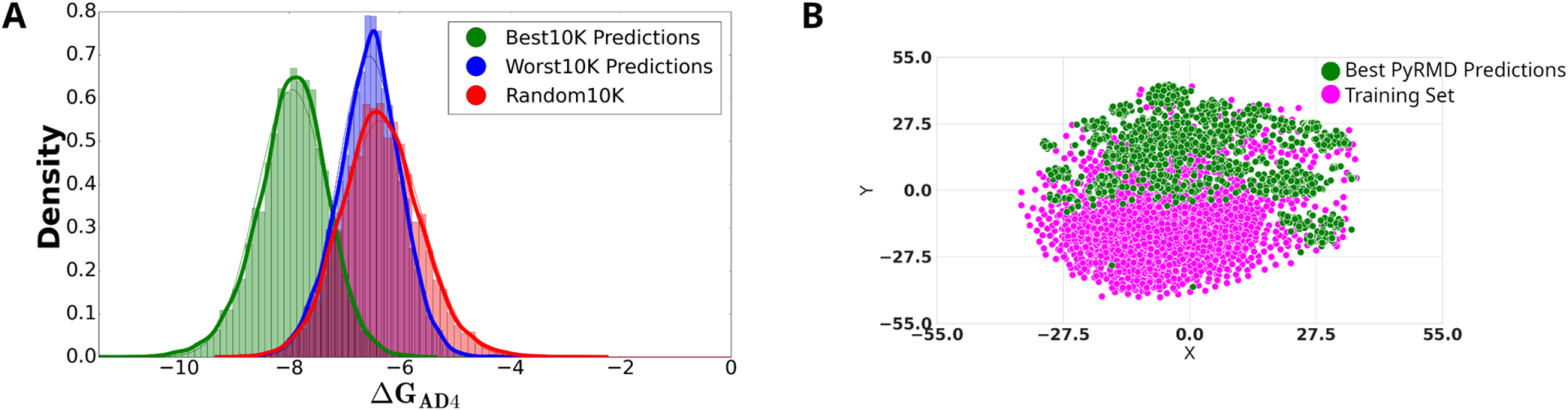
(A) Comparative analysis of the distribution plot of the predicted Δ*G*_AD4_ values between the best 10K compounds predicted by PyRMD Studio (green), the worst predictions (blue) and the randomly chosen subset (red). (B) Chemical space for the PyRMD Studio training and output subsets. The training active compounds are shown in magenta, while the subset consisting of the top screened compounds with the highest RMD scores is shown in green.

Although this analysis provides a clear indication that the RMD can be effectively trained using docking results to predict the outcome of a virtual screening campaign, it does not provide insight into the coverage of chemical space in the output compared to the input. To address this, an analysis was conducted on the chemical space covered by a representative subset of the training set and an equal number of compounds with the highest RMD score output, using t-distributed stochastic neighbor embedding (t-SNE) plots (Figure 5B). The t-SNE analysis highlighted that the training data set derived from docking is intrinsically noisy, as indicated by the lack of chemical clusters of compounds. However, the ML algorithm, by implementing the Marchenko–Pastur distribution, effectively denoises the docking data and facilitates the extraction of meaningful signals. Consequently, PyRMD Studio was able to successfully enrich discrete portions of the training set, identifying families of compounds featuring similar chemical features and disregarding chemical information present in the training set that is identified as noise. However, because the t-SNE algorithm operates by minimizing the Kullback–Leibler divergence between probability distributions of nearest neighbors rather than preserving exact metric distances or global geometry, this projection serves exclusively as a qualitative, descriptive analysis to visually illustrate the data sets chemical space coverage. Therefore, to provide also a quantitative evaluation of this structural enrichment and to quantify the denoising capability of the algorithm in this real-life application, NN Tanimoto similarity analysis was conducted for both training and screening results sets (Figure 6). These results closely match the qualitative clustering observed in the t-SNE projection. The compounds belonging to the training set (Figure 6, red distribution) exhibited a very low mean NN Tanimoto similarity of approximately 0.25. This mathematically confirms that the initial docking-derived data set is highly diverse, scattered, and intrinsically noisy, lacking well-defined structural families. Conversely, the top-scoring compounds prioritized by the PyRMD2Dock protocol (blue distribution) displayed a significantly shifted distribution, reaching a mean NN similarity of approximately 0.55. This substantial increase in structural similarity among nearest neighbors demonstrates that the ML classification did not result in a random sampling of the library. Rather, it successfully filtered out the computational background noise, converging upon and enriching specific, structurally coherent chemical scaffolds.

**Figure 6:**
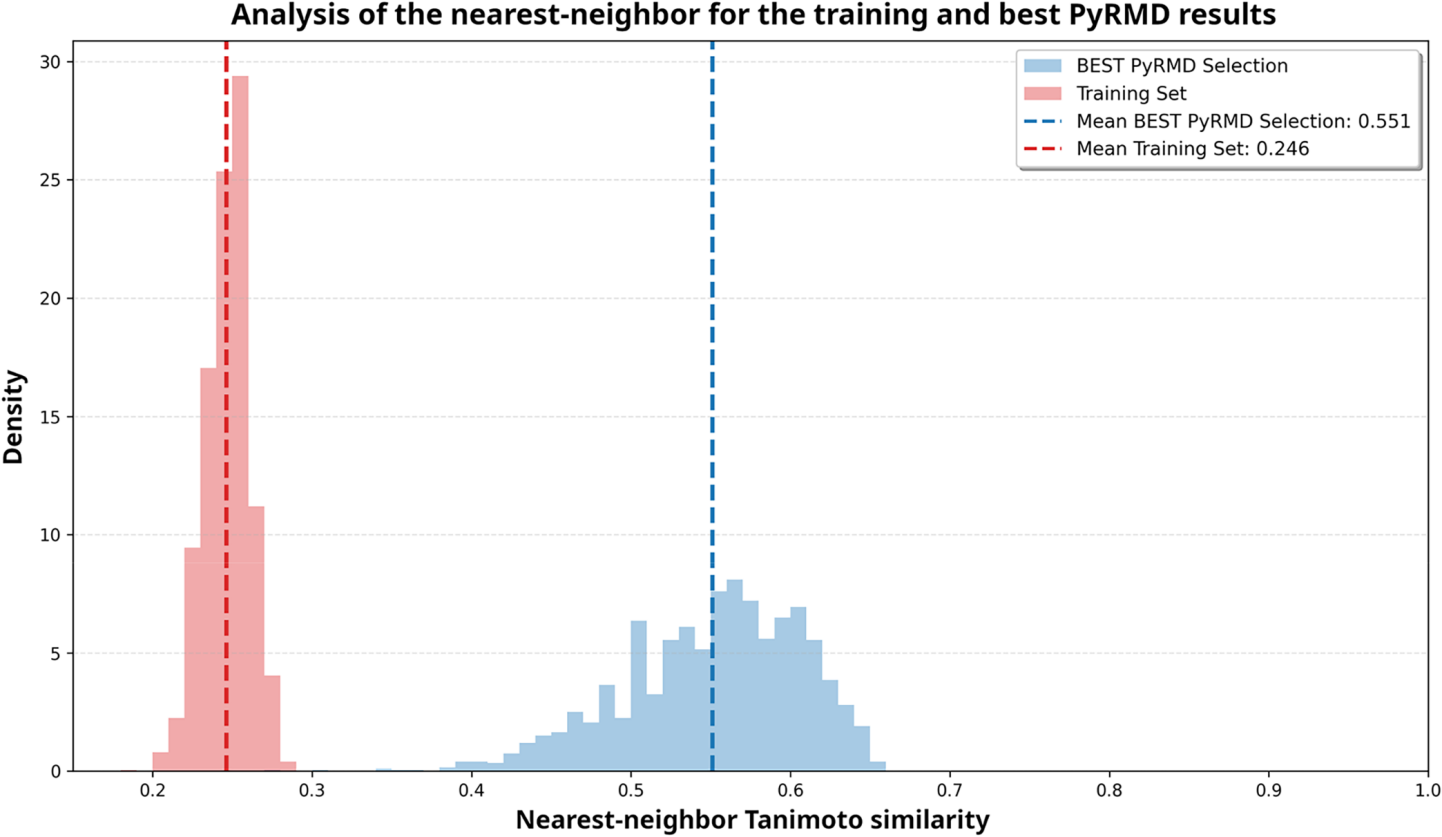
NN analysis of the training set (red distribution) and the top-scroting compounds predicted by PyRMD Studio (blue distribution).

Based on the RMD ranking, we prioritized the top 650,000 candidates for explicit docking. This strategic triage allowed us to focus our computational resources on the most promising 1.35% of the screening library (6.35% of the total database, including training), representing a substantial gain in efficiency compared to an exhaustive docking-only approach.

Subsequently, based on the predicted Δ*G*_AD4_ values, only compounds presenting an energy score ≤−7.75 kcal/mol were advanced to the next stage, reducing the pool to ∼200,000 molecules. To further refine this selection and guarantee that the remaining compounds correctly fit within the identified cleft, a structure-based filter approach was applied. We defined a filtering criterion based on two strategic interaction points spanning the targeted cavity: K109 and C94. The first one was selected as it is one of the key residues delimiting the pocket (Figure 2) and its positively charged side chain should serve as an excellent attachment point, able to establish strong ionic interactions with the ligand candidates. On the other hand, although C94 does not delimit the targeted binding site, it is centrally located at the deep bottom of the pocket.^6^ Thus, filtering out all molecules that failed to establish simultaneous contacts with both residues ensured the selection of ligands strictly occupying the full volume of the targeted cavity. This interaction-based filtering step yielded ∼94,000 ligands, which were subsequently subjected to a solvent-accessible surface area (SASA) analysis using Maestro (Schrödinger) as well as the ΔSASA to quantify the steric fit of the docked compounds. This latter metric represents the ligand’s buried surface area upon binding, calculated as the difference between the SASA of the free ligand and its SASA within the receptor–ligand complex. To narrow down the selection while maintaining chemical diversity, the molecules were clustered based on their structural features. From the various generated clusters, we selected multiple compounds rather than strictly limiting the choice to the single top-scoring molecule per cluster. This selection process was primarily driven by the ΔSASA values, allowing us to come up with a subset of ∼13,000 diverse ligands that exhibited the optimal fit within the pocket. At this stage, visual inspection of the remaining ∼13,000 ligands revealed the presence of three distinct binding orientations within the targeted (Figure 7).

**Figure 7:**
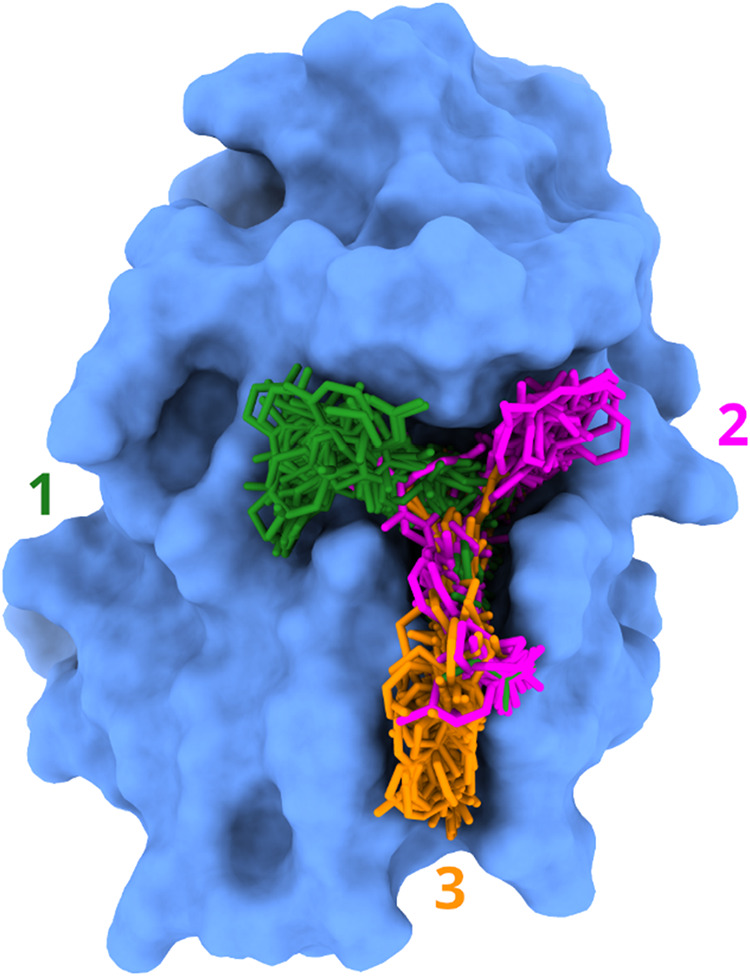
Representative subset of ligands illustrating the three distinct binding orientations identified within the targeted binding site from a pool of ∼13,000 compounds. The compounds belonging to the different binding modes are represented as green, magenta, and orange sticks for clusters 1, 2, and 3, respectively, while the protein is depicted as a blue surface.

Subsequently, to ensure a comprehensive evaluation of these orientations, iterative clustering rounds were performed separately for each of the three groups, categorized by binding mode. Ultimately, this approach led to a final, highly refined selection of 232 ligands. Based on these computational results, 150 compounds were successfully purchased and subjected to biological assays.

### Primary Dianthus Screening of 150 Computationally Selected Compounds

Following large-scale virtual screening of approximately 48 million compounds (described above), a prioritized subset of 150 chemically diverse molecules was selected for experimental evaluation. These compounds were assessed using a Dianthus-based TRIC (temperature-related intensity change) assay employing the purified extracellular domain (ECD) of CD28 to detect ligand-induced perturbations in fluorescence arising from changes in the local environment of the labeled protein under defined biochemical conditions.

Vehicle control wells (2% DMSO in PBST, 0.05% Tween) established a stable baseline signal with a mean Fnorm of 0.863. BPU11, a previously reported CD28-interacting small molecule, was included as a reference control to validate assay responsiveness. This compound reduced the signal to a mean Fnorm of 0.769, confirming that small-molecule engagement of CD28 ECD produces a measurable change within the assay window.^18^

To identify statistically significant signal-altering events, a stringent ±5 standard deviation threshold relative to the vehicle mean was applied, corresponding to an Fnorm interval of 0.828–0.894. The majority of screened compounds generated Fnorm values within this confidence range, indicating no detectable interaction under the assay conditions.

In contrast, 12 compounds reproducibly produced Fnorm values outside the predefined statistical interval. These molecules were therefore classified as primary hits, corresponding to approximately 7.3% of the tested subset (Figure 8A). Given that the assay contains only purified CD28 ECD and small molecules, these signal deviations reflect compound–protein interactions sufficient to alter the Dianthus readout.

**Figure 8:**
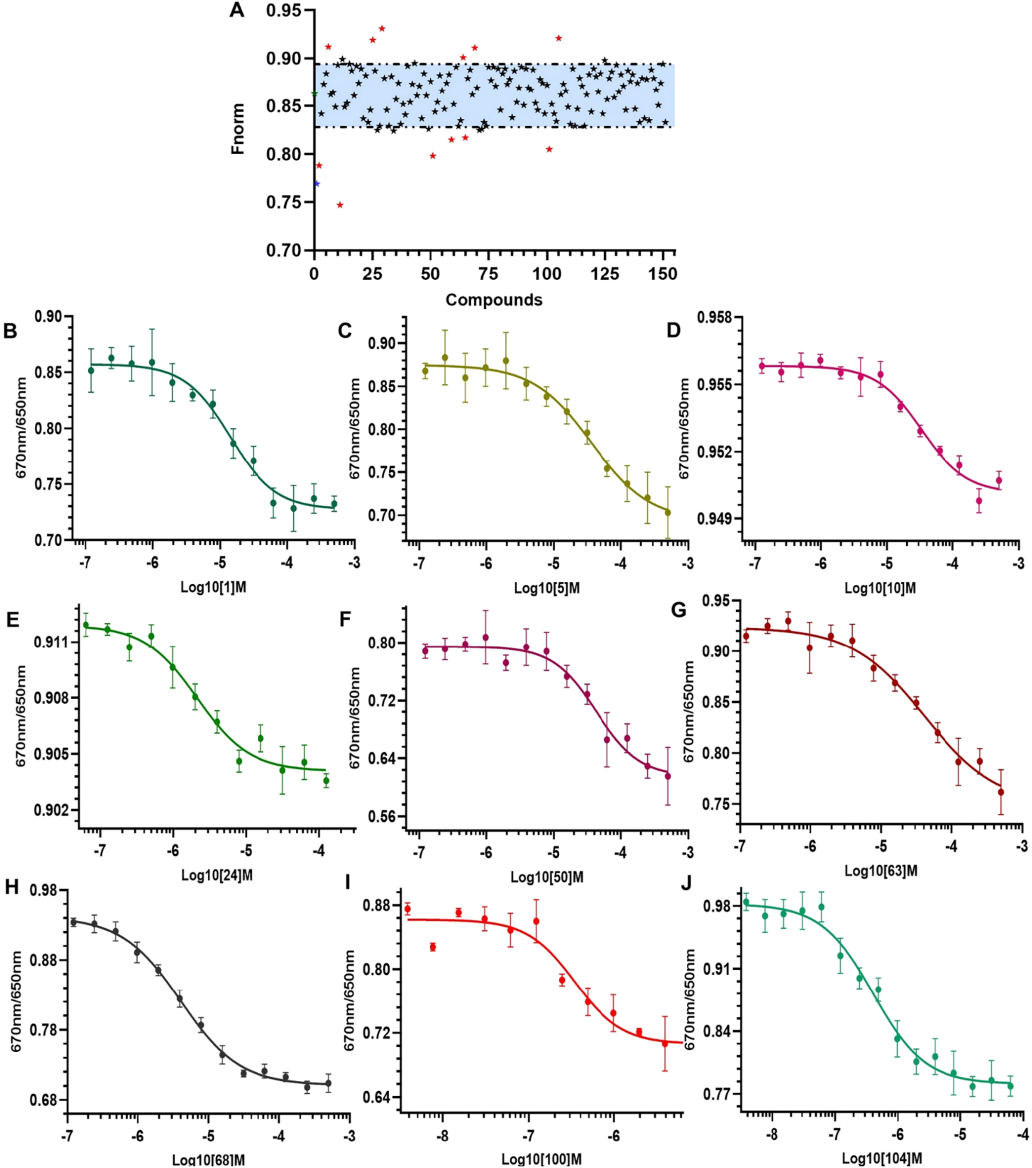
(A) Dianthus-based screening of 150 computationally prioritized compounds using purified CD28 extracellular domain (ECD). Individual compounds are plotted as Fnorm values. The shaded region represents the ±5 SD interval relative to the vehicle control (2% DMSO in PBST, 0.05% Tween). Compounds outside this interval were classified as primary hits. BPU11 is shown as the positive control. (B–J) Concentration–response binding curves obtained by microscale thermophoresis (MST) using purified CD28 ECD. Compounds were tested over a concentration range of 500 μM to 1 nM (1:1 serial dilution) with 2% DMSO maintained across all conditions. Data are presented as mean ± SEM from at least three independent experiments. Solid lines represent nonlinear regression fits used to calculate equilibrium dissociation constants (*K*_d_). (B) **1**, (C) **5**, (D) **10**, (E) **24**, (F) **50**, (G) **63**, (H) **68**, (I) **100**, and (J) **104**.

The 12 primary hits were subsequently advanced to orthogonal biophysical validation by microscale thermophoresis (MST) to quantify direct binding affinity and assess concentration-dependent engagement of CD28.

### Orthogonal Validation of CD28 Binding by MST

To quantitatively validate whether Dianthus/TRIC-derived hits reflected direct molecular engagement of CD28, binding affinities were determined using microscale thermophoresis (MST) with purified CD28 extracellular domain (ECD). MST detects ligand-induced changes in thermophoretic mobility of the labeled protein and was used here as a quantitative affinity assay to derive equilibrium dissociation constants (*K*_d_). Compounds were evaluated over a concentration range spanning 500 μM to 1 nM in a 1:1 serial dilution format, with 2% DMSO maintained across all conditions. Binding measurements were performed in triplicate technical replicates and independently repeated in at least three experiments to ensure reproducibility.

Concentration-dependent thermophoretic shifts yielded well-defined sigmoidal binding curves for multiple compounds. Two candidates, **100** and **104**, exhibited submicromolar affinity with dissociation constants (*K*_d_) of 344 ± 23 nM and 407 ± 15 nM, respectively. **24** and **68** demonstrated low-micromolar binding (*K*_d_ = 2.2 ± 1.4 μM and 4.1 ± 3.2 μM), while 1 bound with moderate affinity (*K*_d_ = 14.2 ± 3.2 μM). Several additional compounds displayed midmicromolar engagement (**5**, **10**, **50**, **63**, **64**, **58**; *K*_d_ ≈ 36–85 μM), whereas **28** showed substantially weaker binding (*K*_d_ = 427 ± 29 μM) (Figures 8B–J and S19).

This orthogonal validation step refined the initial Dianthus hit set into a ranked affinity series, identifying **100** and **104** as the highest-affinity CD28 binders for subsequent functional evaluation. To further assess selectivity, the most potent compound identified in this study (**100**) was evaluated against related immune receptors (ICOS, CD80, and CD86), where no detectable binding was observed under identical conditions (Figure S21). These findings prompted further evaluation of functional activity to determine whether binding translated into disruption of CD28–ligand interactions.

### Competitive Disruption of CD28–CD80 Interaction by Compounds

To evaluate whether MST-confirmed CD28 binders disrupt ligand recognition, all 11 compounds demonstrating dose-dependent binding in MST were assessed in a biochemical CD28–CD80 competition ELISA. Recombinant CD28 was immobilized on 96-well plates and incubated with biotinylated CD80 in the presence of increasing compound concentrations. Inhibition curves were generated from triplicate technical replicates and independently repeated in at least three experiments to derive IC_50_ values.

Seven of the 11 MST-positive compounds exhibited reproducible, concentration-dependent inhibition of CD28–CD80 binding. **100** showed the highest potency (IC_50_ = 231 ± 34 nM), followed by **104** (IC_50_ = 841 ± 113 nM) and **68** (IC_50_ = 1.3 ± 0.8 μM). **1** and **24** displayed moderate inhibition (IC_50_ = 9.5 ± 1.6 μM and 22.3 ± 6.1 μM), whereas **10** showed weaker activity (IC_50_ = 106 ± 13 μM) (Figure 9A–F) and **28** exhibited minimal inhibition (IC_50_ = 411 ± 52 μM) (Figure S20 in Supporting Information).

**Figure 9:**
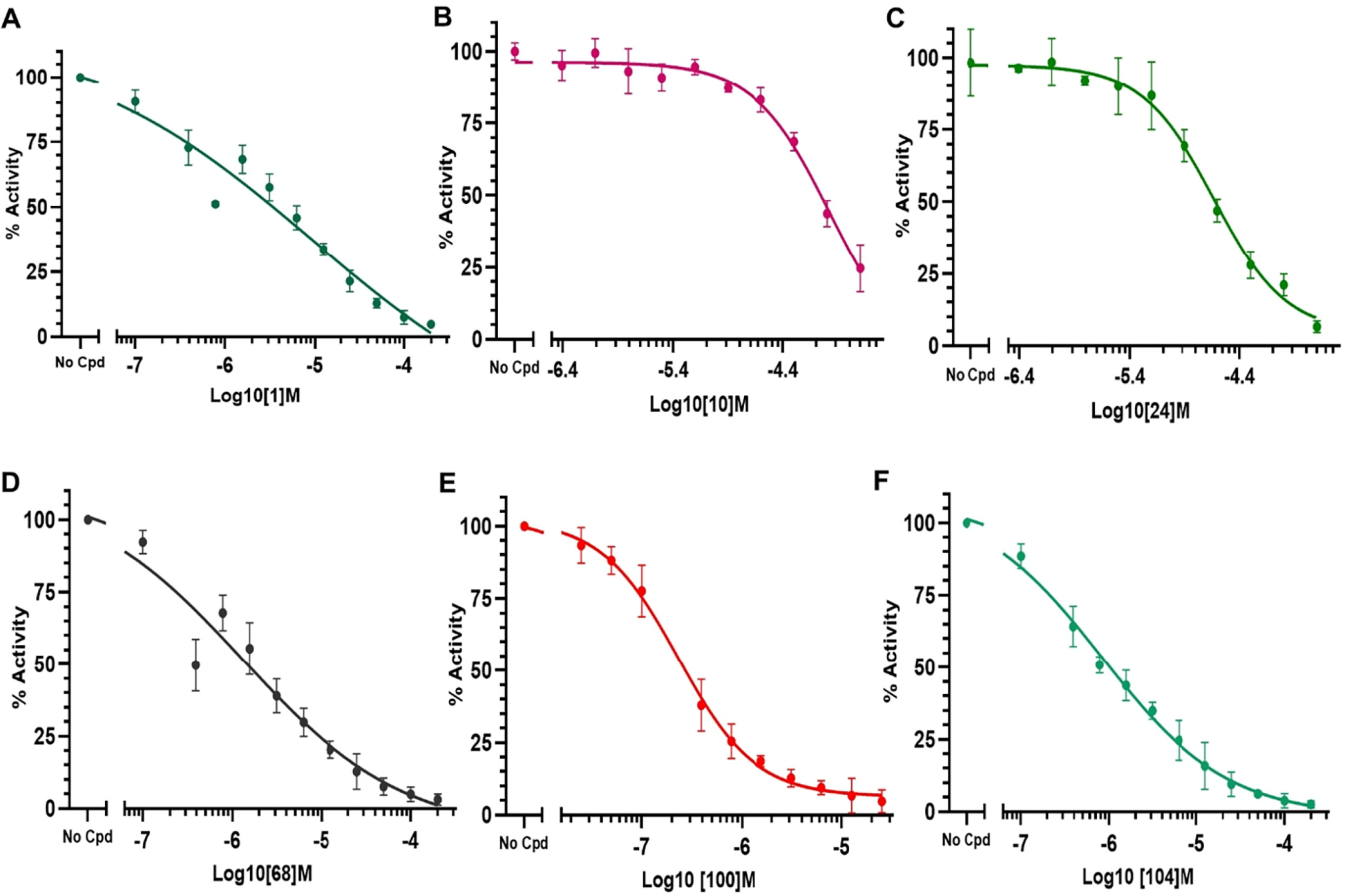
Dose–response curves from ELISA-based CD28–CD80 binding assays. CD28 extracellular domain was immobilized and incubated with biotinylated CD80 in the presence of increasing concentrations of the indicated compounds. Percent activity was calculated relative to the no-compound control (set to 100%). Compounds were tested over a defined concentration range using serial dilutions. Data are presented as mean ± SEM from at least three independent experiments. Solid lines represent nonlinear regression fits used to determine IC_50_ values. (A) **1**, (B) **10**, (C) **24**, (D) **68**, (E) **100**, and (F) **104**.

The remaining MST-confirmed binders did not demonstrate reproducible dose-dependent blockade under identical conditions, indicating that CD28 binding does not uniformly translate into competitive disruption of CD80 engagement. Notably, the most potent functional inhibitors (**100** and **104**) were also the highest-affinity binders identified by MST, revealing a clear correlation between binding strength and competitive activity. Table 1 summarizes the results of the biological evaluation of the positive hits.

**Table 1:**
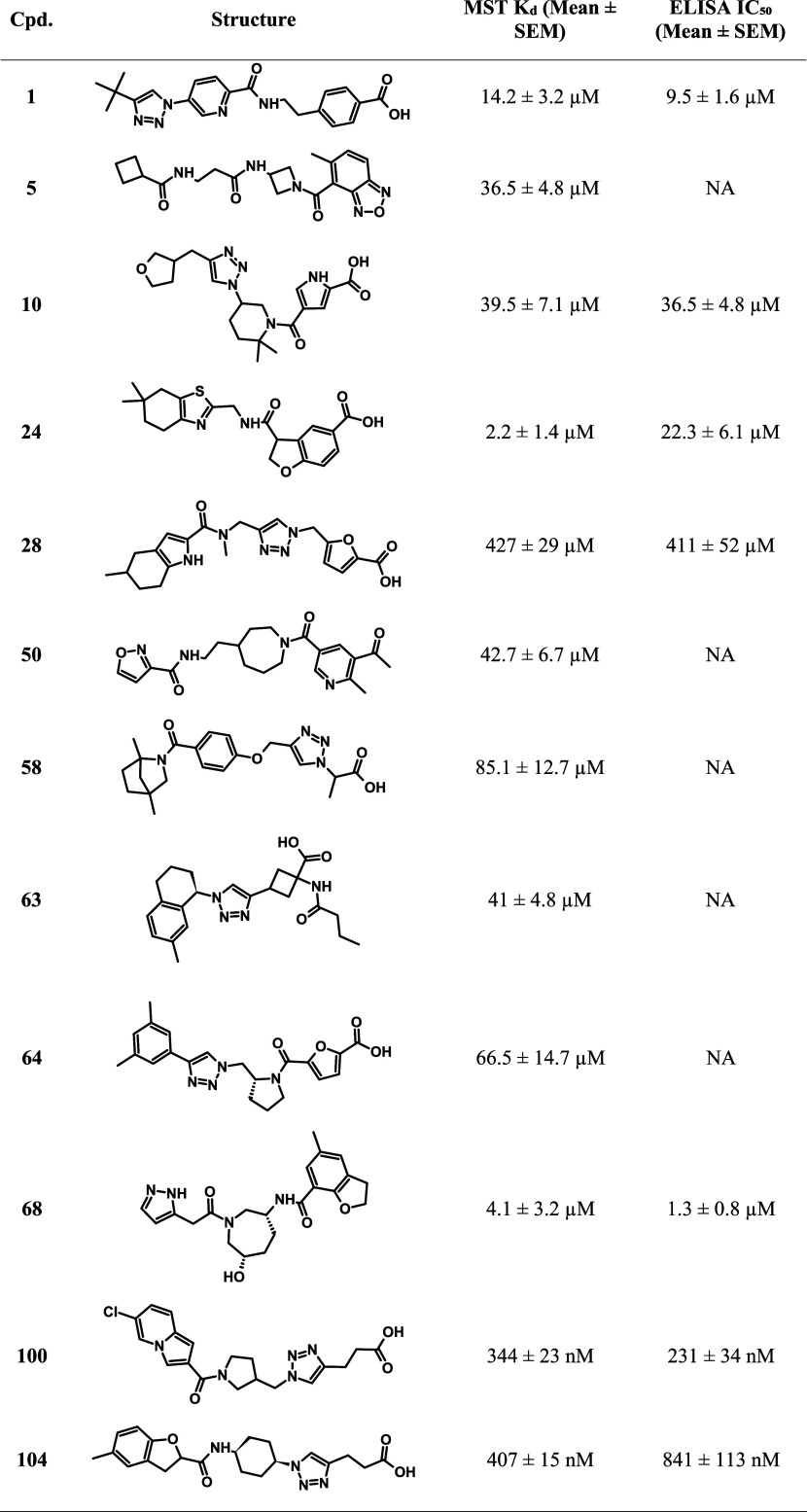
Structures of the Best Compounds from among the 150 Tested Ones, along with Their MST and ELISA *K*_d_, and IC_50_ Values, Respectively

Taken together, these results establish a tiered validation framework in which compounds were systematically progressed and categorized across the screening cascade, from TRIC-identified candidates to MST-confirmed binders and ELISA-active inhibitors, thereby linking direct molecular engagement to functional disruption of the CD28–CD80 interaction and subsequent inhibition of CD28-dependent signaling in cellular systems.

### Binding Mode Analysis

Following the biological evaluation, compounds **100** and **104** emerged as the most active derivatives. Interestingly, these two molecules belong to two distinct clusters among those previously defined based on shape. Specifically, compound **100** belongs to cluster 3 (Figure 7). In this predicted binding mode, the 6-chloroindolizine moiety is oriented toward the H38 side chain (Figure 10A), allowing for the potential formation of halogen bond interactions. The spatial arrangement of this aromatic portion also might allow the formation of favorable cation-π interactions with the K95 side chain. Moreover, the carbonyl group adjacent to the pyrrolidine establishes H-bond interactions with the side chains of K109 and K2. This latter residue side chain might also potentially engage in cation-π interactions with the central triazole ring. Finally, the terminal carboxylic acid forms H-bond interactions with the D106 backbone NH and a Coulombic contact with K2.

**Figure 10:**
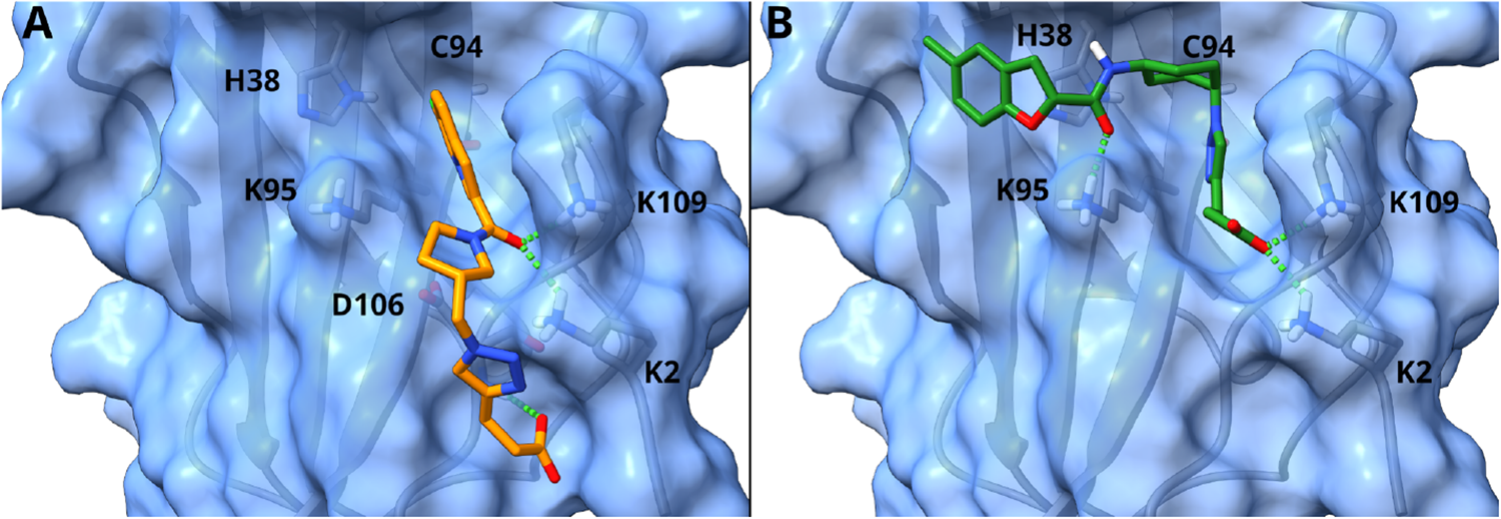
Predicted binding mode of both **100** (A) and **104** (B), orange and green sticks respectively, in complex with the X-ray structure of CD28. The protein is depicted as gray ribbons and sticks and a blue surface.

Conversely, compound **104** was assigned to cluster 1. In its predicted binding pose, the 5-methylbenzofuran group might establish a T-shaped interaction with the H38 side chain, while the cyclohexylamide CO H-bonds with the K95 side chain (Figure 10B). This K95 residue may also mediate cation–π interactions with the triazole core of **104**. Similar to **100**, the terminal carboxylic acid of **104** is involved in strong ionic interactions with both K109 and K2 side chains.

All in all, given the high structural similarity between the two molecules, their convergent interaction profile with the same set of amino acid residues within the targeted pocket, and their comparable biological activities, it is tempting to postulate that both molecules might be able to alternatively adopt either binding mode. Therefore, for future hit-to-lead optimization campaigns, both binding modes (represented by clusters 1 and 3 in Figure 7) might be considered as highly valid and acceptable conformations for the design of novel CD28 binders.

The proposed binding modes are derived from docking-based predictions and should be interpreted as hypothesis-generating rather than experimentally validated structural models. Accordingly, the interaction descriptions above are intended to represent plausible binding hypotheses rather than definitive residue-level assignments. While orthogonal approaches such as site-directed mutagenesis or hydrogen–deuterium exchange could further refine these interactions, the present study was designed to establish functional inhibition through a multitier validation framework. The consistency observed across MST, ELISA, and cellular assays supports direct and functionally relevant engagement of CD28 by these compounds. Future studies incorporating structural approaches such as crystallography or targeted mutagenesis will be valuable to further define residue-level binding interactions.

### Cell-Based Reporter Assay Confirms CD28 Signaling Blockade by Lead Compounds

To assess whether biochemical disruption of CD28–CD80 binding translated into suppression of CD28-dependent signaling in cells, ELISA-active compounds were evaluated using a CD28 Blockade Bioassay. This luciferase-based reporter assay employs Jurkat effector cells cocultured with Raji artificial antigen-presenting cells (aAPCs) and quantitatively measures CD28-mediated costimulatory signaling. Compounds were tested in a dose–response format, and luminescence was recorded after 5 h of incubation. IC_50_ values were derived from triplicate technical replicates and independently repeated in at least three experiments.

Of the seven compounds that showed dose-dependent inhibition in the CD28–CD80 ELISA assay, three demonstrated reproducible, concentration-dependent suppression of CD28-driven reporter activity. **100** exhibited the strongest cellular potency (IC_50_ = 749 ± 124.7 nM), followed by **104** (IC_50_ = 1.13 ± 0.2 μM) (Figure 11B,C). **24** showed weaker but measurable activity (IC_50_ = 60 ± 11 μM) (Figure 11A). The remaining ELISA-active compounds did not exhibit dose-dependent inhibition in this cellular assay, indicating that biochemical competition does not consistently translate into effective blockade of CD28-mediated signaling. Across the screening cascade, **100** and **104** consistently demonstrated high-affinity binding (MST), potent disruption of CD28-CD80 interaction (ELISA), and submicromolar inhibition of CD28-driven reporter signaling, establishing them as the most advanced CD28 antagonists within the series.

**Figure 11:**
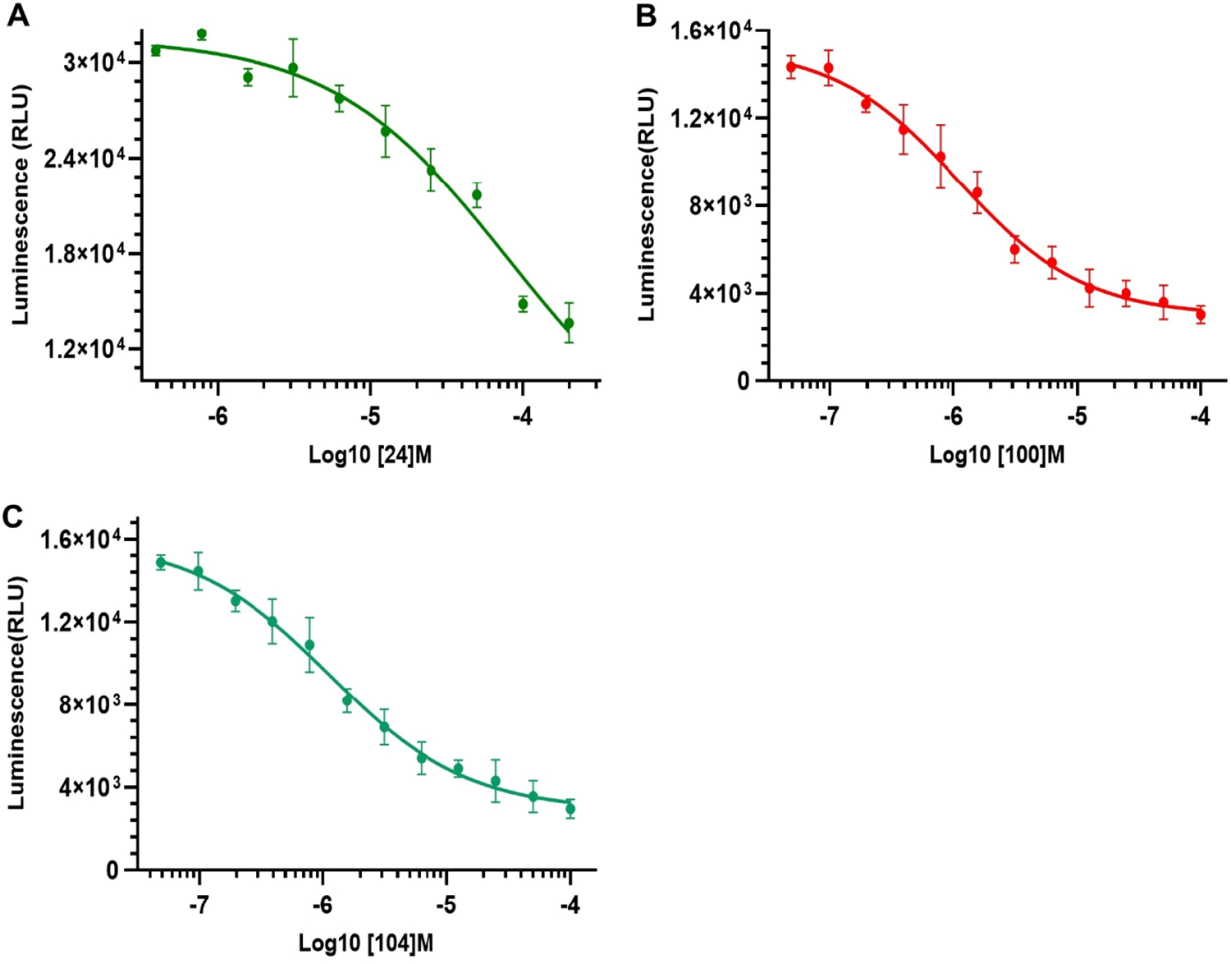
Dose–response curves from a CD28 Blockade Bioassay performed using Jurkat effector cells cocultured with Raji antigen-presenting cells. Luminescence (RLU) was measured following compound treatment over a defined concentration range using serial dilutions. Data are presented as mean ± SEM from at least three independent experiments. Solid lines represent nonlinear regression fits used to determine IC_50_ values. (A) **24**, (B) **100**, and (C) **104**.

### Assessment of T Cell Activation in a Tumor-PBMC 3D Coculture Model

To evaluate the functional impact of our lead CD28 antagonists (**100** and **104**) under physiologically relevant conditions, we employed a 3D coculture system consisting of IFN-γ-conditioned A549 tumor spheroids combined with primary human PBMCs. T cell receptor signaling was initiated using submaximal anti-CD3 stimulation to ensure that downstream activation remained dependent on CD28-mediated costimulatory signals provided by tumor-associated CD80/CD86.

This setup produced strong T cell activation (Figure 12A,B), as demonstrated by increased release of hallmark cytokines including IFN-γ, IL-2, and soluble CD69. Both **100** and **104** attenuated cytokine production in a dose-responsive manner (1, 5, and 10 mM), confirming effective interference with CD28-driven costimulation. Across all measured end points, **100** showed slightly greater inhibitory potency than **104**, in agreement with its superior biochemical activity profile. The clinical-stage CD28 antagonist FR104 was included as a benchmark control to contextualize the activity of newly identified compounds in physiologically relevant systems. The use of BPU11 and FR104 across different assay tiers reflects their distinct roles, with BPU11 serving as a screening-level reference control and FR104 providing a clinically relevant benchmark for functional inhibition in cellular systems. Collectively, these findings demonstrate that both compounds suppress CD28-dependent T cell activation within a human tumor-immune microenvironment model, with **100** representing the more advanced candidate for further development.

**Figure 12:**
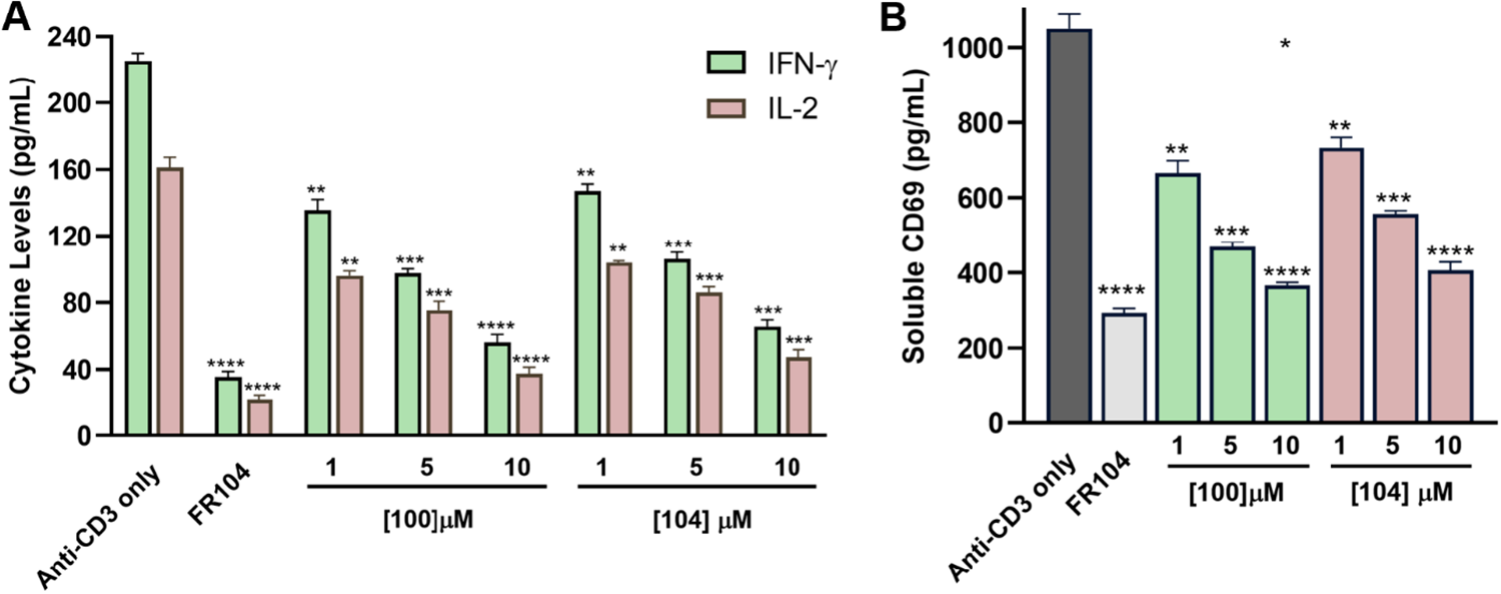
Concentration-dependent suppression of T cell activation in a tumor-PBMC coculture model. (A) IFN-γ and IL-2 concentrations were measured in supernatants collected after 48 h of coculture between A549 tumor spheroids and human PBMCs (effector-to-target ratio 5:1). Cultures were stimulated with anti-CD3 (0.3 μg/mL) and treated with FR104 (10 μg/mL), **100** (1, 5, or 10 μM), or **104** (1, 5, or 10 μM). (B) Soluble CD69 levels were determined by ELISA under identical experimental conditions following 48 h of coculture. Data are presented as mean ± SD from three independent wells. Statistical analysis was conducted relative to the anti-CD3-stimulated control group using one-way ANOVA with Dunnett’s multiple comparison test. Significance levels are indicated as follows: **p < 0.01; ***p < 0.001; ****p < 0.0001 versus anti-CD3 alone.

### Human PBMC-Airway Tissue Coculture Model

To further examine the biological activity of **100** (the most potent lead from this study) in a physiologically relevant setting, we implemented a human coculture platform designed to mimic immune-epithelial interactions at mucosal surfaces. In this system, differentiated airway epithelial tissues (MucilAir, Epithelix) were combined with primary human PBMCs and activated using plate-bound anti-CD3 together with soluble anti-CD28 to induce costimulatory signaling. This configuration preserves important characteristics of the mucosal immune niche, including epithelial barrier function, localized cytokine signaling, and bidirectional communication between immune and epithelial compartments. Cytokines were measured from the apical supernatant after 48 h to determine the impact of CD28 inhibition.

Dual CD3/CD28 stimulation triggered strong production of IFN-γ, IL-2, and TNF-α, reflecting robust T cell activation. Administration of **100** (1, 5, and 10 mM) attenuated cytokine release in a concentration-dependent manner across the three tested doses (Figure 13A–C). FR104 was included as a reference inhibitor to confirm assay responsiveness. Collectively, these data indicate that **100** retains potent inhibitory activity in a primary human mucosal model, consistent with its performance in tumor-PBMC cocultures, supporting effective CD28 blockade across diverse immune microenvironments.

**Figure 13:**
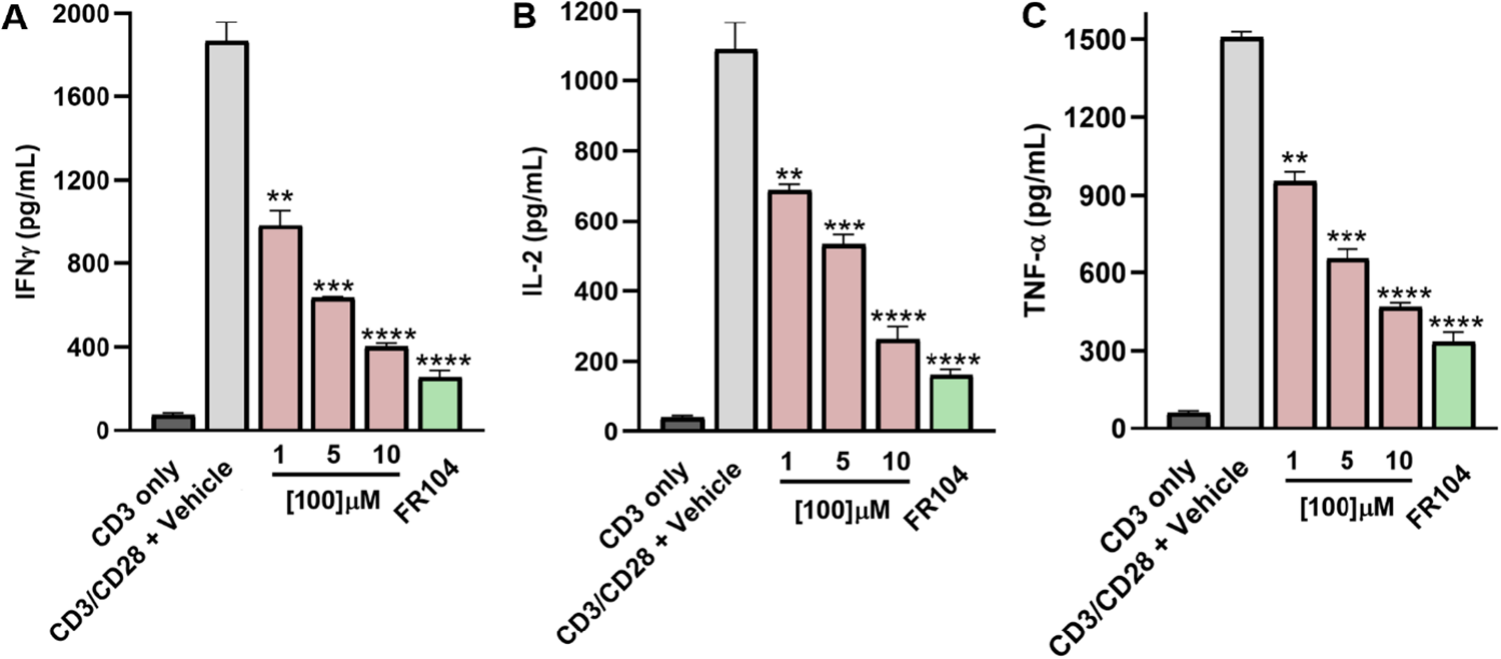
Inhibition of CD28-driven cytokine production by **100** in a human PBMC-airway tissue coculture system. Primary human PBMCs were applied to differentiated MucilAir airway epithelial inserts (Epithelix) and stimulated for 48 h with plate-immobilized anti-CD3 together with soluble anti-CD28. Treatments included vehicle control (0.1% DMSO), **100** at 1, 5, or 10 μM, or the reference CD28 antagonist FR104 (10 μg/mL). Levels of IFN-γ (A), IL-2 (B), and TNF-α (C) in apical supernatants were measured by ELISA and expressed in pg/mL. **100** reduced cytokine secretion in a concentration-dependent fashion, with maximal inhibition at 10 μM, approaching the effect observed with FR104. Statistical analysis was performed relative to the CD3/CD28-stimulated vehicle control using one-way ANOVA followed by Dunnett’s multiple comparison test. Significance thresholds are indicated as **p < 0.01, ***p < 0.001, and ****p < 0.0001 versus CD3/CD28 + vehicle.

## Conclusions

In this study, we successfully addressed the challenge of targeting the shallow, solvent-exposed protein–protein interaction interface of CD28 using small molecules. By taking advantage of the PyRMD2Dock suite developed by some of us, we coupled the screening capabilities of an ML method with the predictive power of docking-based VS, enabling the rapid and efficient virtual screening of 48 million compounds from the Enamine REAL Diversity Set. This represents the first real-world, prospective application of this AI-enforced pipeline. Employing a tailored filtering strategy, a set of 150 candidates was prioritized for experimental evaluation. As a result, we identified multiple direct CD28 binders, notably compounds **100** and **104**, which exhibited submicromolar affinities (*K*_d_ = 343.78 nM and 407.08 nM, respectively). These findings highlight the critical advantage of mining ultralarge chemical spaces to identify high-affinity ligands.

These hits demonstrated potent competitive disruption of the CD28–CD80 interaction and efficient blockade of the CD28-mediated signaling in cellular reporter assays. Crucially, the biological efficacy of these compounds translated into complex, physiologically relevant 3D coculture systems. Both compounds significantly attenuated T cell activation and cytokine secretion in a tumor-PBMC model, while **100** also showed potent inhibitory activity in a mucosal epithelial-PBMC platform. Furthermore, computational analysis revealed that the highly active compounds **100** and **104** should occupy the target cleft in distinct, equally valid binding orientations, offering valuable insights for future hit-to-lead optimization campaigns. Ultimately, the discovery of these potent CD28 antagonists validates the PyRMD2Dock platform. This study demonstrates that AI-driven, large-scale virtual screening can successfully unlock structurally demanding immune receptor interfaces, providing a scalable and highly effective paradigm for targeting complex PPIs.

## Material and Methods

### Molecular Docking

X-ray structure of CD28 in complex in complex with the Fab fragment of a mitogenic antibody (1YJD)^6^ was sourced from the RCSB PDB database and underwent preliminary adjustments for docking purposes using the protein preparation wizard integrated into the Schrödinger suite.^6,19^ The hydrogen atoms were added and minimized, the solvent molecules were removed, and the appropriate protonation and tautomeric state of the protein’s side chains were calculated at physiological pH. Using the AutoDockTools Python scripts, the CD28 structure was converted into the AutoDock PDBQT format, where, compared to a standard PDB file, Gasteiger charges are added to the atoms, and the torsional freedoms of the various bonds are described.^20^ Then, the receptor grid maps were calculated with the AutoGrid4 software, mapping the receptor interaction energies using every AutoDock atom type as a probe, and the docking grid box was centered on the binding site made by H38, F93, K95, D106, N107, K109, and S110 residues (*XYZ* size of the box: 60 × 60 × 60 with a spacing of 0.375 Å). From the ERDS, a set of 2.4 M compounds was selected, and the molecules’ SMILES strings were converted into 3D structures with the employment of the LigPrep routine in Schrödinger’s Maestro suite.^21^ After, their tautomeric and protonation states were calculated at physiological pH, and the possible enantiomers were generated. The prepared molecules were exported as PDB files and converted into PDBQTs by making use of the AutoDock suite scripts. For the docking calculations, attained through AD4-GPU, the Lamarckian Genetic Algorithm (LGA) was employed, encompassing a total of 50 LGA runs.^14^ All other settings were maintained at their default values. The docking results were subsequently grouped based on the RMSD criterion, whereby solutions differing by less than 2.0 Å were considered part of the same cluster. The ranking of these clusters was determined based on the calculated free energy of binding (Δ*G*_AD4_).

### AI-Enforced Virtual Screening

To create a ML prediction model, PyRMD was fed with a comma-separated file (.csv) generated by extracting the predicted lowest Δ*G*_AD4_ for each of the 2.4 million docked compounds extracted from the ERDS ultralarge database.^13^ This led to the construction and preparation of the training data set. According to their predicted Δ*G*_AD4_, the compounds included in the. csv file were classified into three groups: “actives”, “inactives”, and discarded. Compounds whose predicted Δ*G*_AD4_ falls below the “activity” thresholds of −8.25, −8.0, −7.75, and −7.50 kcal/mol were placed in the active group. Instead, those with a Δ*G*_AD4_ value higher than the “inactivity” threshold of −4.75, −4.50, and −4.25 kcal/mol went into the “inactives” group. Moreover, compounds whose Δ*G*_AD4_ value falls above the “activity” threshold and “below-the “inactivity” threshold were discarded. By selecting the MinHash fingerprints (MHFP) with a vector length of 2048 units for the featurization process, and by varying the ε cutoff for “actives” (0.70–0.99 with a 0.05 step) and “inactives” (0.60–0.95 with a 0.05 step), 672 different models were generated.^22^ During model training and validation, PyRMD Studio automatically applied Butina clustering coupled with a stratified approach to separate structural clusters between training and test sets. This rigorous data splitting strategy prevented data leakage and ensured a realistic evaluation of the models’ true generalization capabilities.

Across all models, PyRMD returns relevant metrics to evaluate their predictive performance (i.e., TPR, FPR, F1-Score, ROC AUC, BED ROC, PRC AUC). In this work, the selected model was chosen by maximizing the TPR/FPR trade-off alongside robust Precision and F-score metrics. Once the model was generated, PyRMD was used to screen the remaining ∼46 million compounds from the ERDS database and it automatically returned all the compounds deemed to be active along with a confidence score of its prediction (RMD Score).

### Compound Selection and Procurement

Following the computational pipeline described previously (see Results and Discussion section), a total of 150 compounds from the ERDS ultralarge database were selected for experimental evaluation. From this set, all the compounds were synthesized on demand by Enamine (Kyiv, Ukraine) from the REAL Space collection. All compounds were supplied with reported purity ≥90% (LC–MS verified by the supplier) and were used without further purification. Structural identities were confirmed based on supplier-provided analytical data (HPLC and MS). HPLC spectra and ^1^H NMR characterization data for all compounds are provided in the Supporting Information (pp. S2–S11). SMILES, purity information, and vendor codes for all compounds used in this study are listed in the accompanying Sl Excel file.

### Screening of Small-Molecule Binders to Human CD28 Using the Dianthus TRIC Assay

His-tagged recombinant human CD28 extracellular domain (ECD) was obtained commercially and labeled using RED-tris-NTA second Generation dye (NanoTemper Technologies) according to the manufacturer’s instructions. Briefly, CD28 protein was incubated with dye at a 1:0.5 molar ratio in assay buffer (PBS, pH 7.4, supplemented with 0.05% Tween-20) for 30 min at room temperature in the dark.

For primary screening, labeled CD28 was mixed 1:1 with compounds to yield a final compound concentration of 100 μM in 2% DMSO.^23,23^ Samples were incubated for 15 min at room temperature prior to measurement. 2% DMSO served as the negative control, and BPU11 was included as a reference compound. After brief centrifugation (1000*g*, 1 min), 20 μL of each sample was loaded into premium capillaries and analyzed using the Dianthus NT.23 Pico instrument (NanoTemper Technologies) at 25 °C.

Fluorescence at 670 nm was recorded for 5 s before and 5 s after IR-laser activation. Normalized TRIC signals (Fnorm) were calculated as the ratio Fhot/Fcold. Although a *Z*′ factor was not calculated, a ±5 standard deviation cutoff from the vehicle control was used to define hits. Each compound was tested in triplicate technical replicates, and control samples were distributed throughout the 384-well plate to monitor signal stability.

### Microscale Thermophoresis (MST)–Based Binding Affinity Measurements

Binding affinities were determined using microscale thermophoresis (MST) on a Monolith X instrument (NanoTemper Technologies), using spectral shift detection mode. His-tagged recombinant human CD28 extracellular domain (Acro Biosystems) was labeled with RED-tris-NTA second Generation dye according to the manufacturer’s instructions. Labeling was performed in assay buffer (PBS, pH 7.4, containing 0.05% Tween-20) and incubated for 30 min at room temperature in the dark.

Compounds were prepared as serial dilutions spanning concentrations from 500 μM to 1 nM in assay buffer containing 2% DMSO. Labeled CD28 (100 nM protein labeled with 50 nM dye) was mixed 1:1 with compound dilutions, resulting in a final protein concentration of 50 nM, and incubated for 15 min at room temperature in the dark before measurement.

Samples were loaded into Monolith premium capillaries and measured at 25 °C. Binding was quantified by monitoring ligand-induced changes in the fluorescence emission ratio (F670/F650) upon IR-laser excitation. Normalized fluorescence ratio values were plotted against compound concentration to generate binding curves.

Each compound was tested in triplicate technical replicates, and binding experiments were independently repeated at least three times. Dissociation constants (*K*_d_) were determined by nonlinear regression using MO. Affinity Analysis software and GraphPad Prism (four-parameter logistic model).

### Evaluation of CD28–CD80 Binding Inhibition by Competitive ELISA

Competitive ELISA assays were performed using a CD28–CD80 binding assay kit (BPS Bioscience) according to the manufacturer’s instructions with minor modifications. Briefly, 96-well plates precoated with recombinant human CD28 were equilibrated to room temperature and washed with 1× wash buffer before use.^8,24^

Serial dilutions of test compounds were prepared in assay buffer containing 2% DMSO. Compounds were coincubated with biotinylated human CD80 for 1 h at room temperature to allow competitive binding to immobilized CD28. Wells treated with vehicle (2% DMSO) served as negative controls, and wells containing inhibitor buffer without compound were used to define maximal binding.

Following incubation, wells were washed and incubated with streptavidin–HRP diluted in blocking buffer for 1 h at room temperature. After washing, chemiluminescent substrate was added, and luminescence was immediately measured using a microplate reader.

Percent inhibition was calculated relative to vehicle control wells. IC_50_ values were determined by fitting concentration–response curves using a four-parameter logistic regression model in GraphPad Prism. All experiments were performed in triplicate technical replicates and independently repeated at least three times.

### CD28 Blockade Bioassay (Luciferase Reporter Assay)

Functional inhibition of CD28-mediated signaling was assessed using the CD28 Blockade Bioassay (Promega, Cat. #JA6101) according to the manufacturer’s instructions. Jurkat CD28 Effector Cells (2 × 10^4^ cells per well) were seeded in white 96-well plates and preincubated with serial dilutions of test compounds prepared in assay medium containing 1% DMSO.^7^

Compounds were tested in a concentration–response format using serial dilutions spanning the indicated concentration range. After compound preincubation (5 min at 37 °C), Raji artificial antigen-presenting cells (aAPCs; 2 × 10^4^ cells per well) were added to initiate CD28-dependent costimulatory signaling. Plates were incubated for 5 h at 37 °C in a humidified 5% CO_2_ atmosphere.

Following incubation, Bio-Glo Luciferase Reagent (Promega) was added according to the manufacturer’s protocol, and luminescence was measured using a microplate luminometer. Dose–response curves were generated using GraphPad Prism (four-parameter logistic regression model), and IC_50_ values were calculated accordingly. Each compound was tested in technical replicates, and experiments were independently repeated. Data are presented as mean ± SEM.

### Assessment of T Cell Activation in a Tumor–PBMC Coculture Model

To examine the concentration-dependent effects of CD28 blockade in a translationally relevant human system, we utilized a three-dimensional tumor–PBMC coculture assay. Tumor spheroids were established by plating A549 cells (from ATCC) into ultralow attachment 96-well plates and allowing aggregation for 48 h. Spheroids were subsequently primed with recombinant human interferon-γ (50 ng/mL) for 24 h prior to immune cell introduction.

Cryopreserved human peripheral blood mononuclear cells (PBMCs) from STEMCELL Technologies were thawed and added to the spheroids at an effector-to-target ratio of 5:1. T cell receptor stimulation was initiated using soluble anti-CD3 antibody (clone OKT3, 0.3 μg/mL). Test compounds were introduced at the onset of coculture. FR104 (10 μg/mL), an anti-CD28 Fab′ fragment, was included as a reference inhibitor. Compounds **100** and **104** were tested at 1, 5, and 10 μM, with vehicle and anti-CD3-only conditions serving as controls.

After 48 h, culture supernatants were collected for cytokine quantification. IFN-γ and IL-2 levels were measured using human ELISA kits (BioLegend), and soluble CD69 was determined using a human CD69 ELISA kit (Thermo Fisher Scientific).

### CD28-Mediated Cytokine Production in a Human PBMC–Airway Tissue Coculture System

To investigate the immunomodulatory effects of **100** within a physiologically relevant mucosal setting, a human airway epithelial tissue model (MucilAir, Epithelix) was cocultured with PBMCs. Cryopreserved PBMCs (StemCell Technologies) were thawed and allowed to recover for 4 h in RPMI-1640 supplemented with 10% heat-inactivated human AB serum, 2 mM l-glutamine, and 1% penicillin-streptomycin.

MucilAir inserts were preconditioned for 24 h in proprietary maintenance medium at 37 °C under 5% CO_2_. PBMCs were adjusted to a density of 2 × 10^6^ cells/mL, and 200 μL was applied to the apical compartment of each insert in 24-well Transwell plates. T cell activation was induced using plate-immobilized anti-CD3 antibody (1 μg/mL) together with soluble anti-CD28 (1 μg/mL). Treatments included vehicle control (0.1% DMSO), **100** at 1, 5, or 10 μM, or the reference CD28 inhibitor FR104 (10 μg/mL).

After 48 h of incubation, apical supernatants were collected and analyzed for IFN-γ, IL-2, and TNF-α using human ELISA kits (BioLegend). Cytokine levels were calculated from standard curves and reported in pg/mL. All experimental conditions were conducted in triplicate across three independent experiments.

## Supplementary Material





## Data Availability

The PyRMD Studio software can be downloaded free of charge at https://github.com/cosconatilab/PyRMD-Studio.

## Notes

Chemical structures and SMILES strings of the active compounds, HPLC purity, NMR spectra, MST binding studies, and competitive ELISAS (PDF)
MILES strings, Catalog IDs, and purity for all compounds (XLSX)

## Abbreviations

PPI: protein–protein interaction
AI: artificial intelligence
VS: virtual screening
SBVS: structure-based virtual screening
LBVS: ligand-based virtual screening
ML: machine learning
AD4-GPU: AutoDock-GPU
ECFP6: Extended Connectivity Fingerprints with a radius of 3
ERDS: Enamine REAL Diversity Set
SASA: solvent-accessible surface area
ECD: extracellular domain
MST: microscale thermophoresis
aAPCs: artificial antigen-presenting cells
PBMC: peripheral blood mononuclear cells
LGA: Lamarckian Genetic Algorithm
MHFP: MinHash fingerprints
TPR: True positive rate
FPR: False positive rate
TRIC: temperature-related intensity change
