# Supplementary material for: AI-Enforced Ultra-Large Virtual Screening Discovers Potent CD28 Binders
Source: J Chem Inf Model. 2026 Jun 11;66(15):8805–19. doi: 10.1021/acs.jcim.6c00859 (PMC13471729; doi:10.1021/acs.jcim.6c00859)
Supplement: Supplementary file 1 [file ci6c00859_si_001.pdf]

Supporting Information

AI-Enforced Ultra-Large Virtual Screening Discovers Potent CD28 Binders

Saurabh Upadhyay, [a‡] *Michele Roggia,*[b‡] *Shaoren Yuan,*[a] *Sandro Cosconati*[b*] *and Moustafa T. Gabr*[a*]

[a] Department of Radiology, Molecular Imaging Innovations Institute (MI3), Weill Cornell Medicine, New York, NY 10065, USA.

[b] DiSTABiF, University of Campania Luigi Vanvitelli, 81100 Caserta, Italy

‡ These authors contributed equally to this work

Table of Content:

S. No.	Content	Page Number
Table S1	Structures and vendor codes for all the purchased compounds.	S2-S10
Figure S1	Distribution plot of the 2.4M molecules docked for training purposes.	S10
Fig S2-S13	HPLC Spectra for Active compounds	S11-S14
Fig S14-S19	1H-NMR Spectra for Active compounds	S15-S18
Fig S20	Spectral shift binding curves of **28** and **58** to CD28.	S18
Fig S21	ELISA dose–response curve of **28**	S19
Fig S22	MST-Based Selectivity Assessment of Compound **100**	S19

Table S1. Structures and vendor codes for all the purchased compounds.

Compound#	Structure	Vendor ID
1	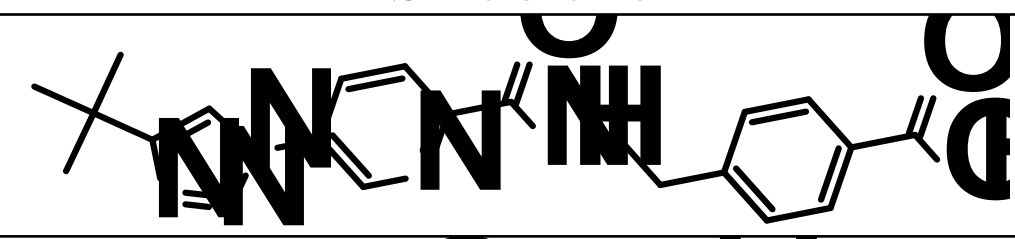	Z9698696370
2	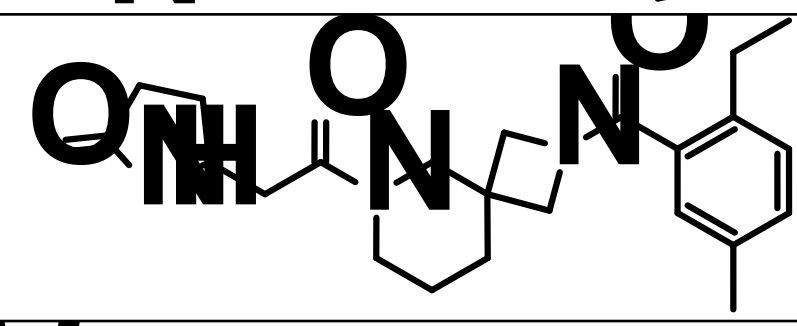	Z9698696424
3	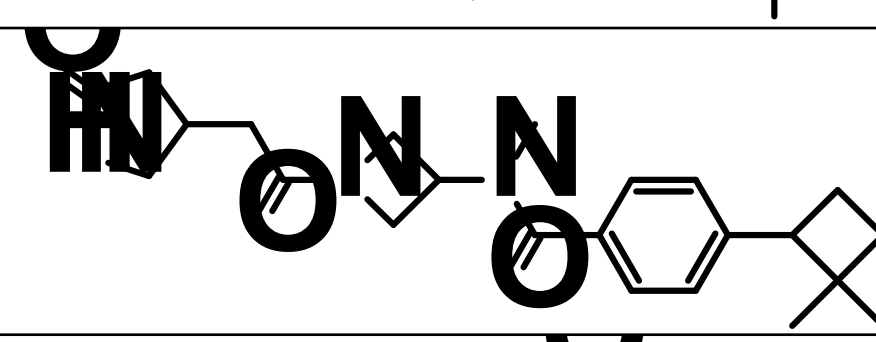	Z9698696369
4	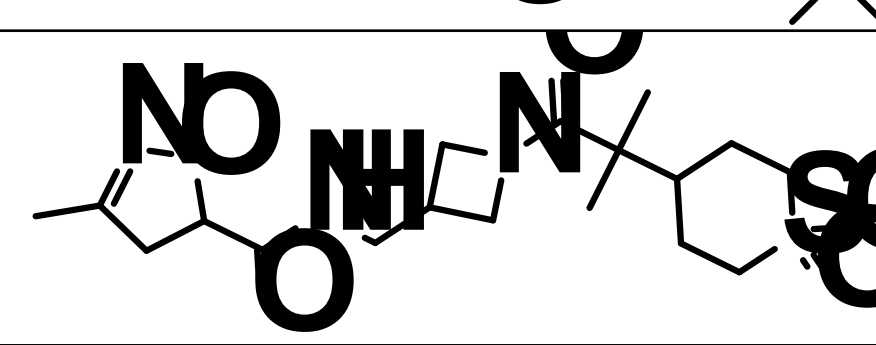	Z9698696308
5	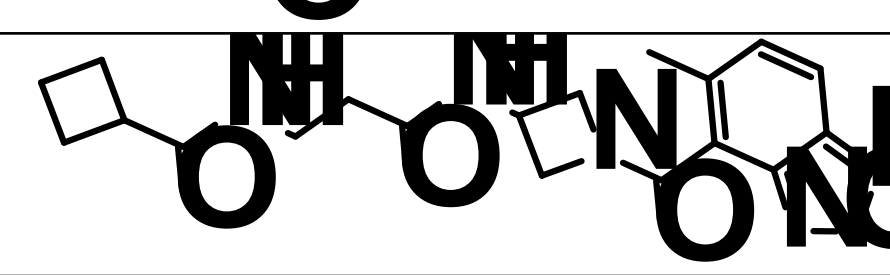	Z9698696375
6	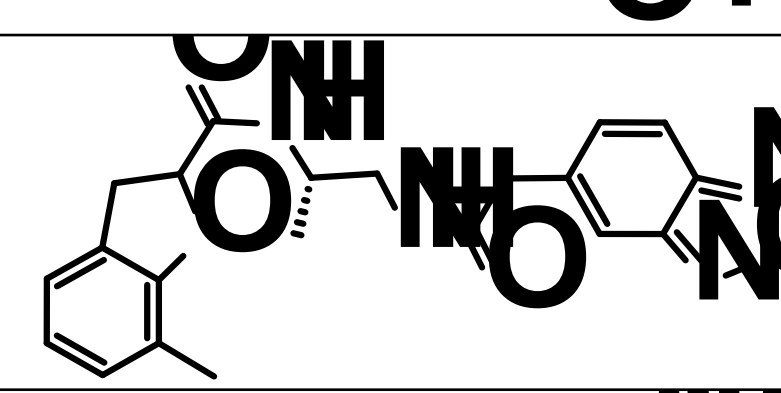	Z9698696371
7	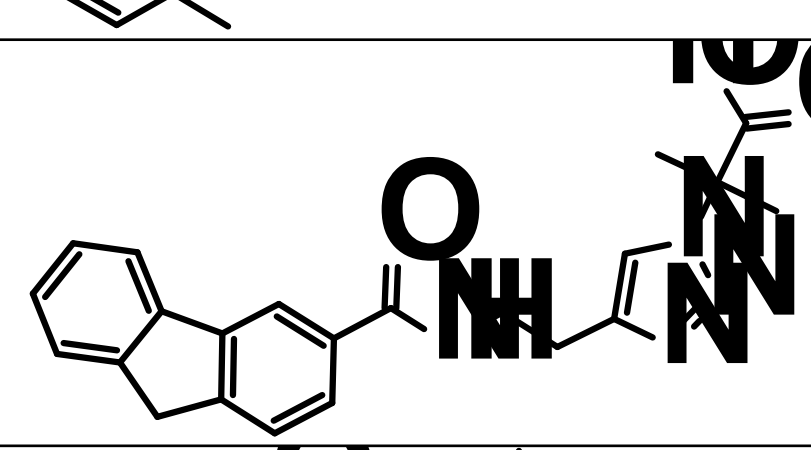	Z9698696376
8	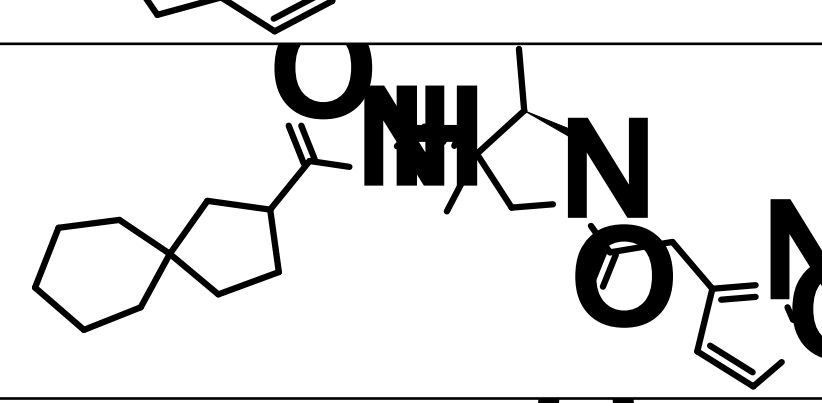	Z9698696298
9	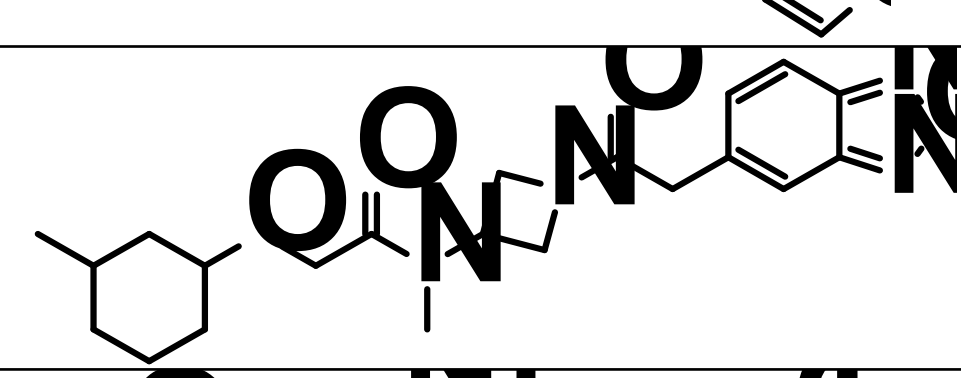	Z9698696395
10	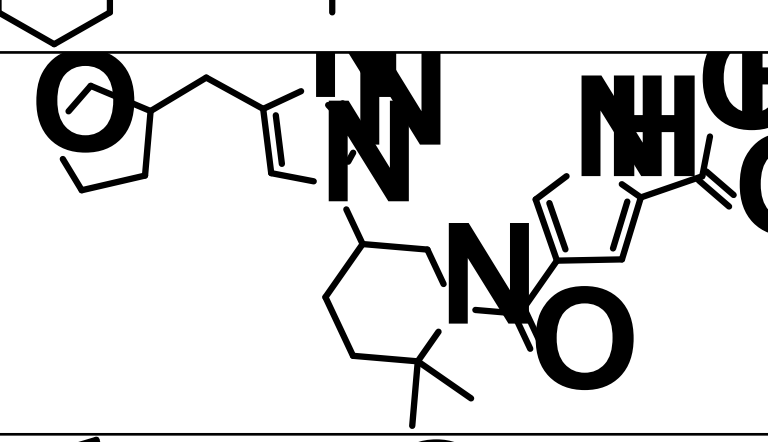	Z9698696401
11	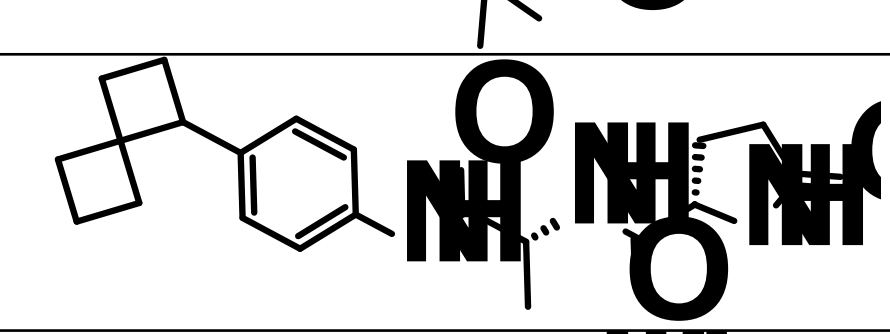	Z7226927075
12	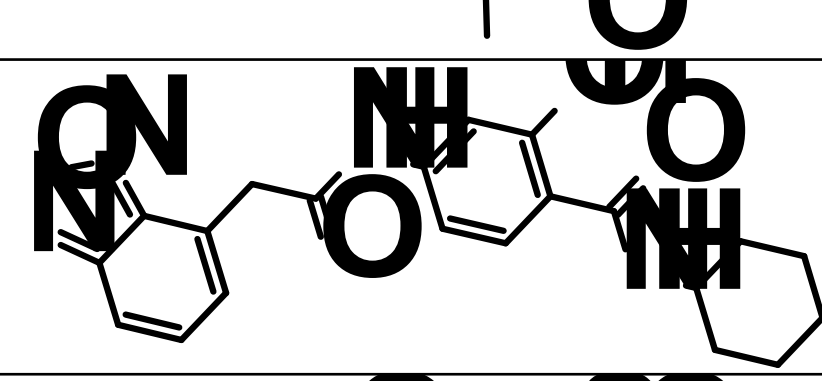	Z9698696382
13	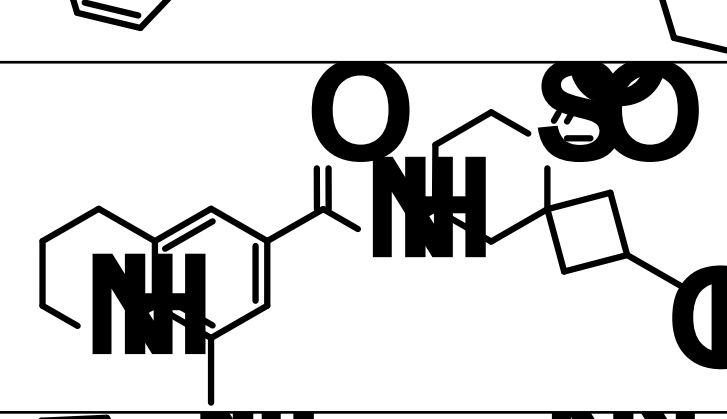	Z6327159723
14	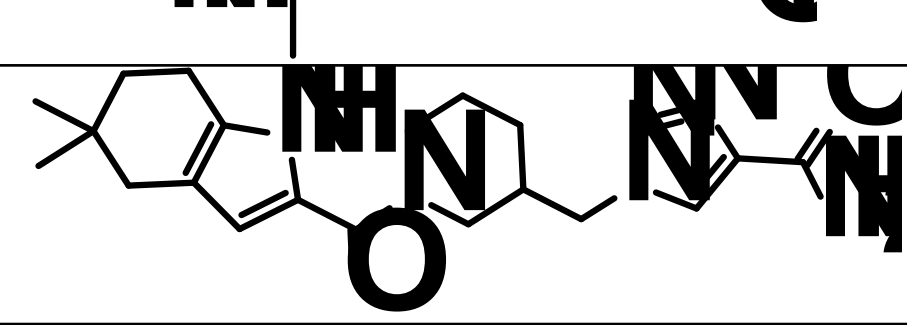	Z9698696385
15	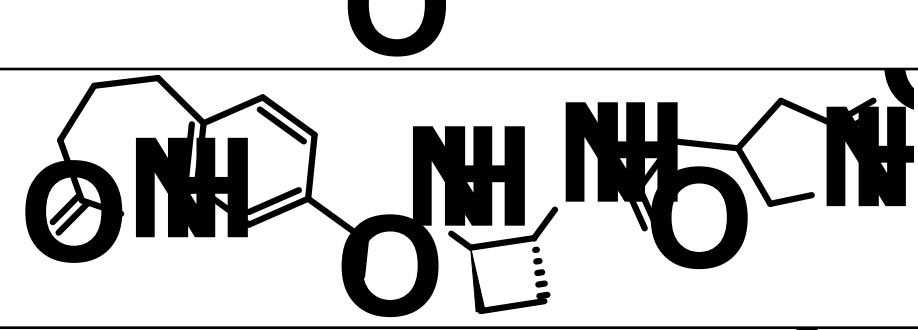	Z9698696394
16	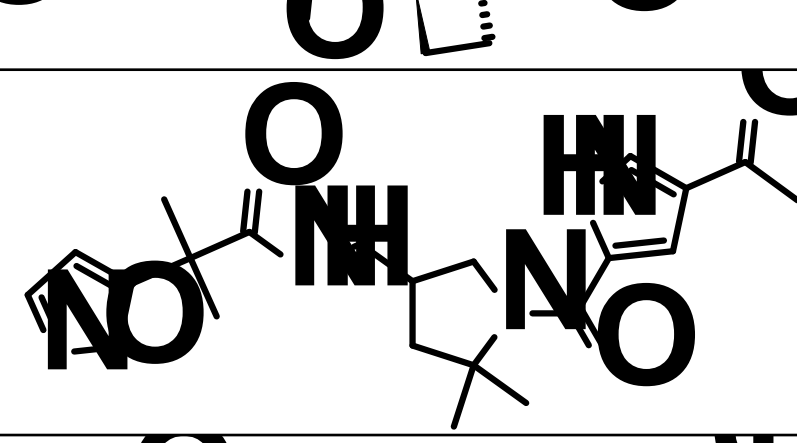	Z9698696409
17	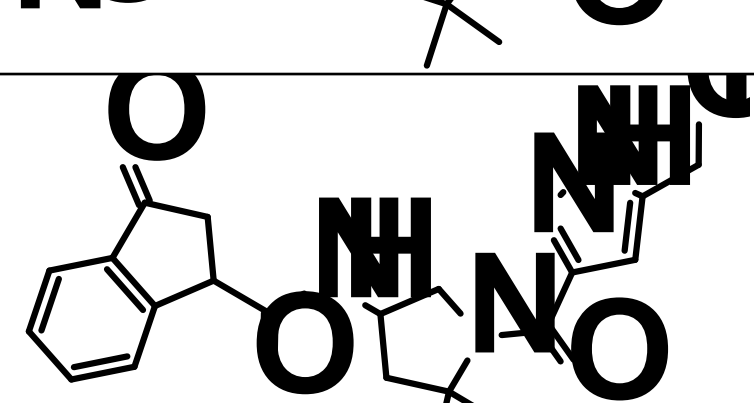	Z9698696412

**18**	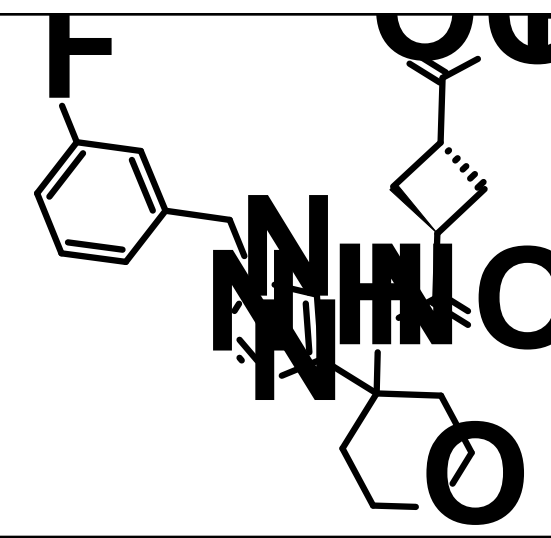	Z9698696408
**19**	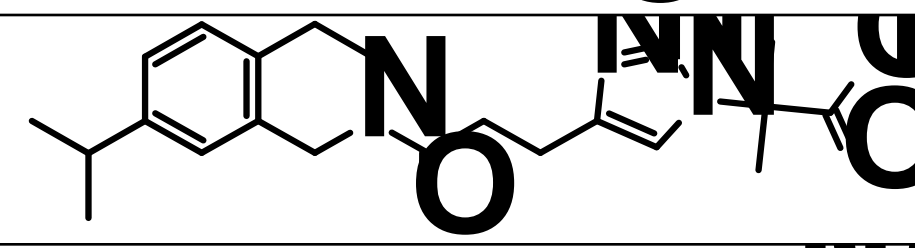	Z9698696379
**20**	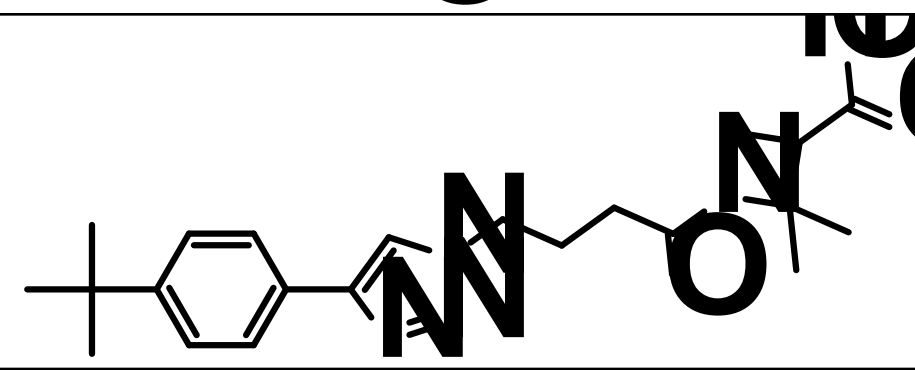	Z9698696358
**21**	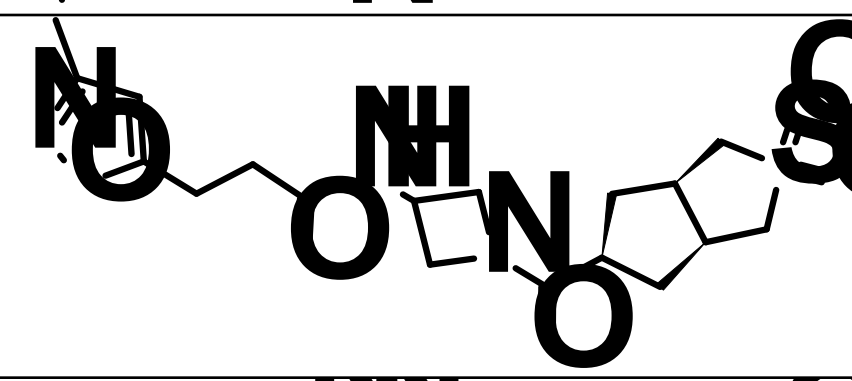	Z9698696377
**22**	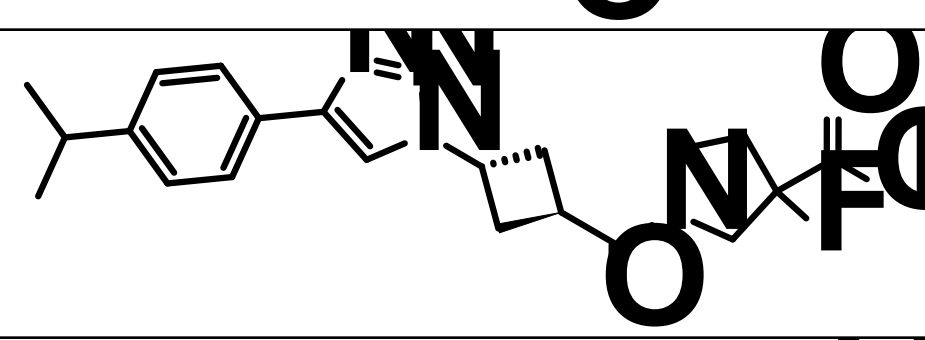	Z9698696344
**23**	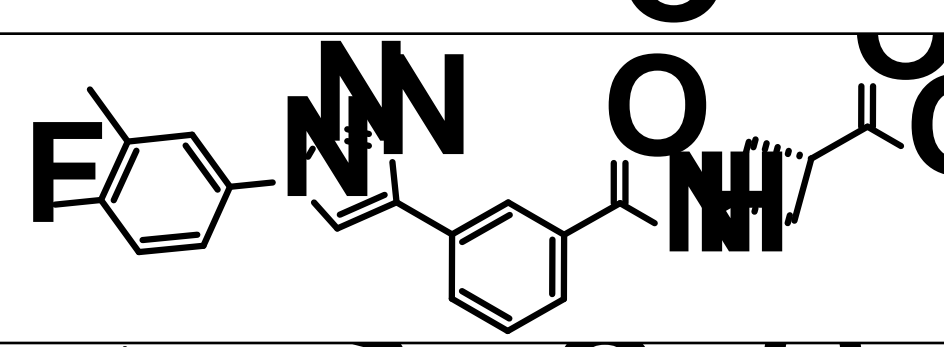	Z9698696350
**24**	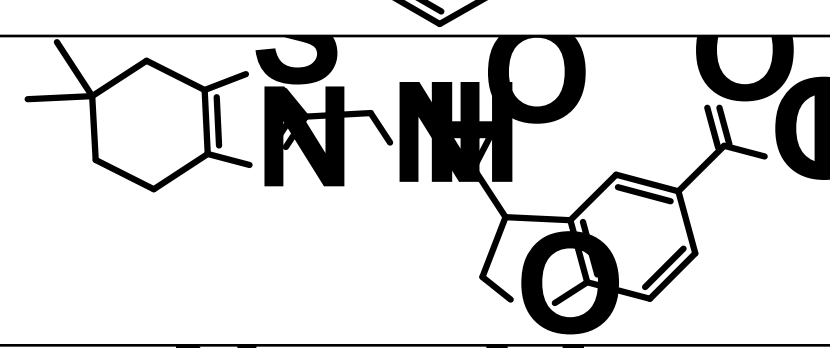	Z9698696341
**25**	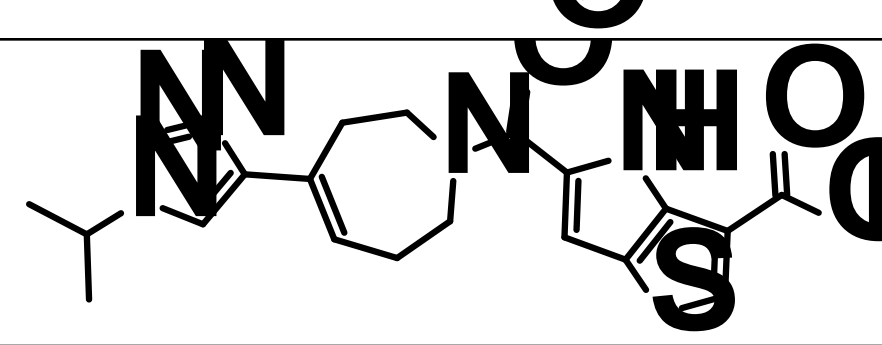	Z9698696292
**26**	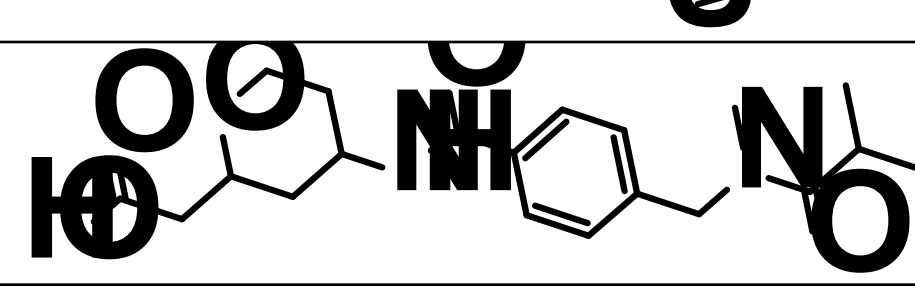	Z3510250611
**27**	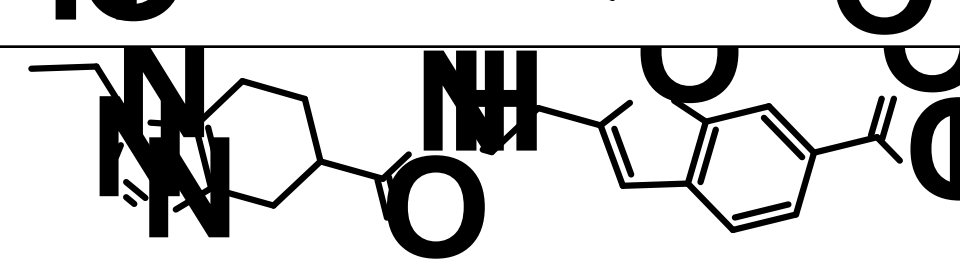	Z9698696354
**28**	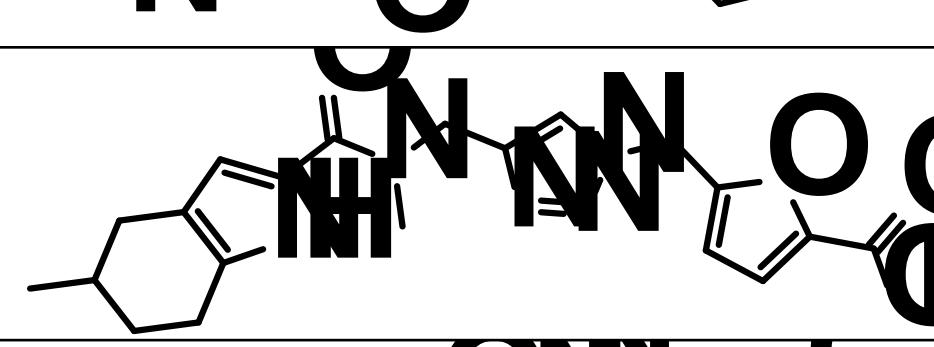	Z9698696337
**29**	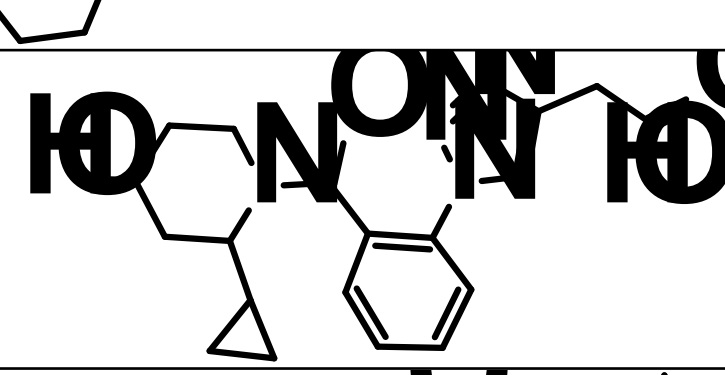	Z9698696303
**30**	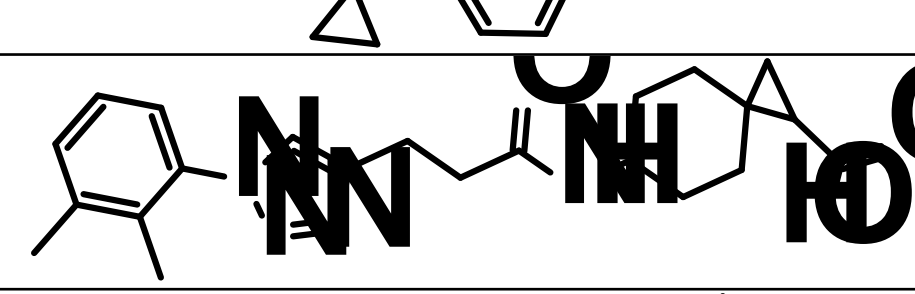	Z9698696268
**31**	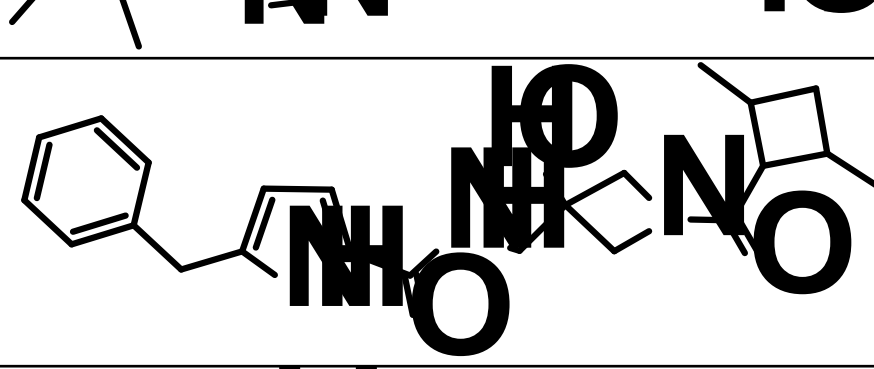	Z9698696391
**32**	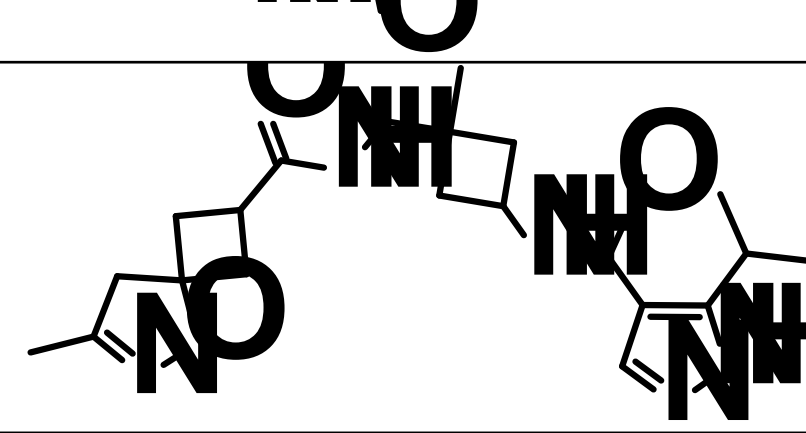	Z9698696302
**33**	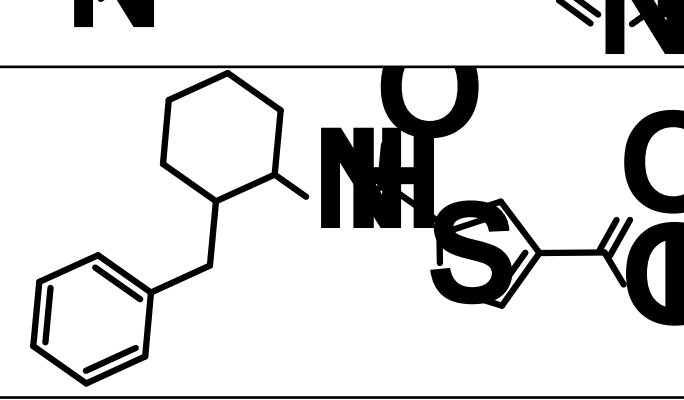	Z9698696284
**34**	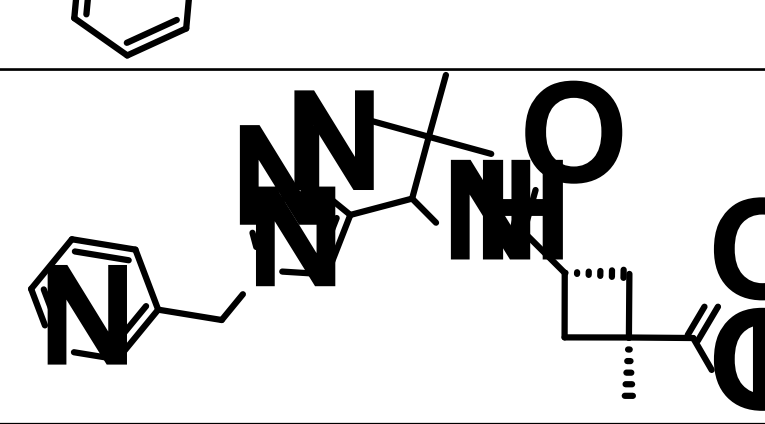	Z9698696404
**35**	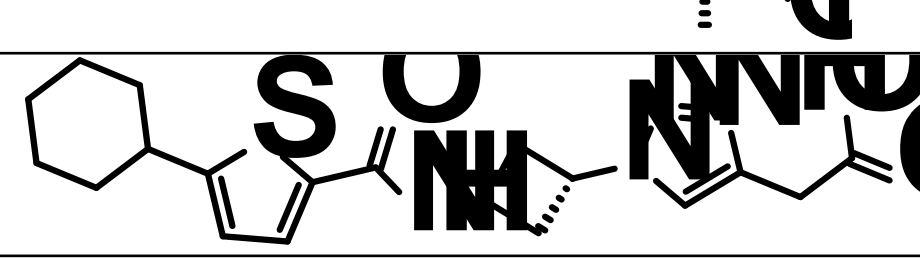	Z9698696365

**36**	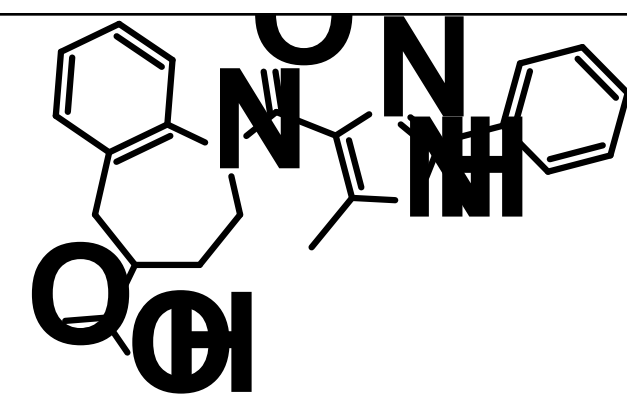	Z9698696281
**37**	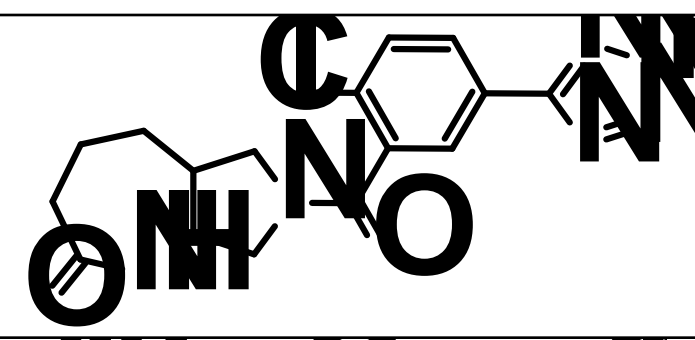	Z9698696301
**38**	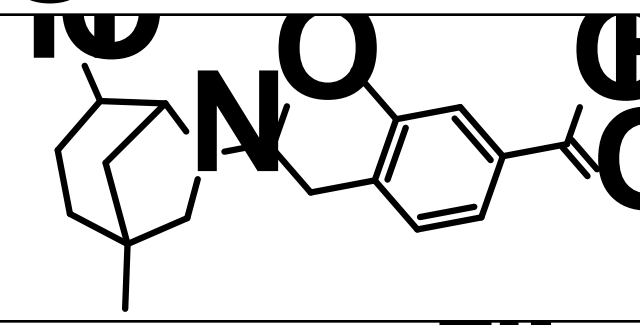	Z3509247428
**39**	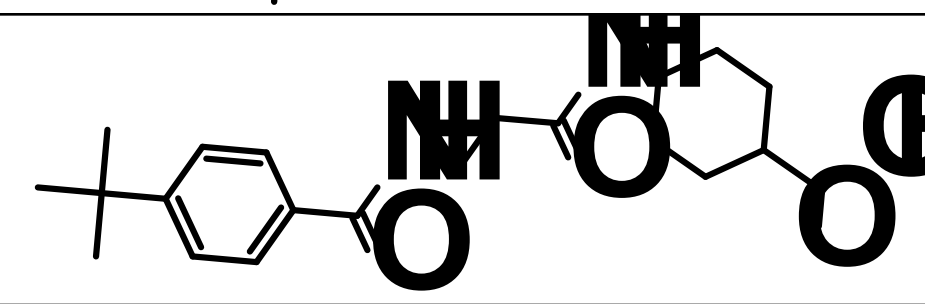	Z9698696362
**40**	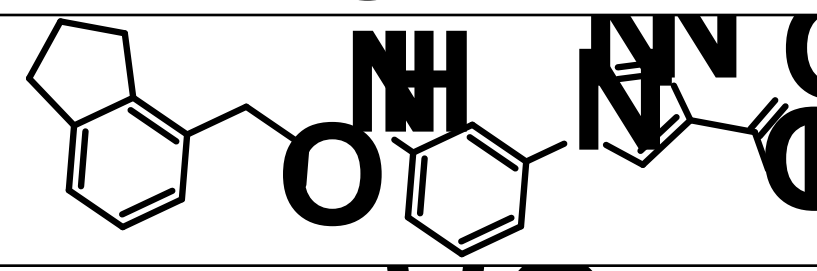	Z9698696429
**41**	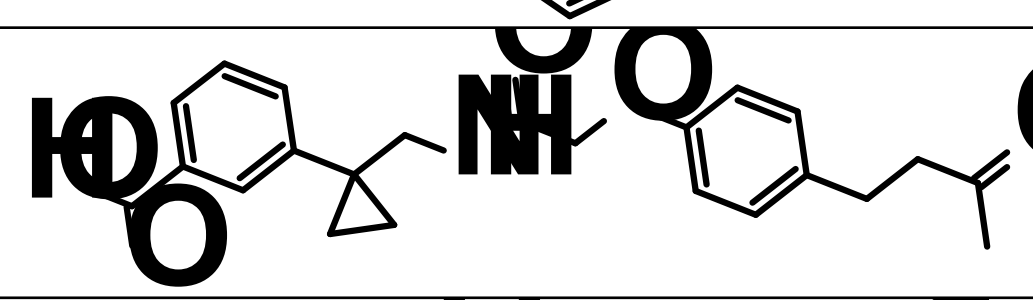	Z4577965976
**42**	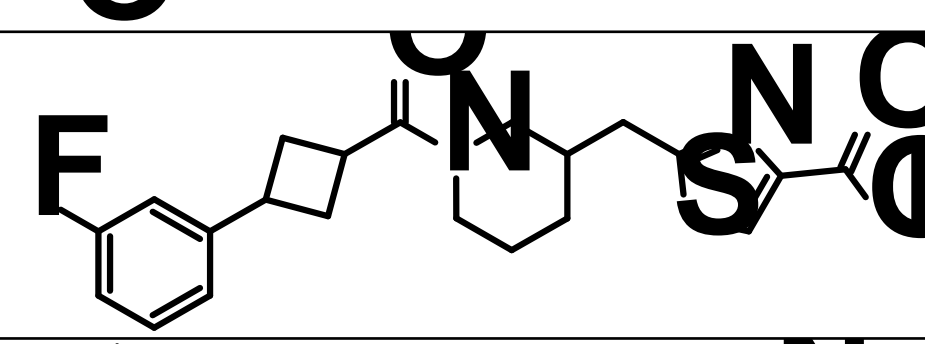	Z9698696359
**43**	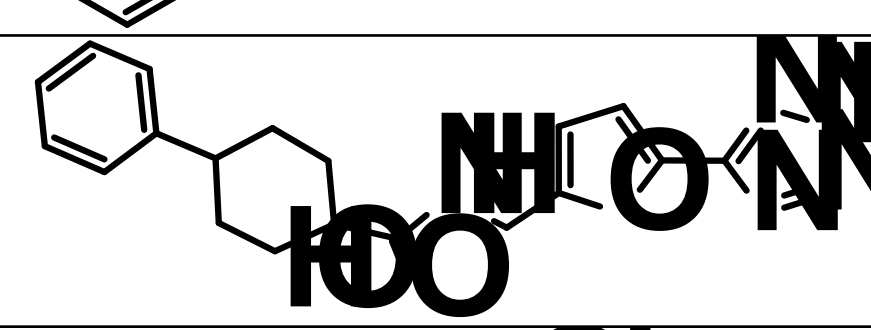	Z9698696378
**44**	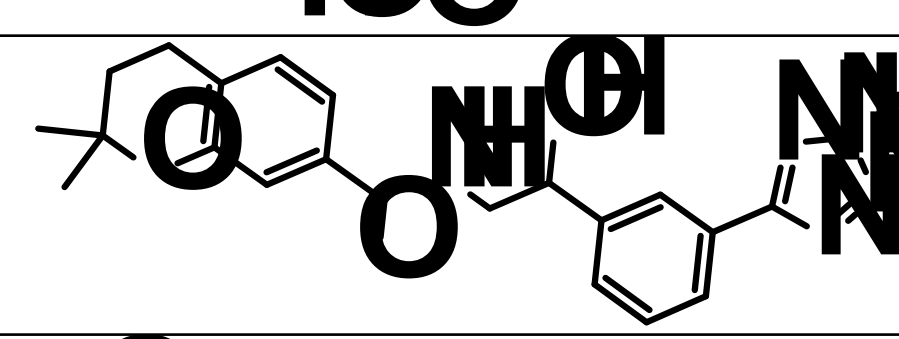	Z9698696363
**45**	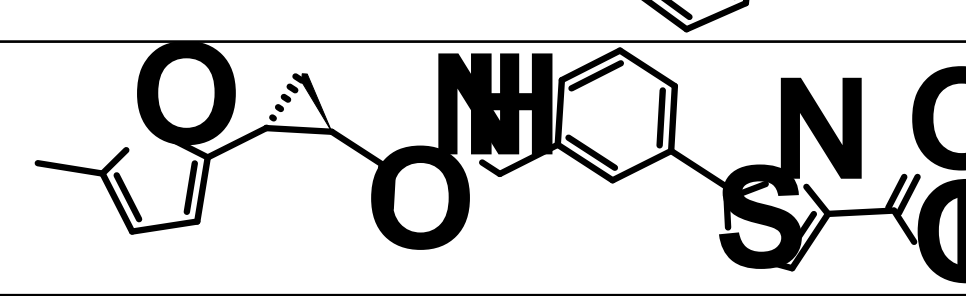	Z9698696348
**46**	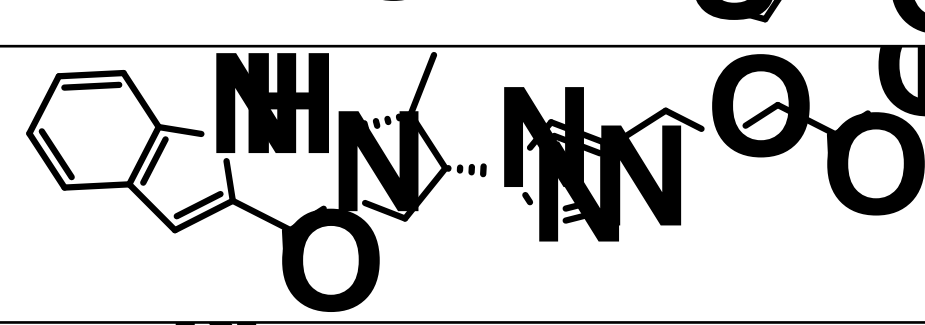	Z9698696416
**47**	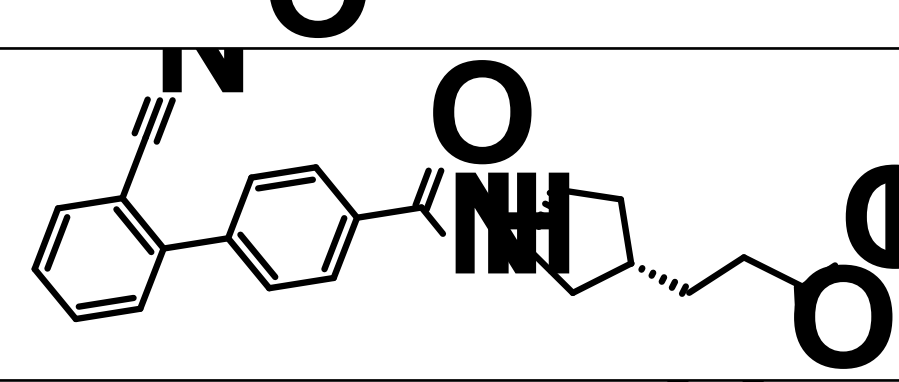	Z9698696396
**48**	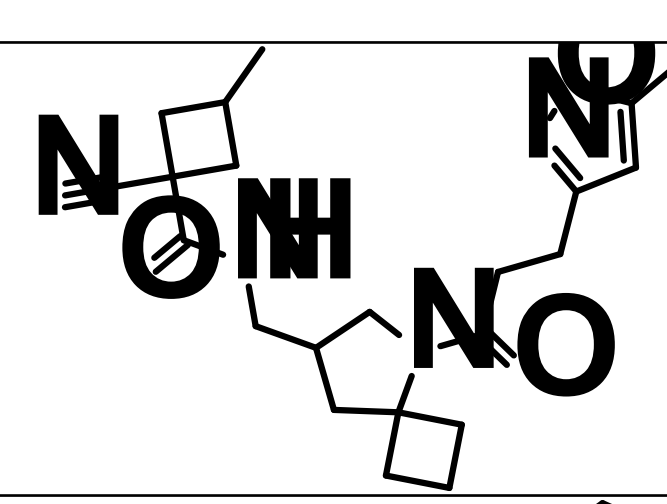	Z9698696418
**49**	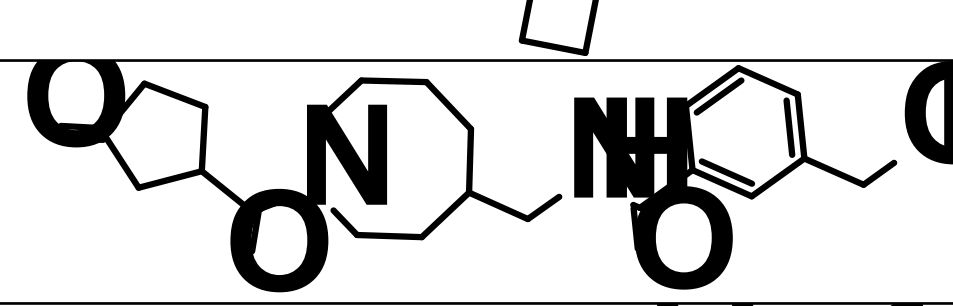	Z9698696410
**50**	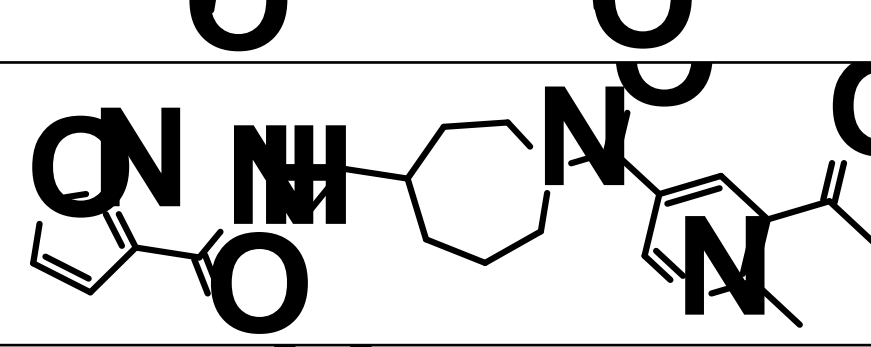	Z9698696427
**51**	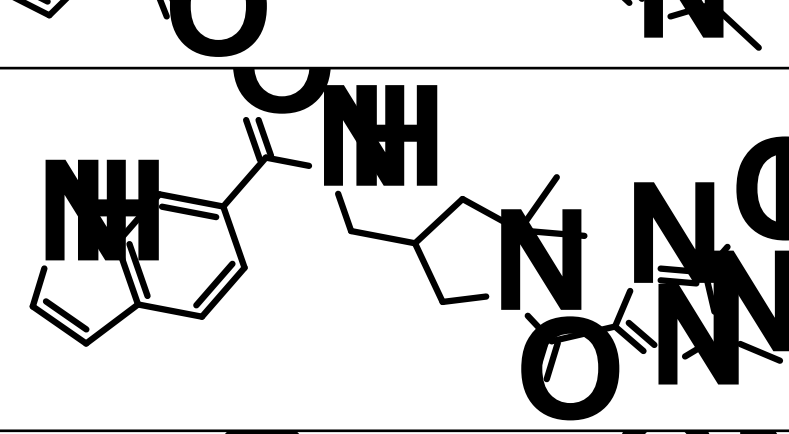	Z9698696405
**52**	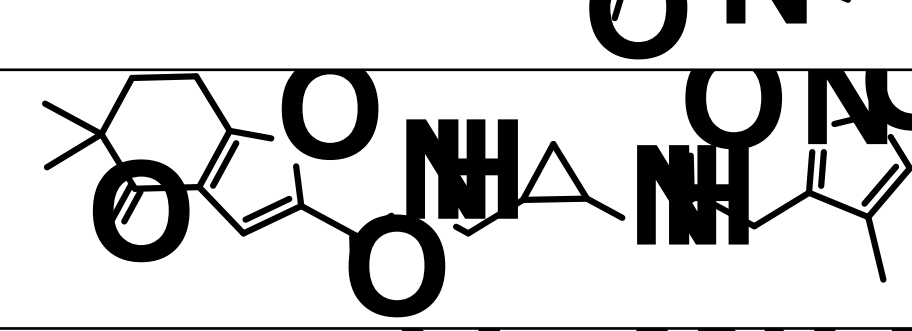	Z9698696398
**53**	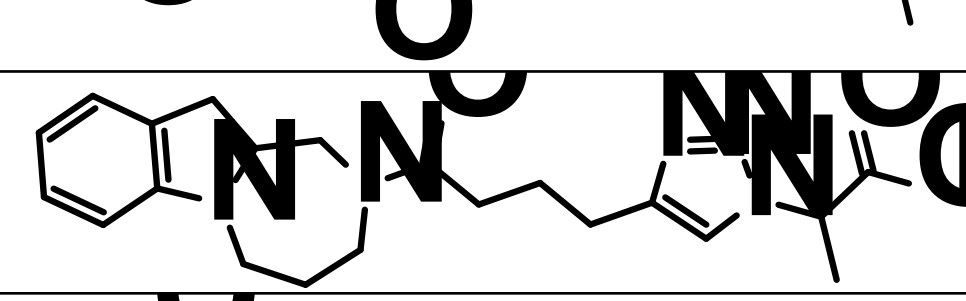	Z9698696272
**54**	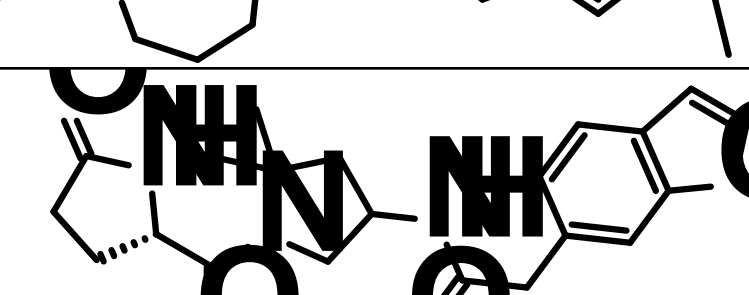	Z9698696413

**55**	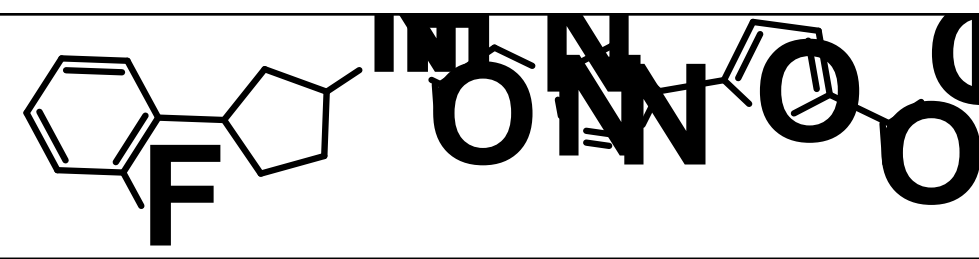	Z9698696345
**56**	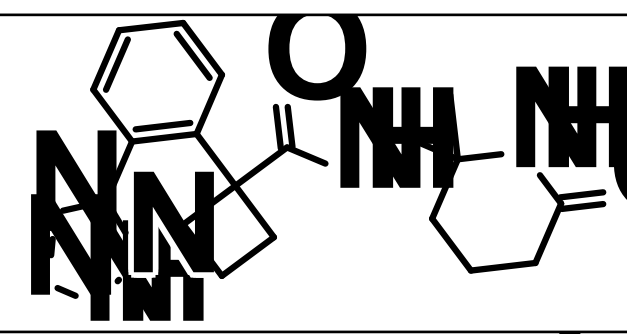	Z9698696290
**57**	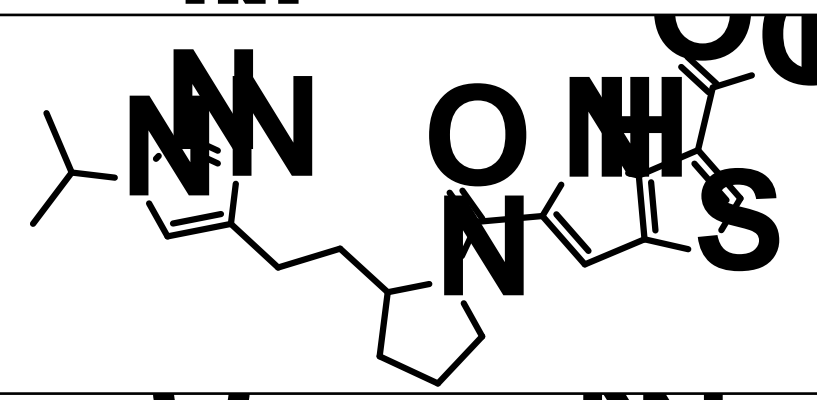	Z9698696289
**58**	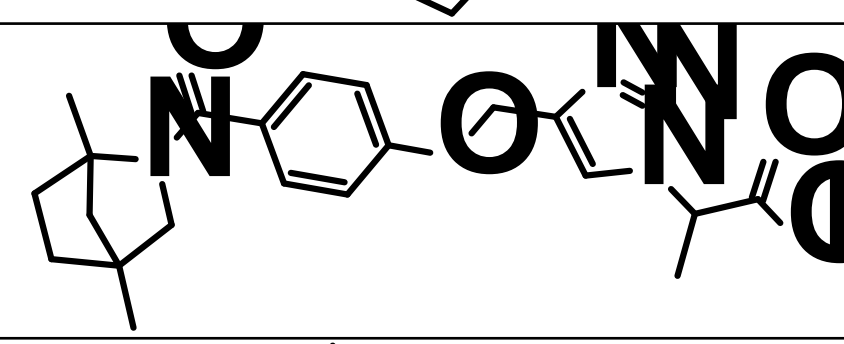	Z9698696388
**59**	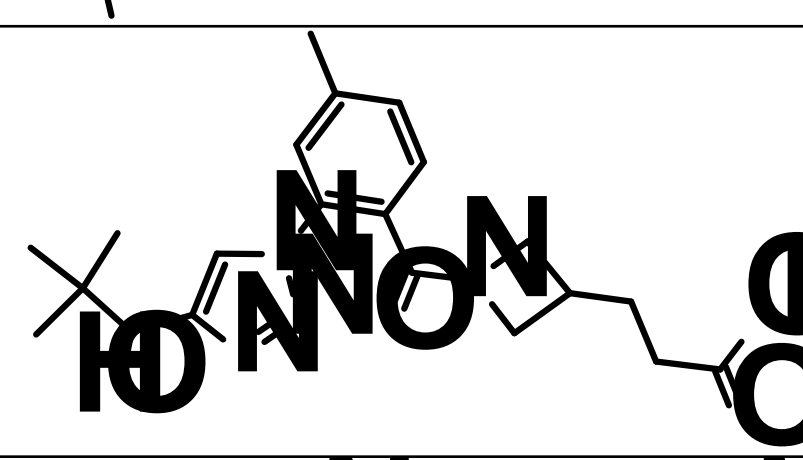	Z9698696293
**60**	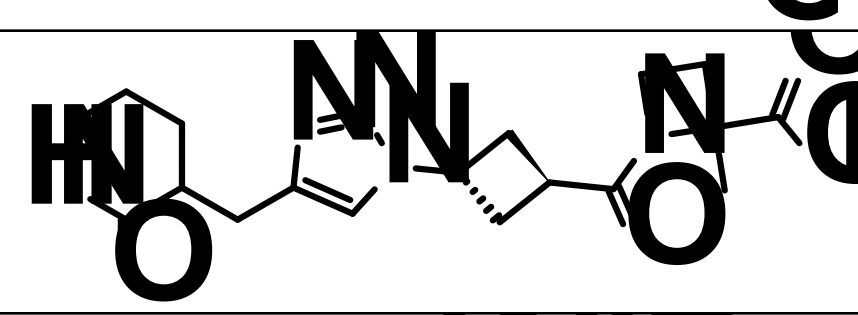	Z9698696264
**61**	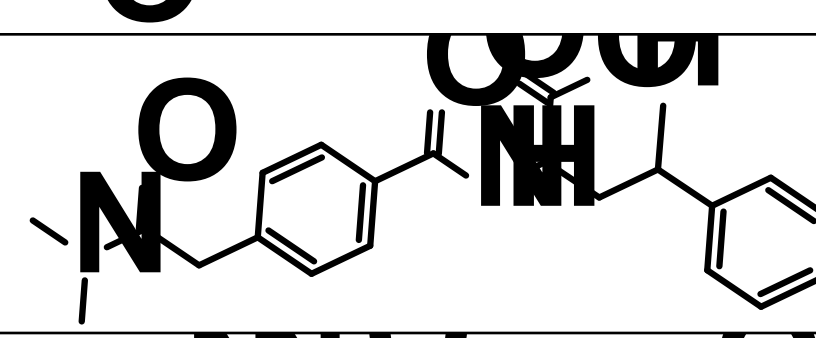	Z4590802640
**62**	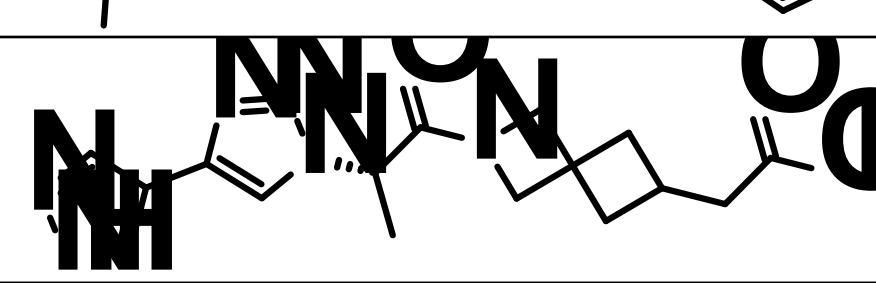	Z9698696274
**63**	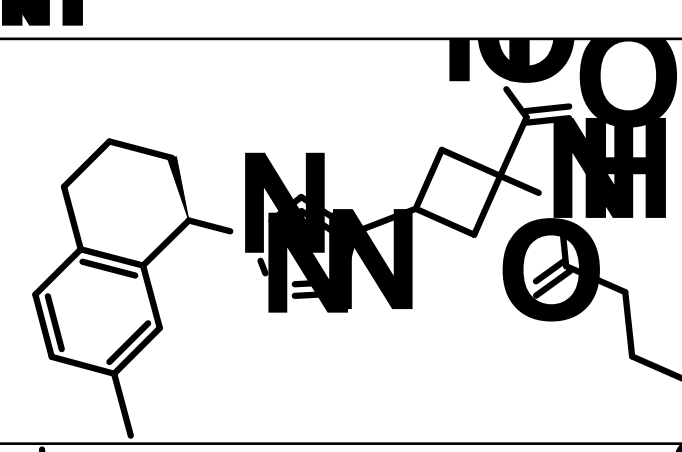	Z9698696383
**64**	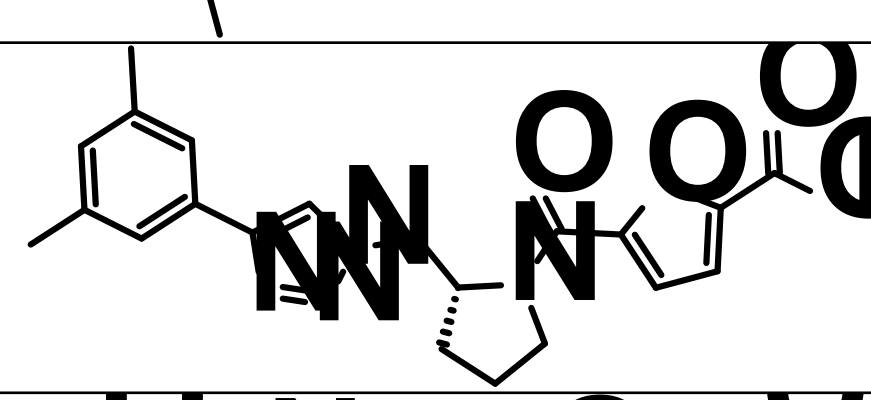	Z9698696349
**65**	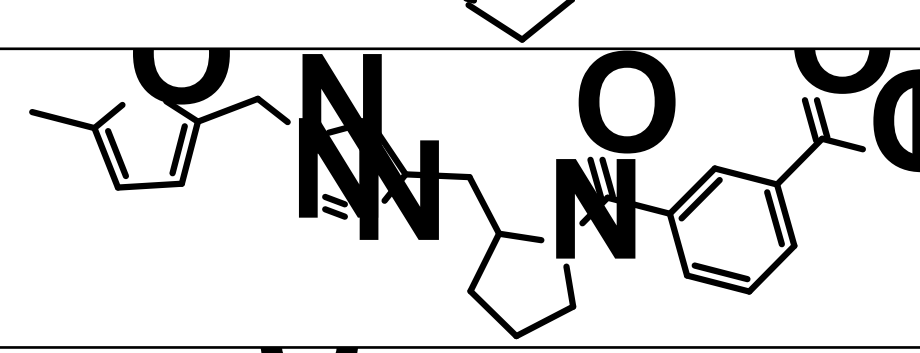	Z9698696417
**66**	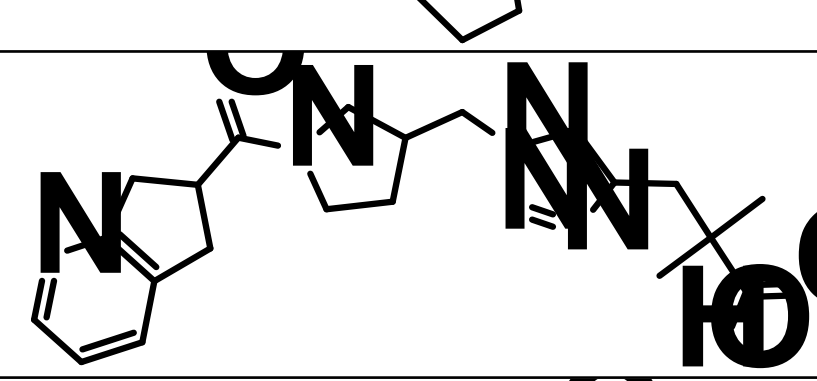	Z9698696360
**67**	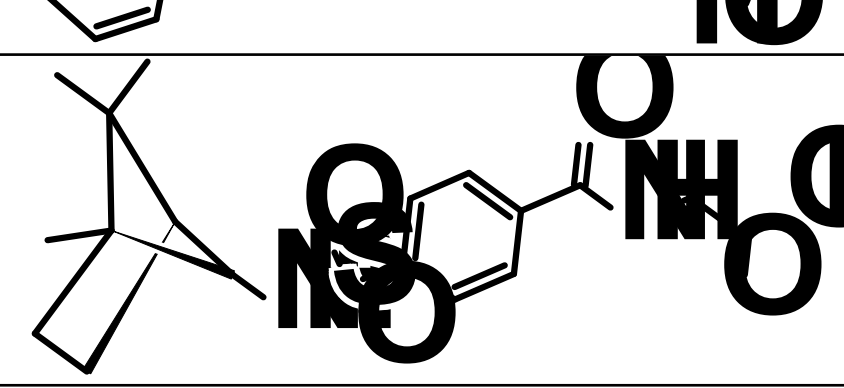	Z9698696299
**68**	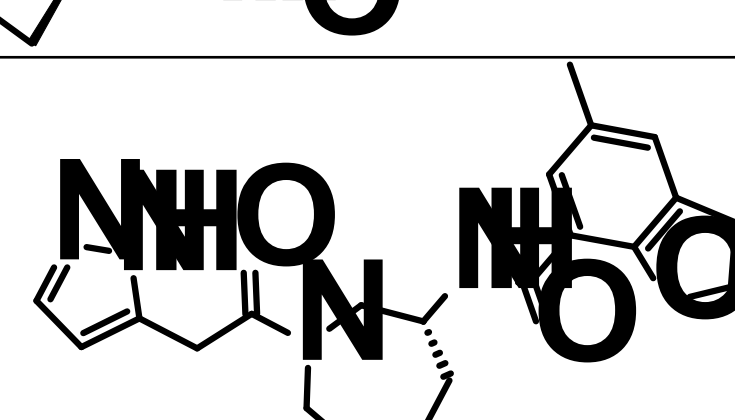	Z9698696403
**69**	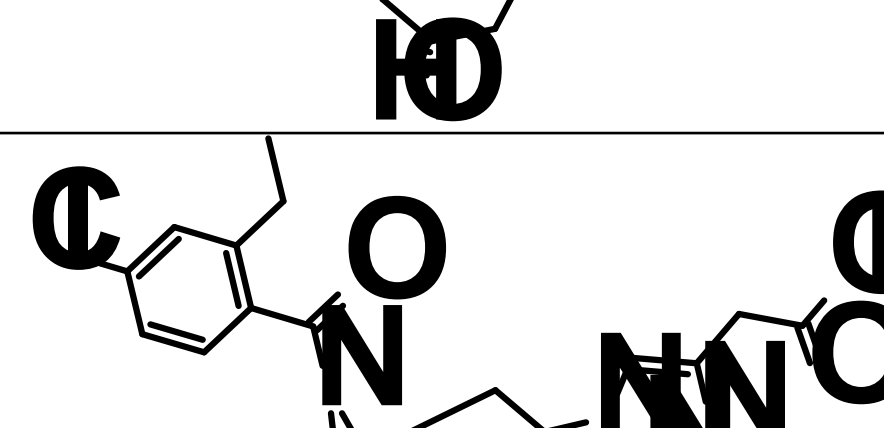	Z9698696275
**70**	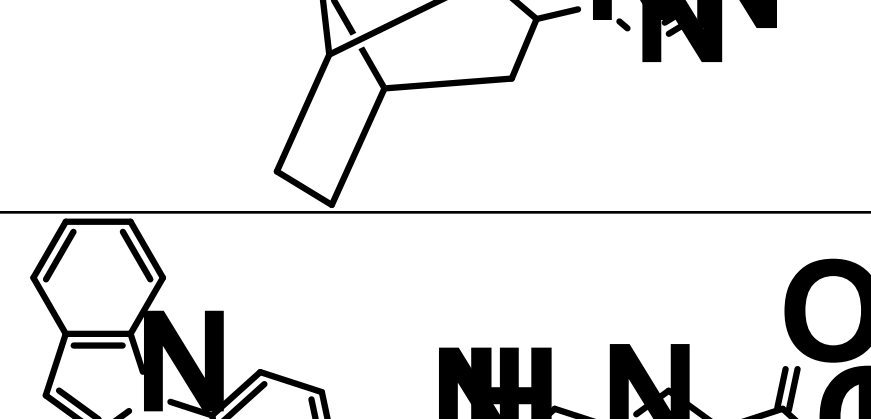	Z9698696361

**71**	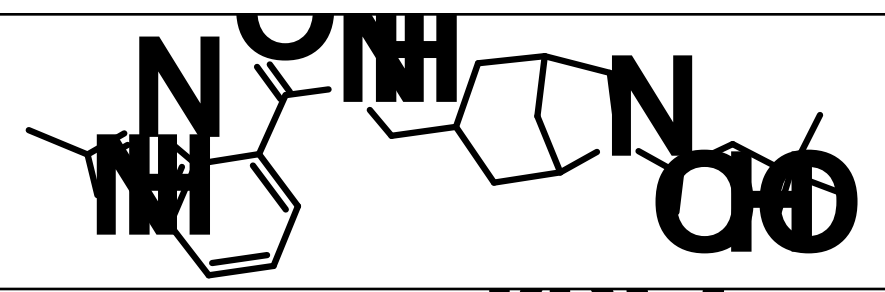	Z9698696423
**72**	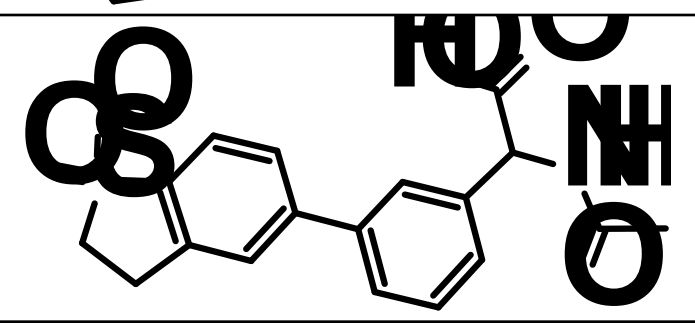	Z9698696265
**73**	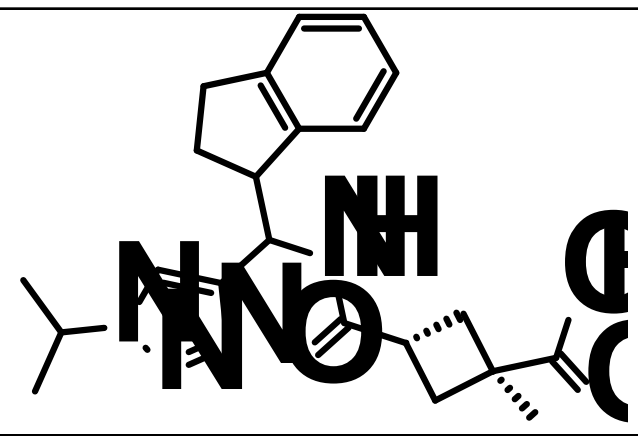	Z9698696279
**74**	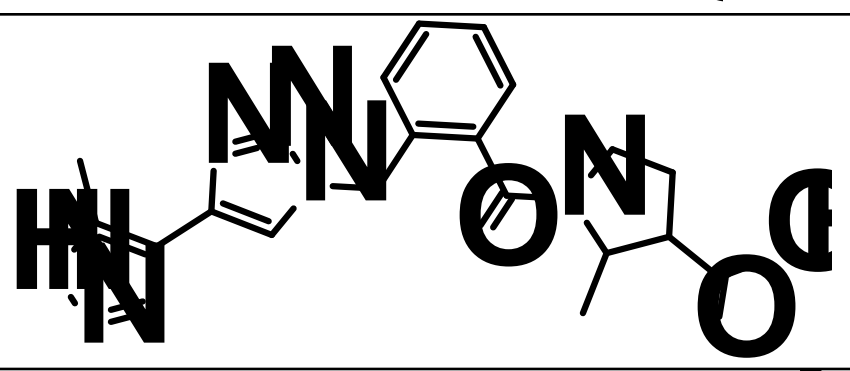	Z9698696277
**75**	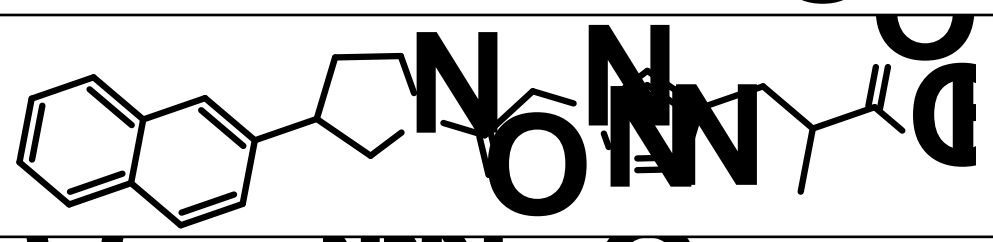	Z9698696353
**76**	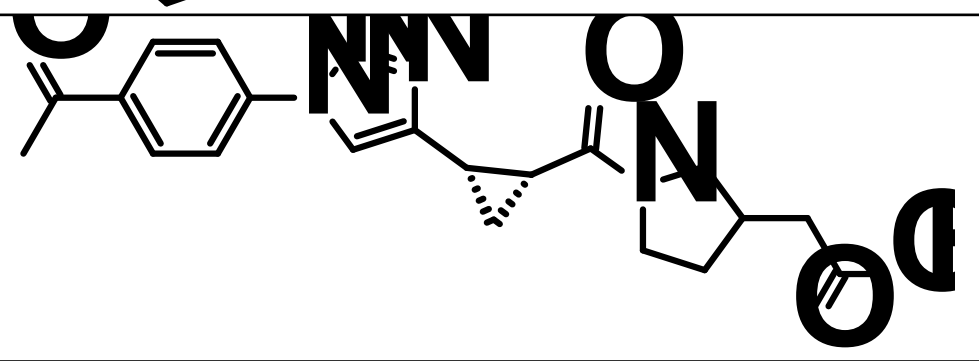	Z9698696386
**77**	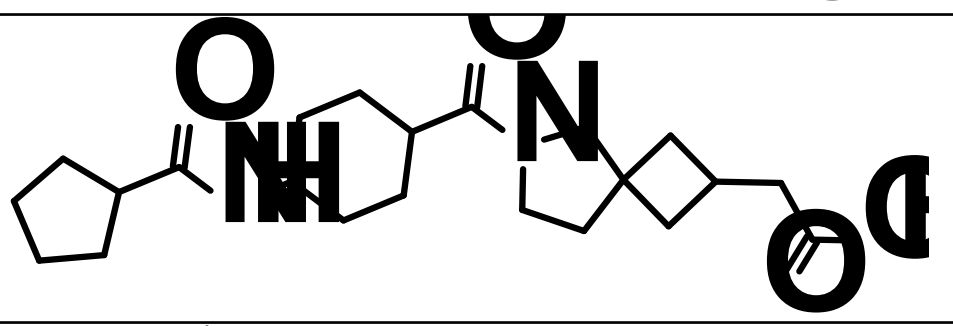	Z9698696273
**78**	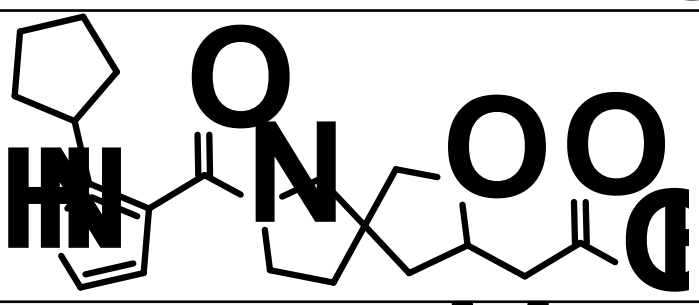	Z5074828592
**79**	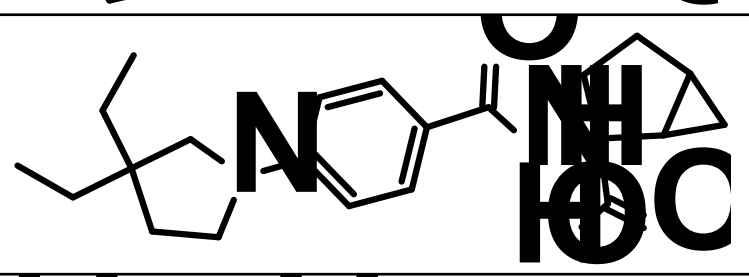	Z9698696306
**80**	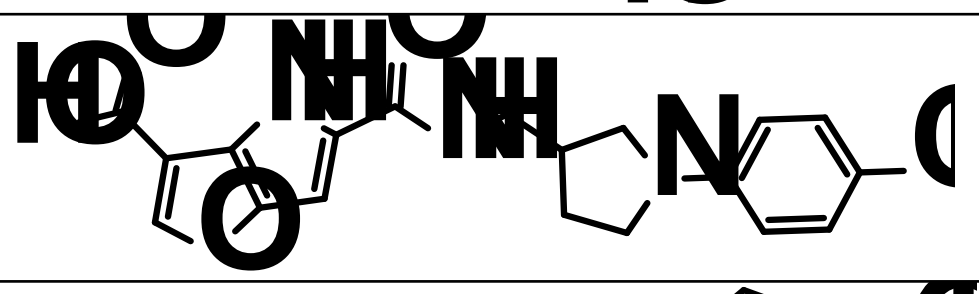	Z9698696305
**81**	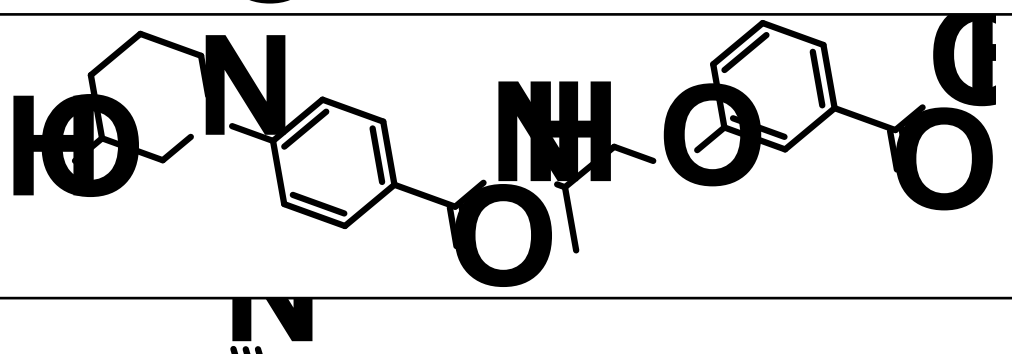	Z6645588208
**82**	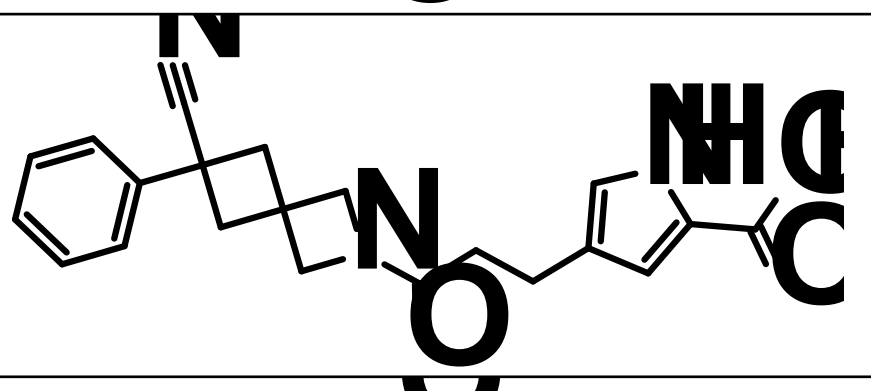	Z6654658182
**83**	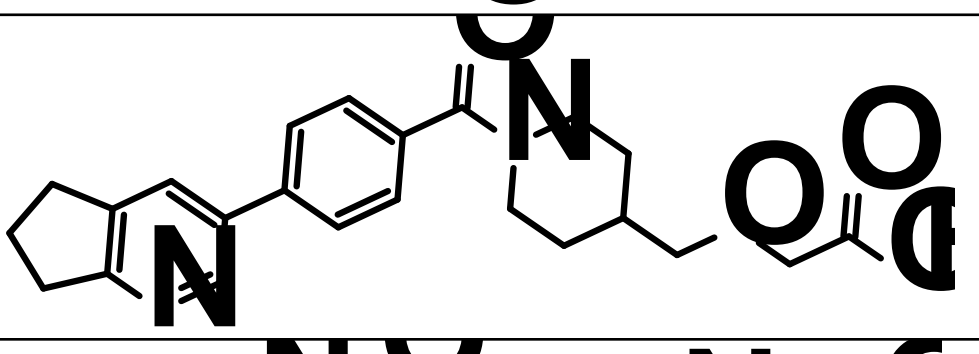	Z6704781071
**84**	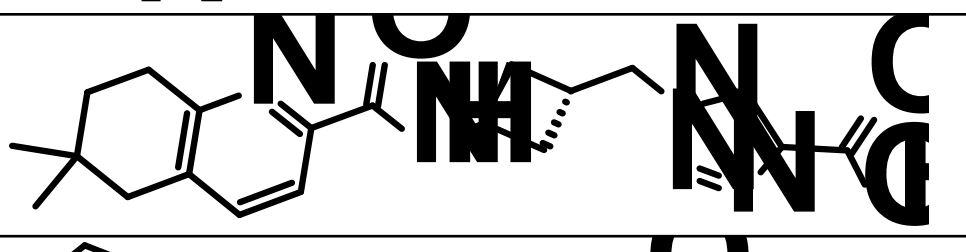	Z9698696387
**85**	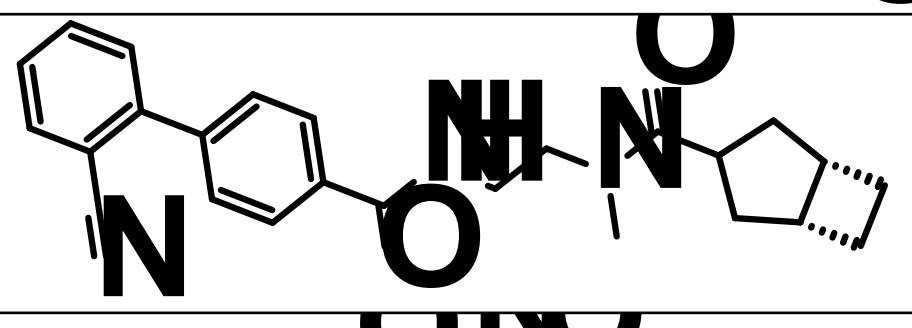	Z9698696392
**86**	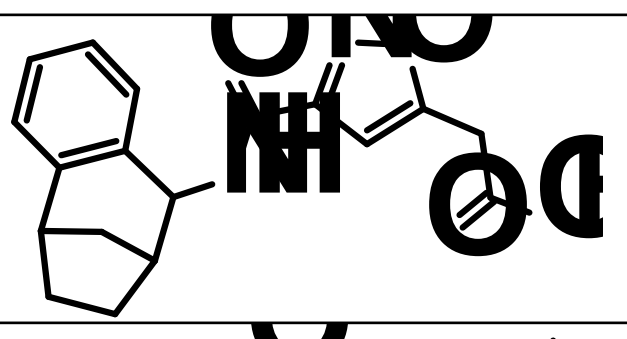	Z5643051772
**87**	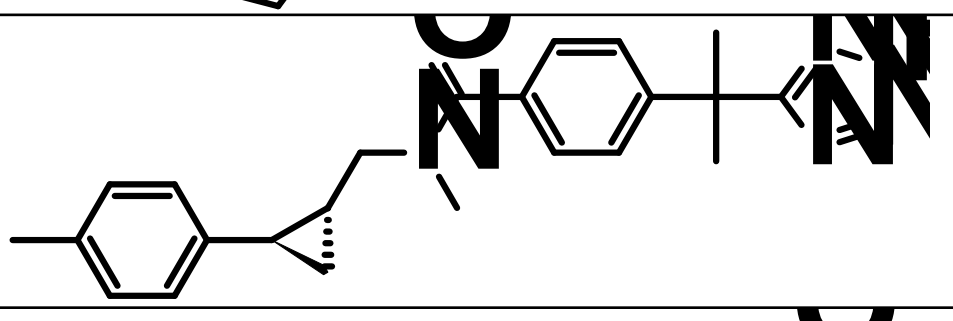	Z9698696351
**88**	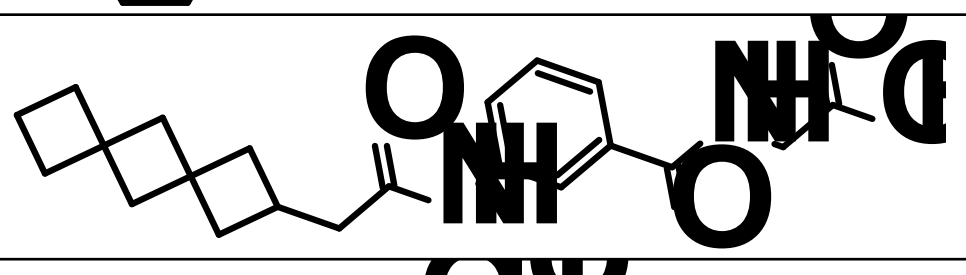	Z9698696267
**89**	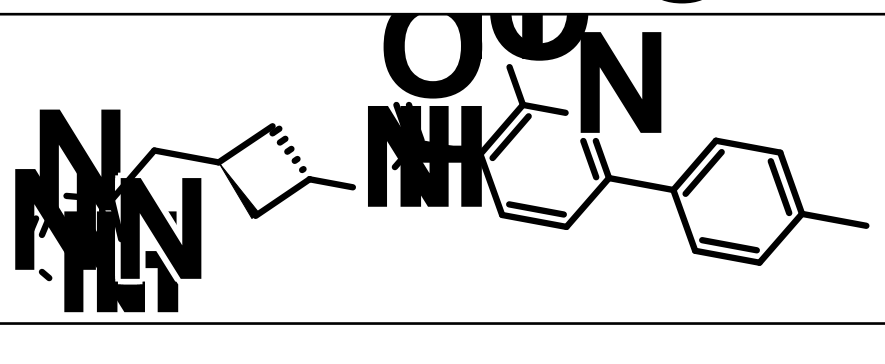	Z9698696346

**90**	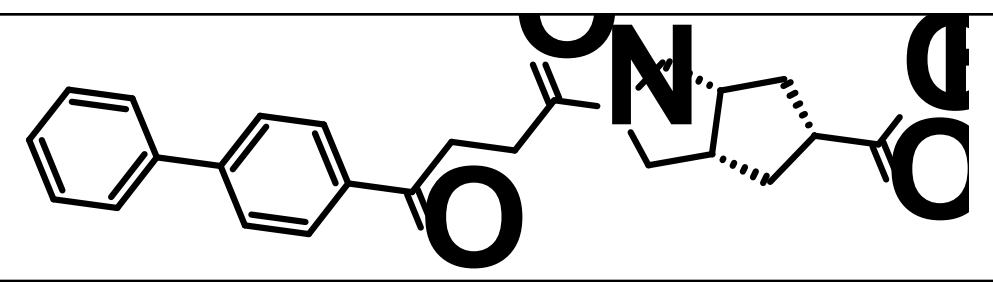	Z6671929133
**91**	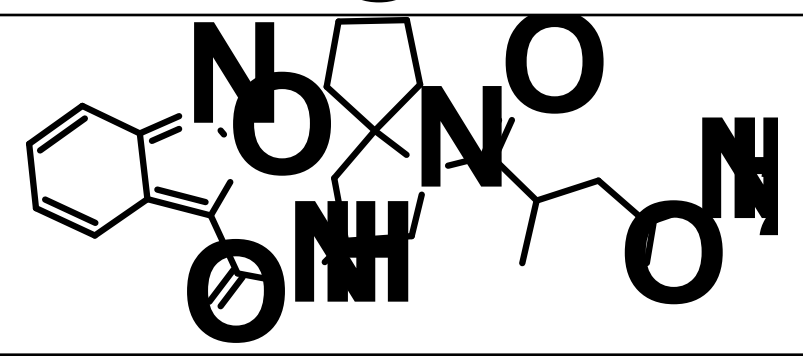	Z9698696430
**92**	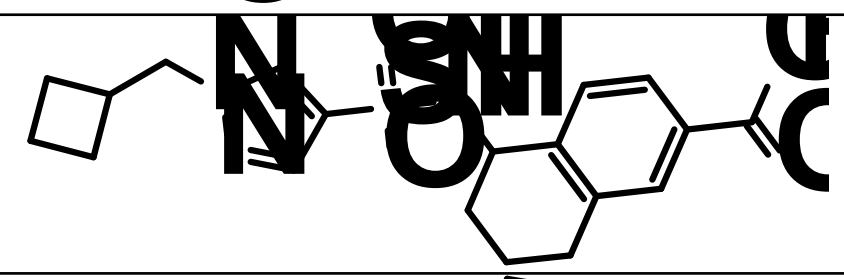	Z9698696285
**93**	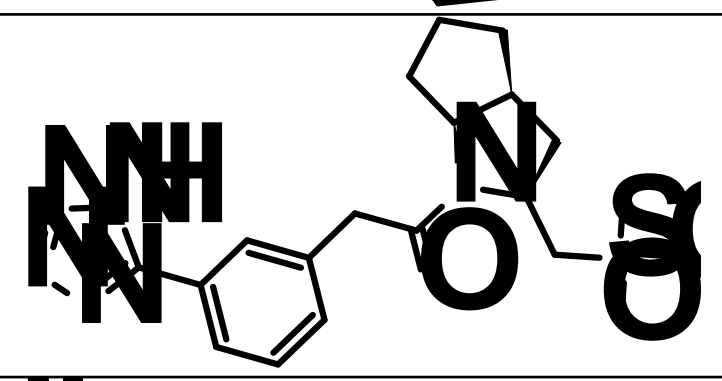	Z8173342514
**94**	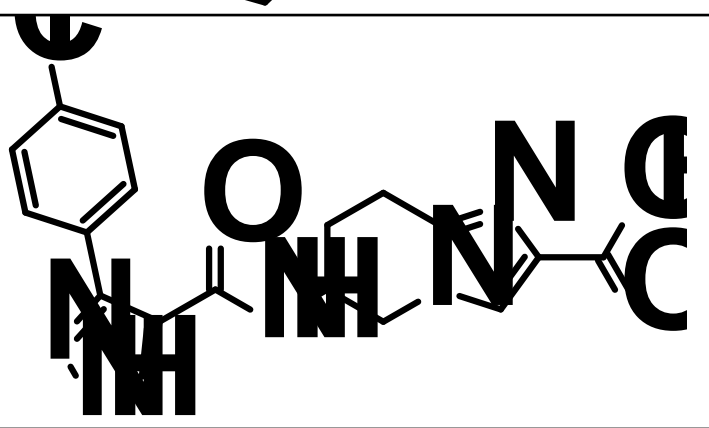	Z9916238374
**95**	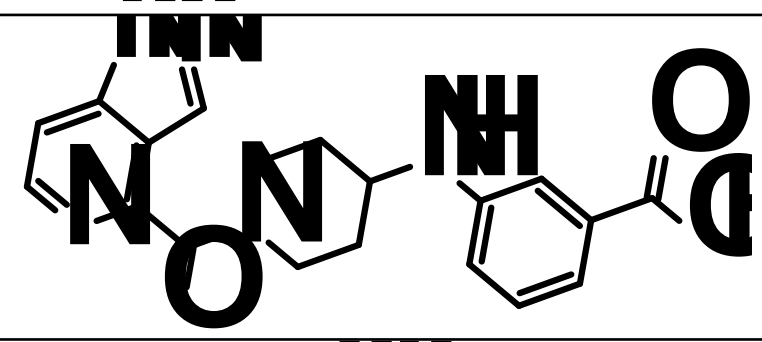	Z9916238372
**96**	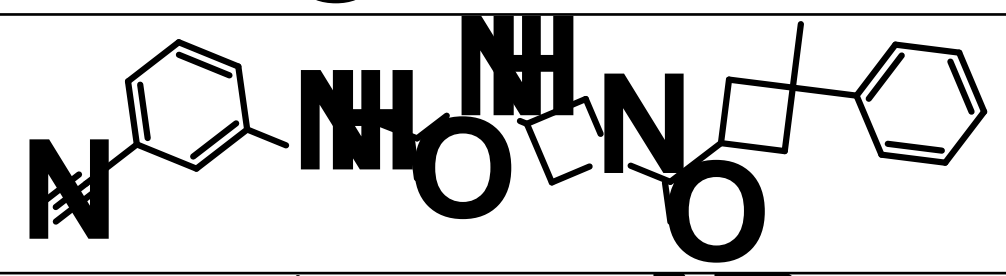	Z9698696397
**97**	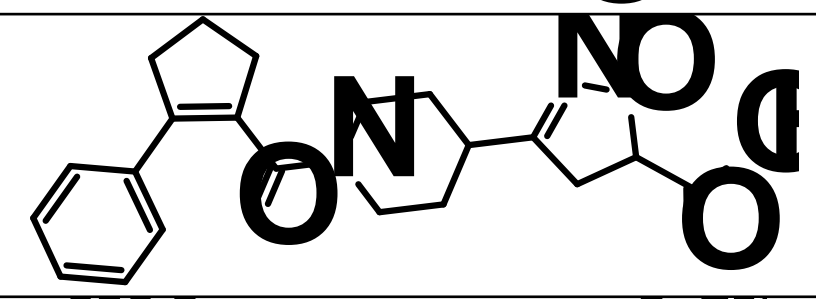	Z6683469397
**98**	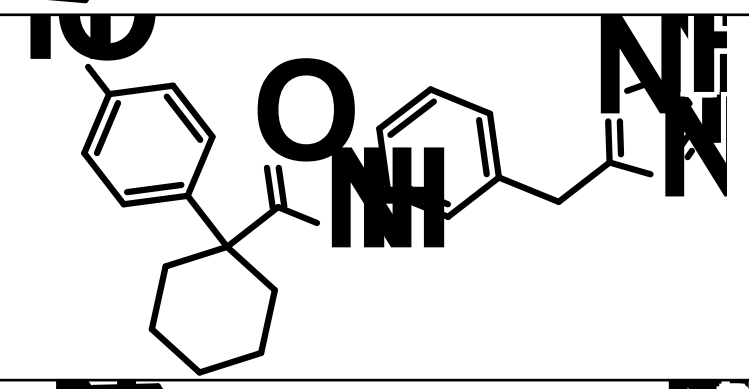	Z9698696407
**99**	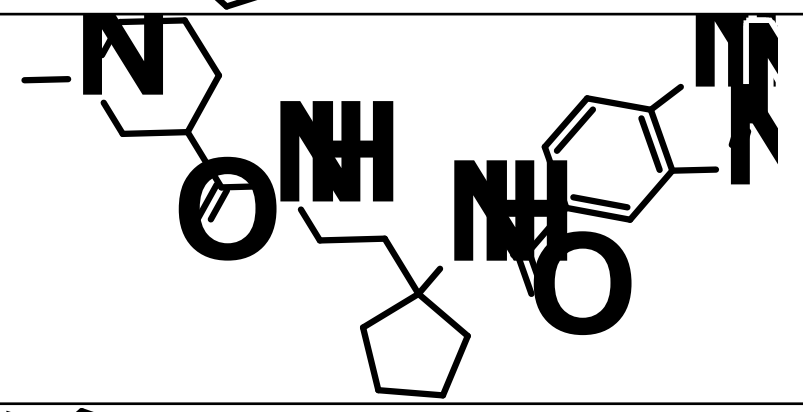	Z9698696411
**100**	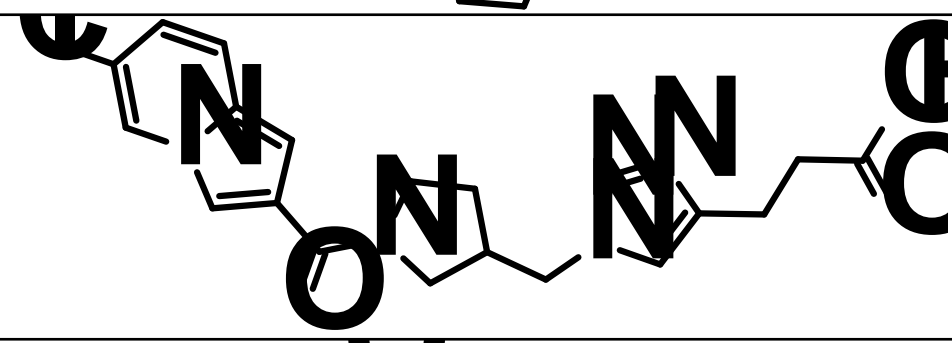	Z9698696355
**101**	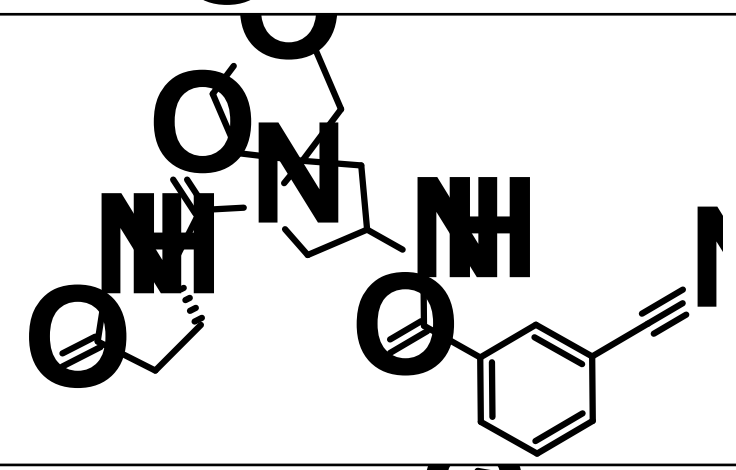	Z9698696415
**102**	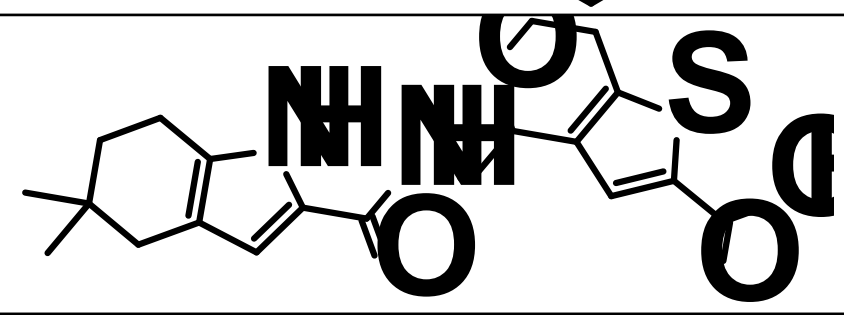	Z9698696374
**103**	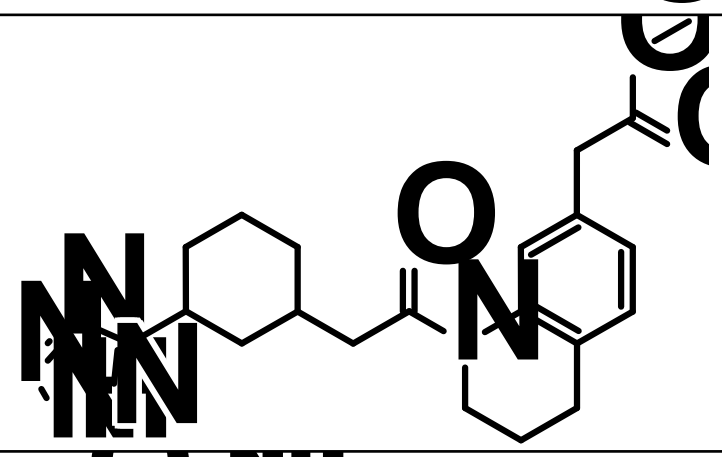	Z9698696425
**104**	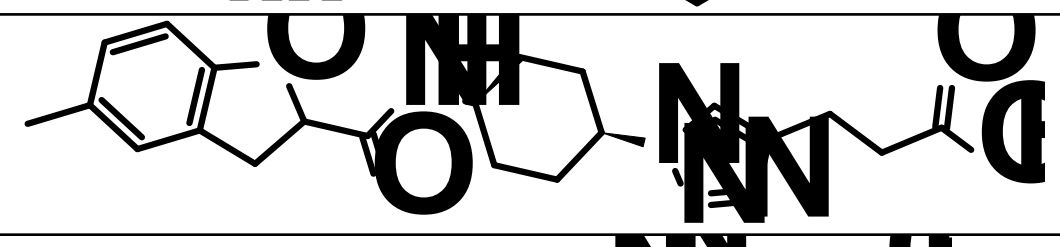	Z9698696402
**105**	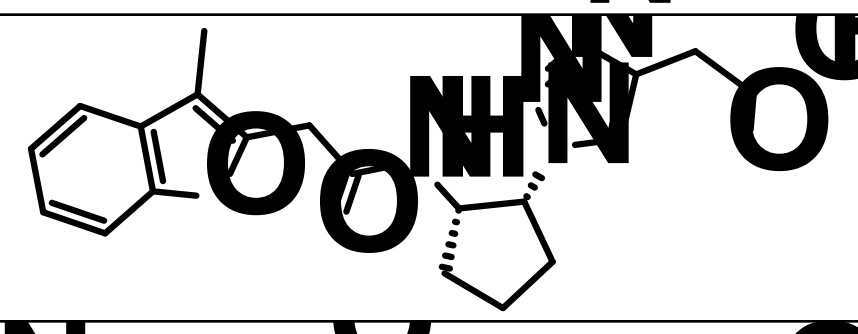	Z9698696426
**106**	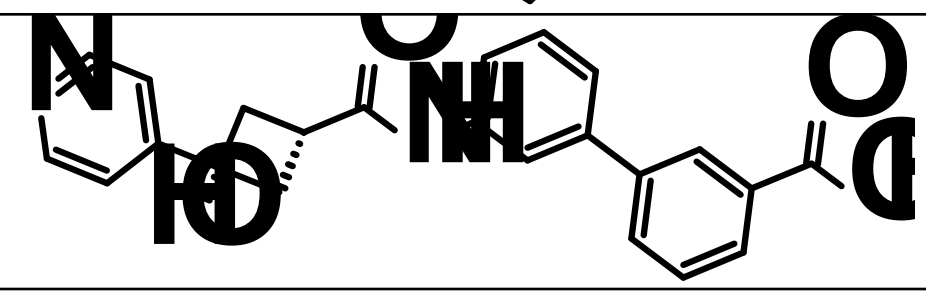	Z9698696352

**107**	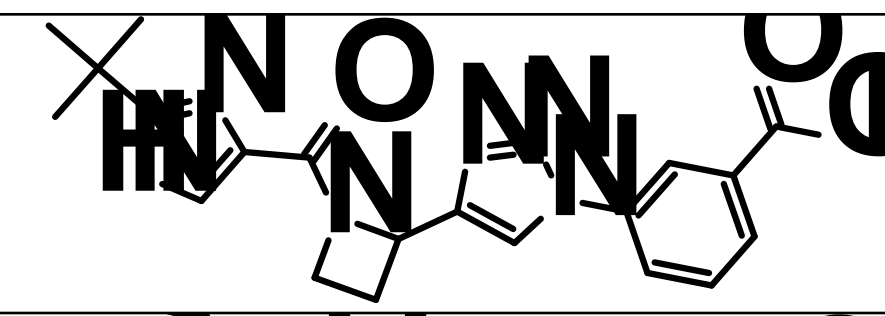	Z9698696347
**108**	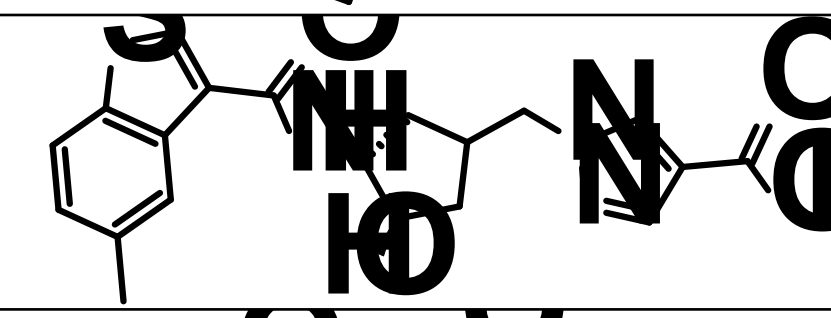	Z9698696283
**109**	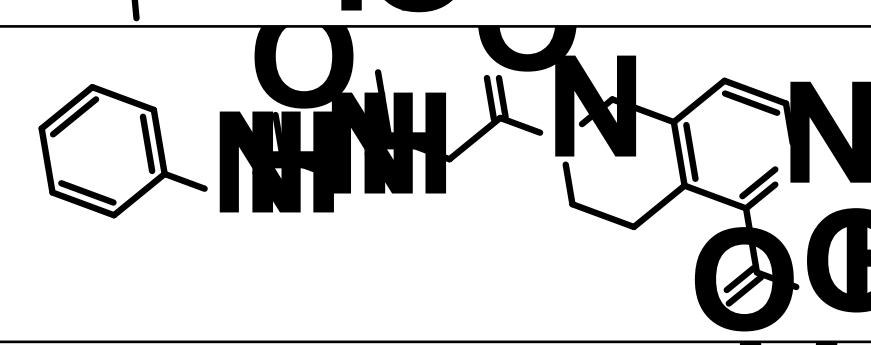	Z9698696276
**110**	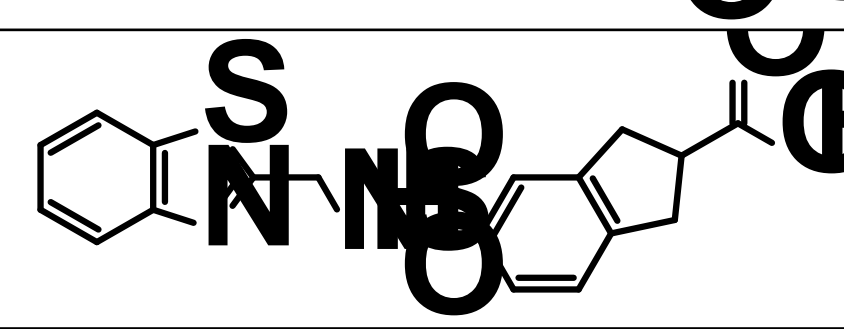	Z9698696286
**111**	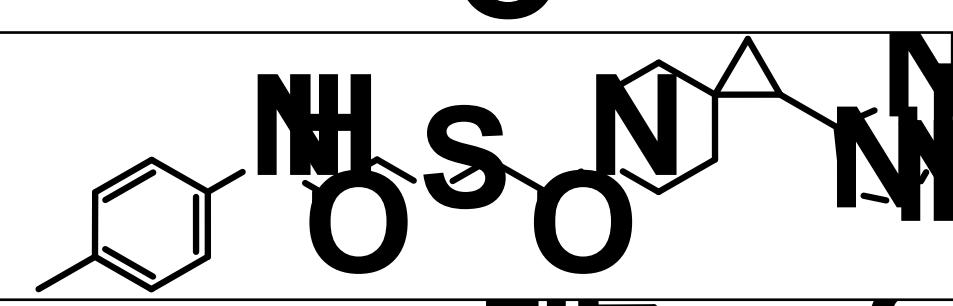	Z9698696357
**112**	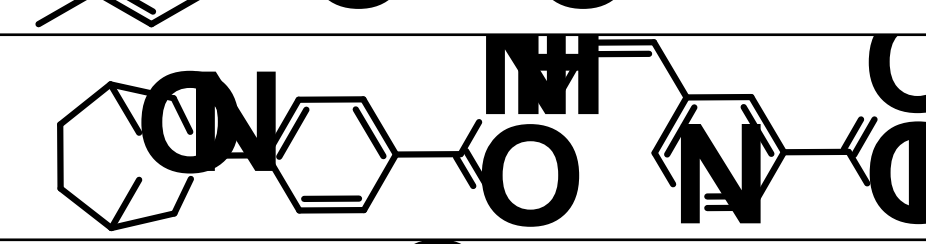	Z6661443394
**113**	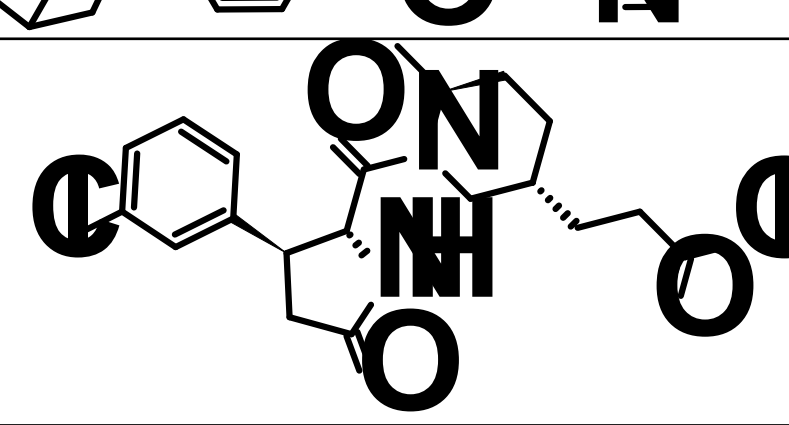	Z6706676830
**114**	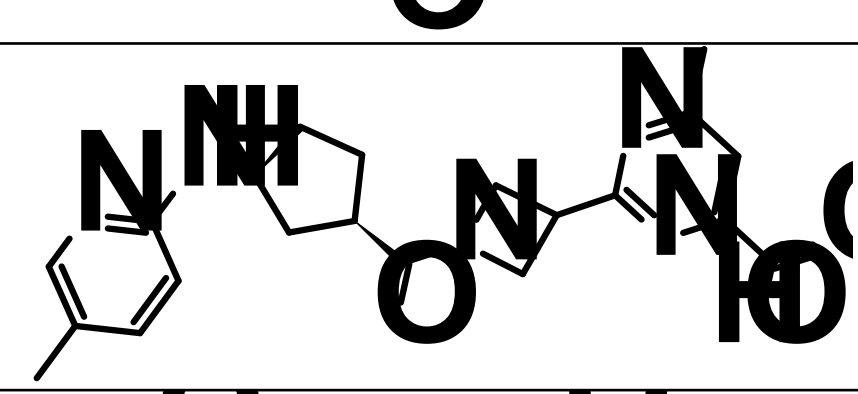	Z9916238373
**115**	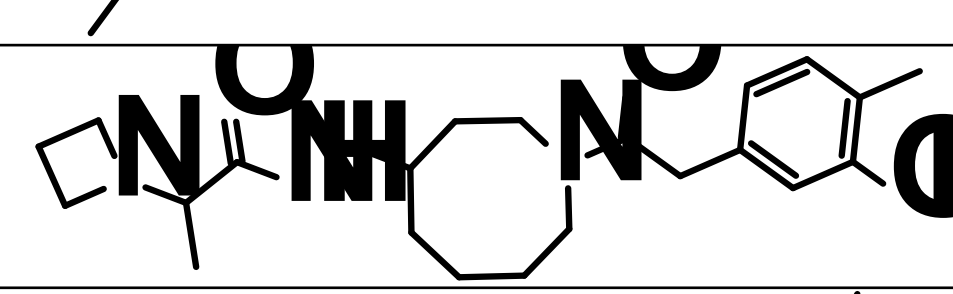	Z9918750513
**116**	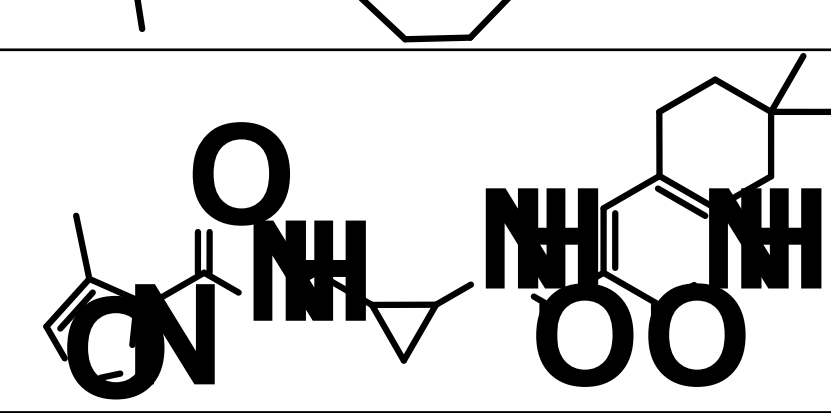	Z9698696393
**117**	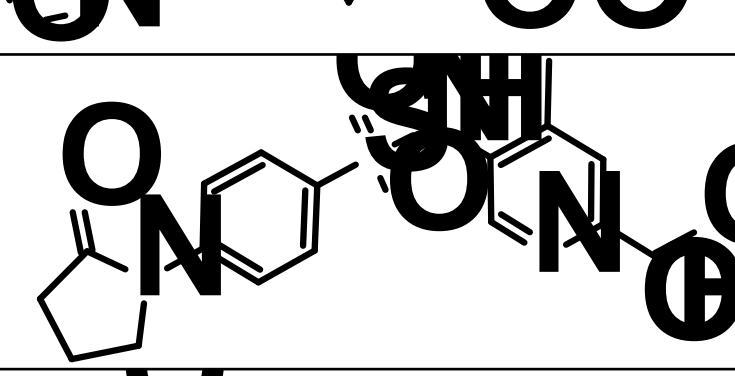	Z9698696307
**118**	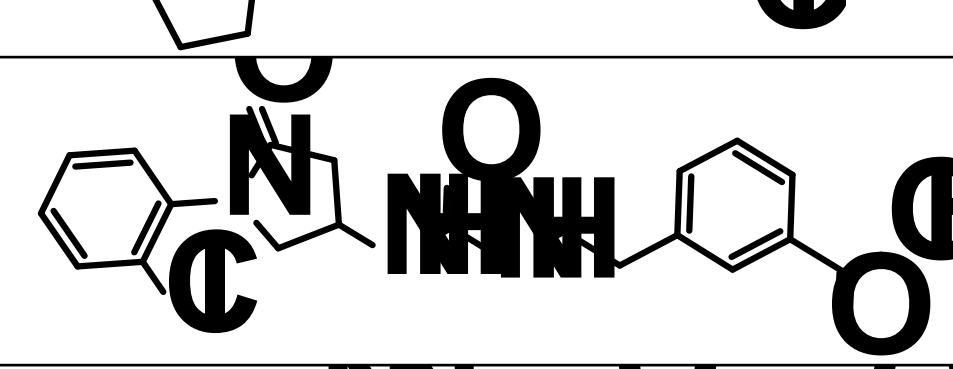	Z9698696373
**119**	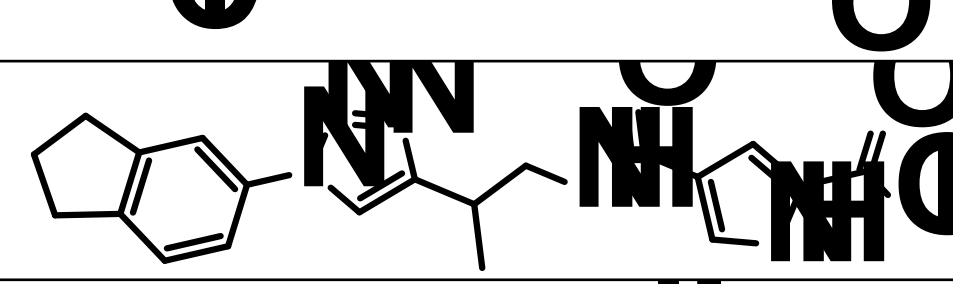	Z9698696364
**120**	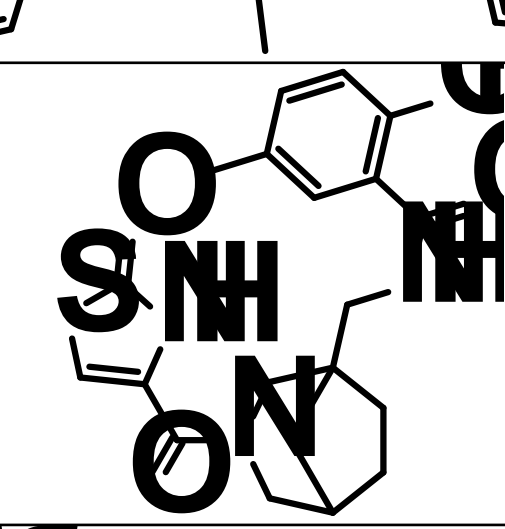	Z9698696421
**121**	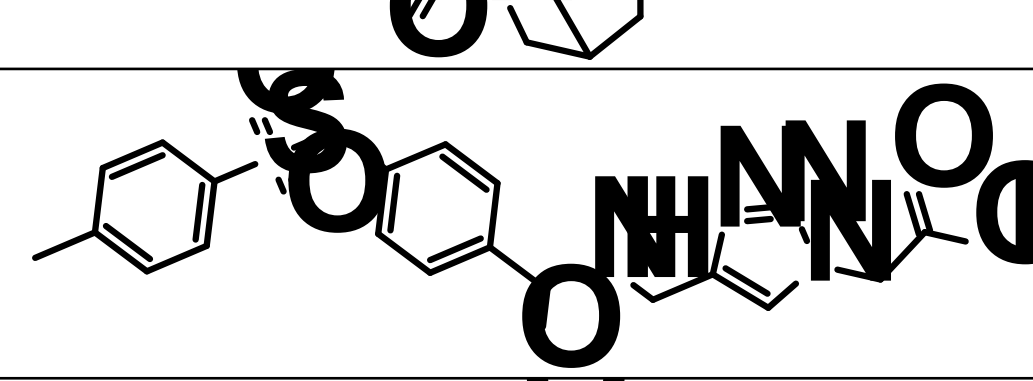	Z1991870008
**122**	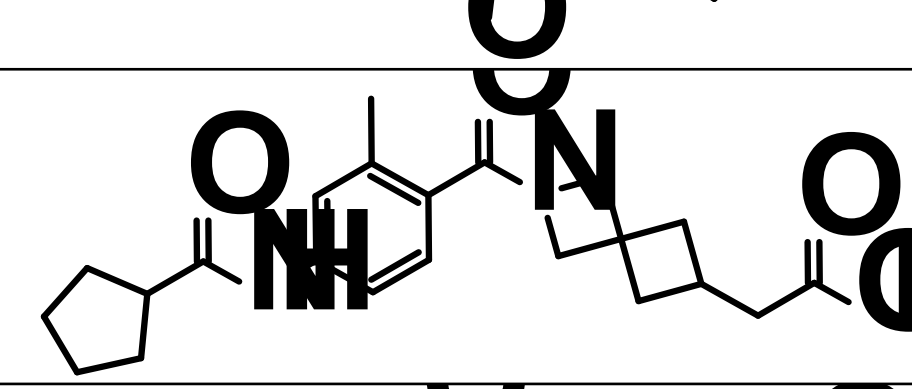	Z5273326489
**123**	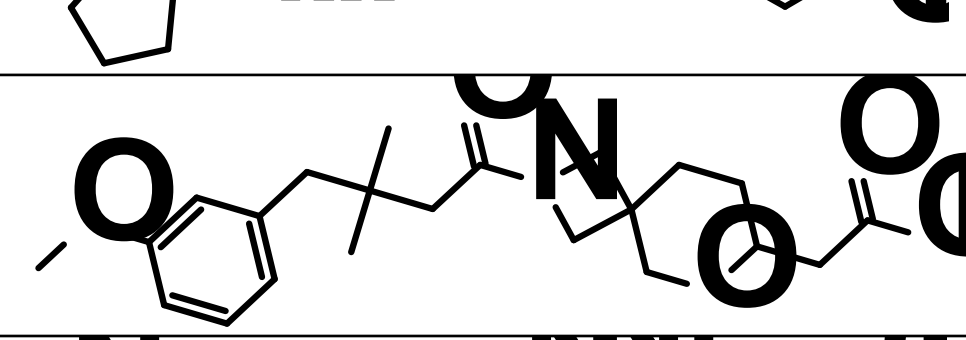	Z6682428204
**124**	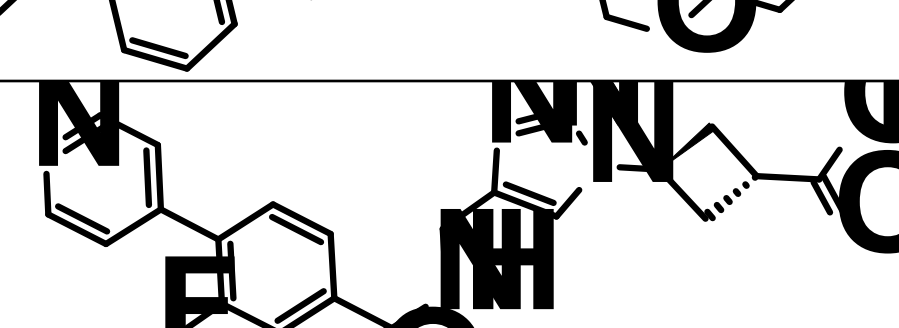	Z9698696333

**125**	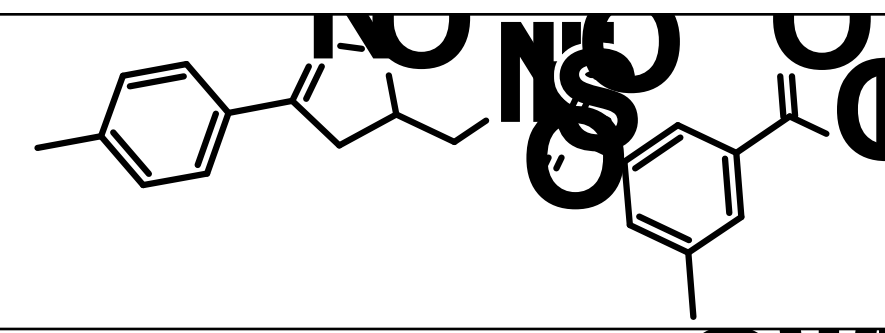	Z9698696338
**126**	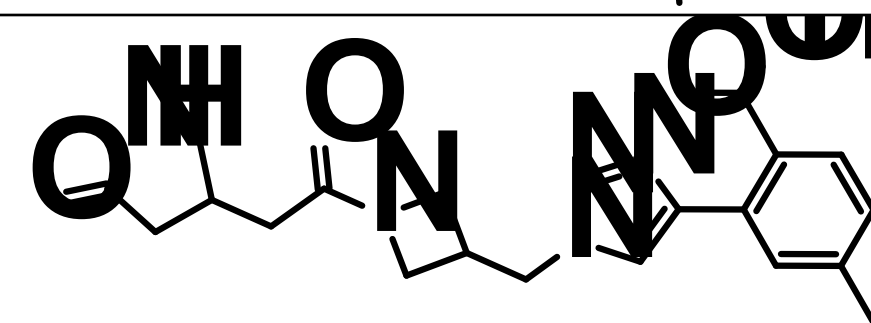	Z9698696255
**127**	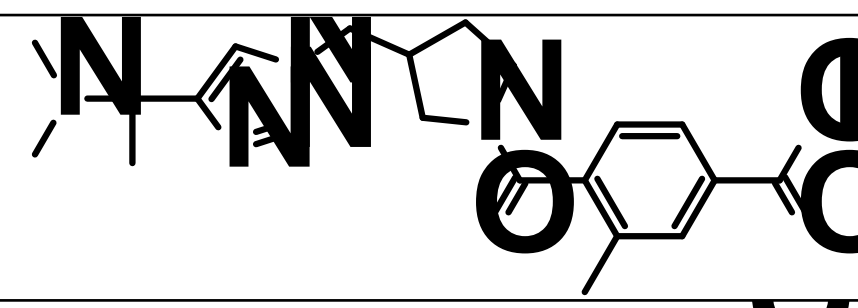	Z9698696258
**128**	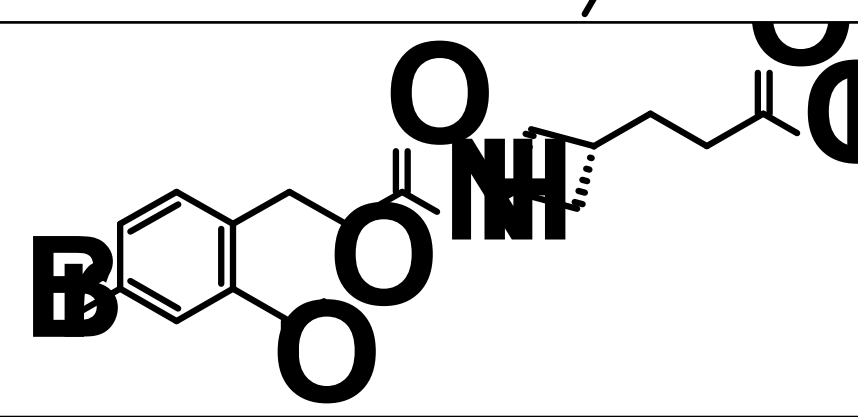	Z4577476016
**129**	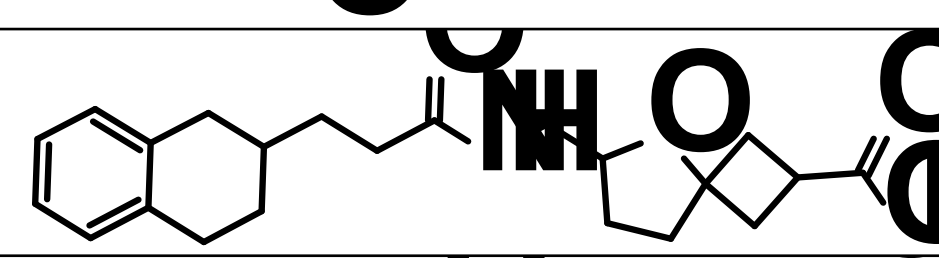	Z6096789177
**130**	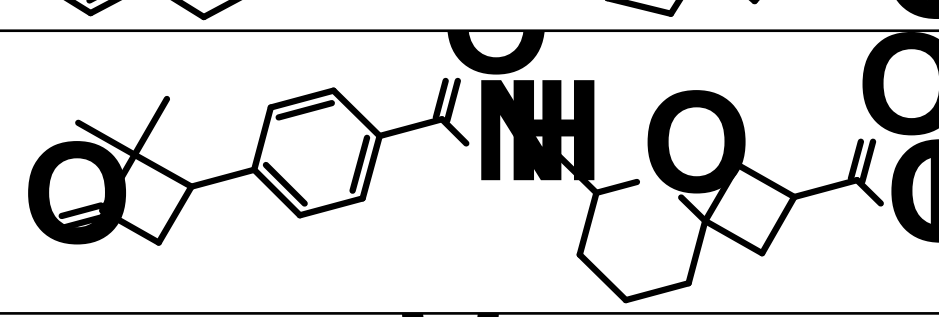	Z6242298398
**131**	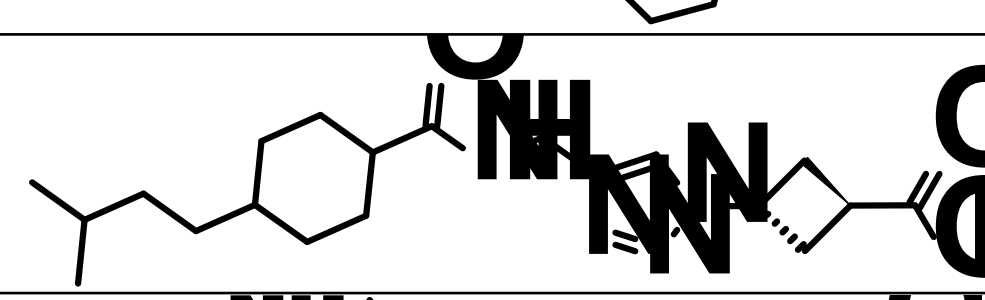	Z6681383318
**132**	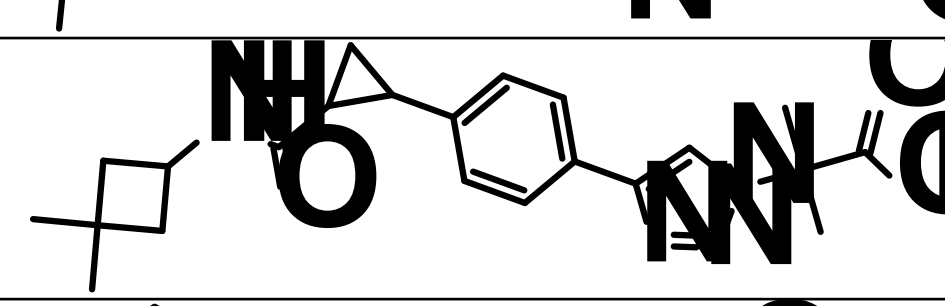	Z9698696253
**133**	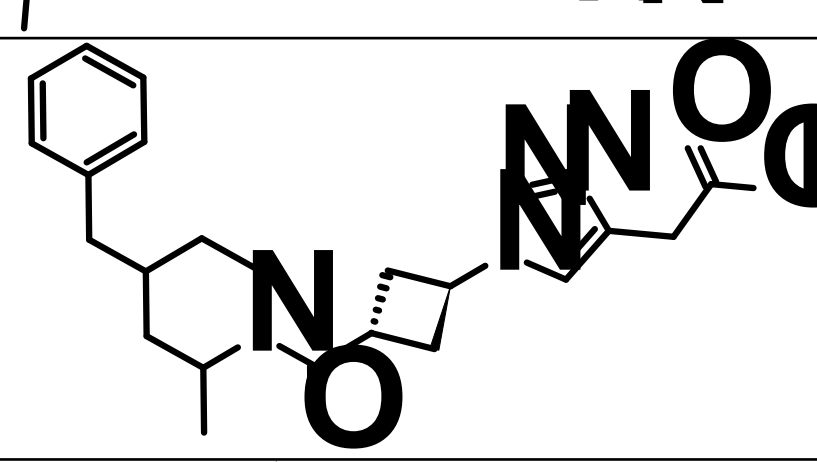	Z9698696254
**134**	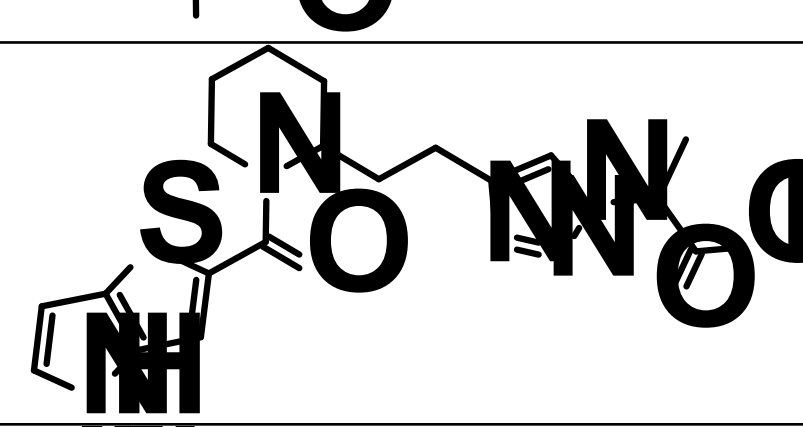	Z9698696259
**135**	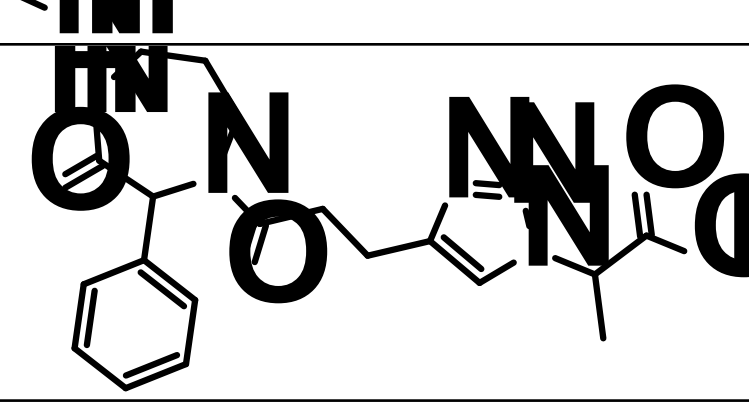	Z9698696260
**136**	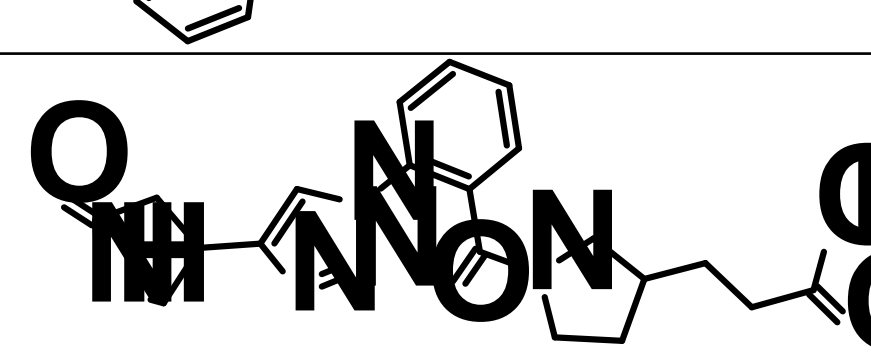	Z9698696261
**137**	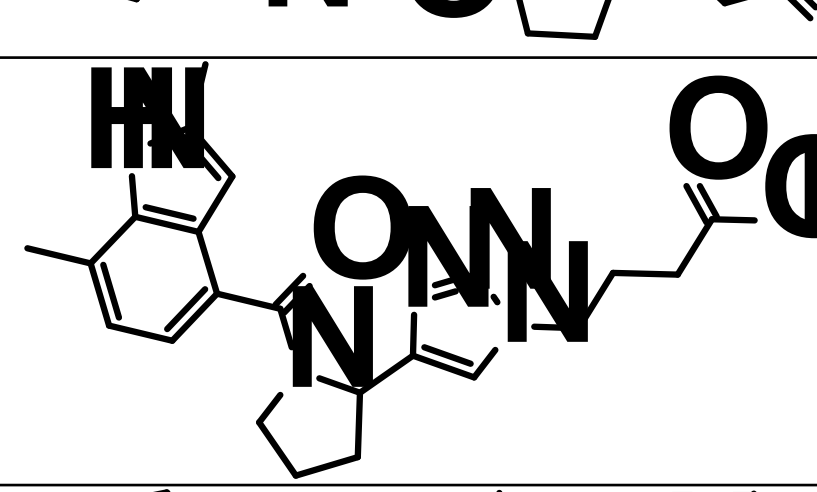	Z9698696262
**138**	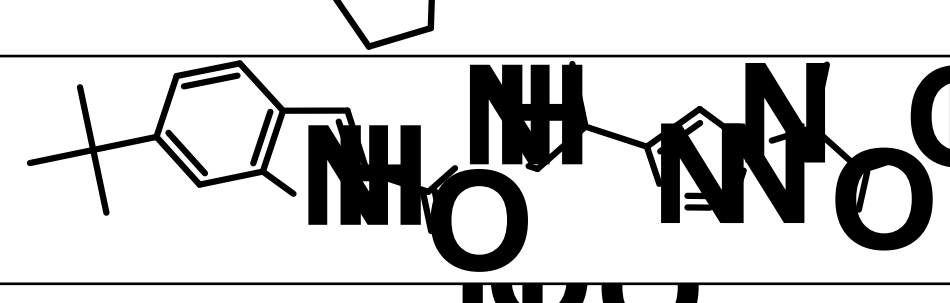	Z9698696321
**139**	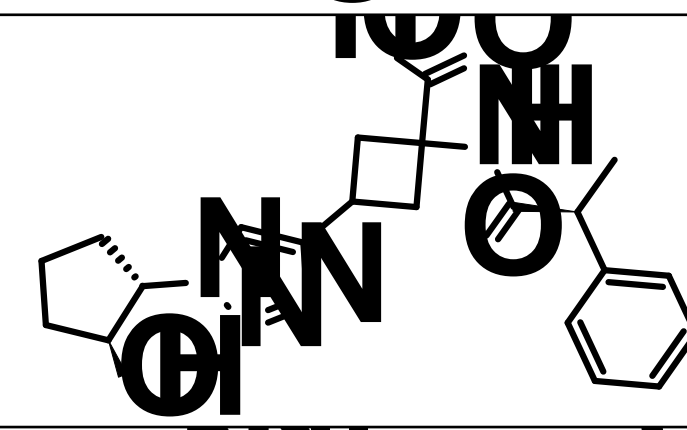	Z9698696322
**140**	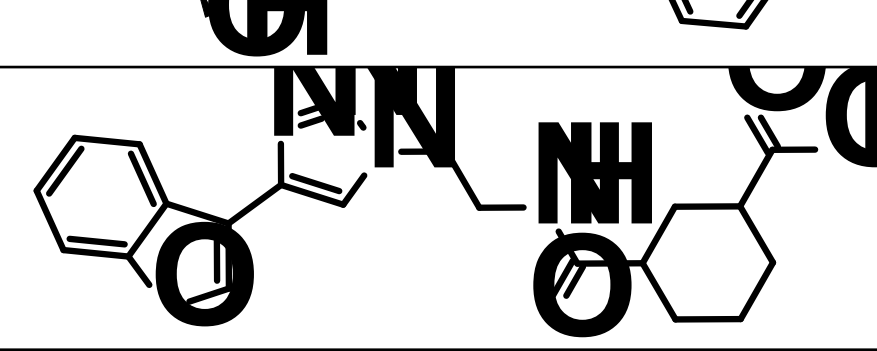	Z9698696332
**141**	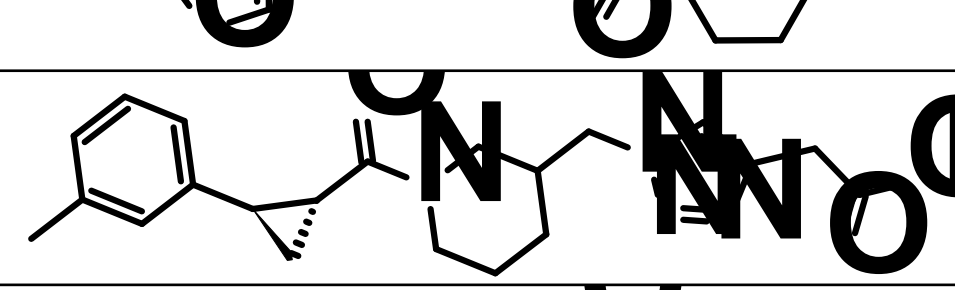	Z9698696334
**142**	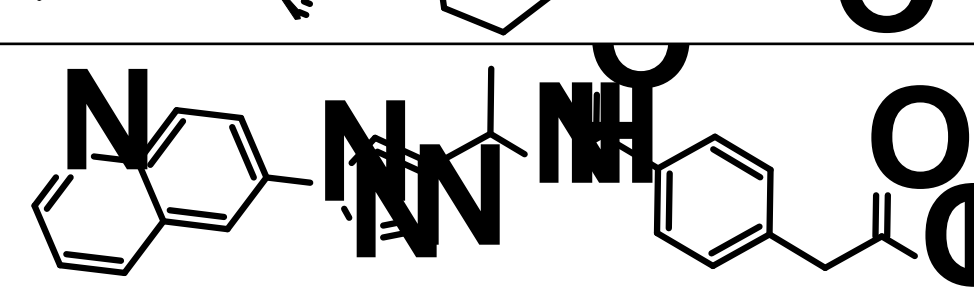	Z9698696336

**143**	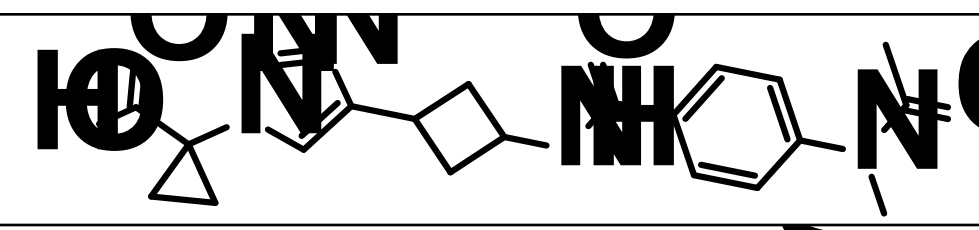	Z9698696372
**144**	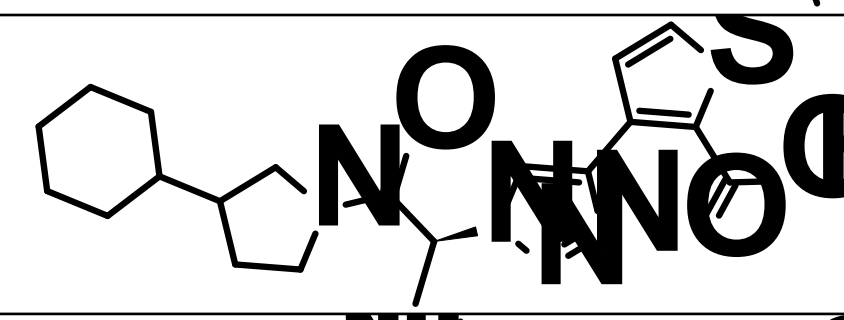	Z9698696339
**145**	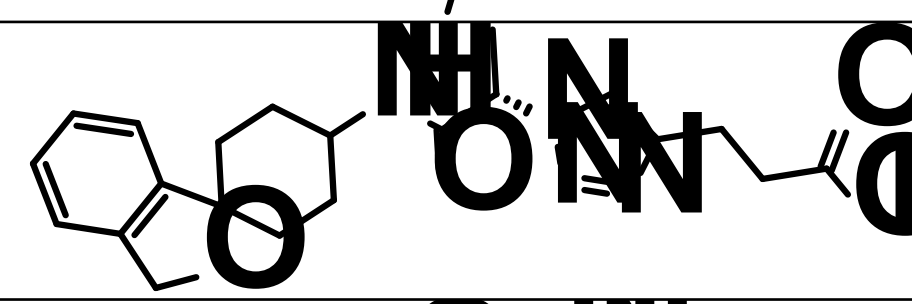	Z9698696340
**146**	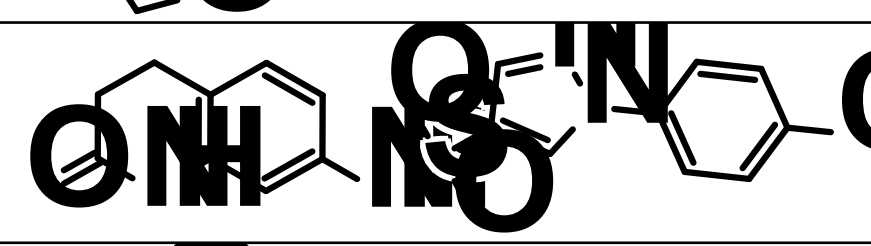	Z9698696295
**147**	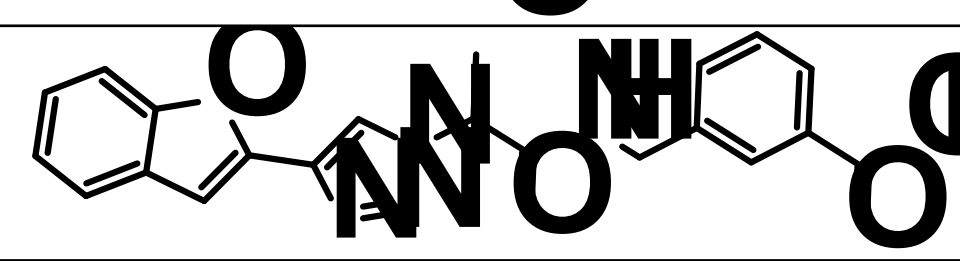	Z9698696343
**148**	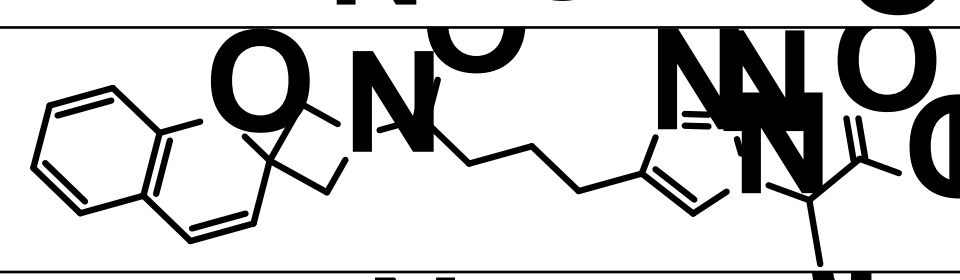	Z9698696399
**149**	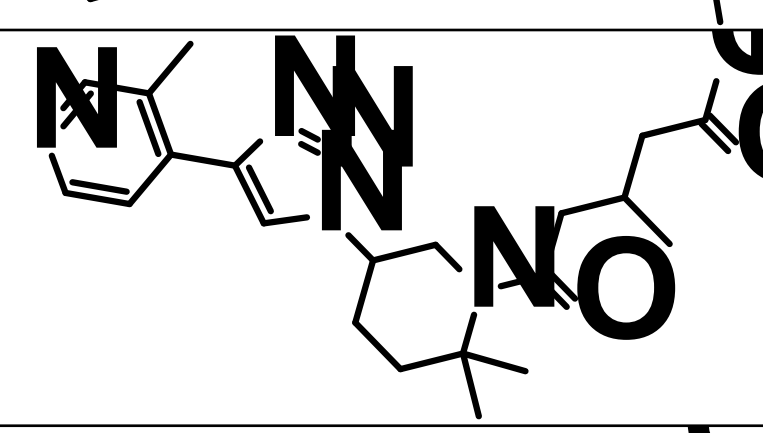	Z9698696400
**150**	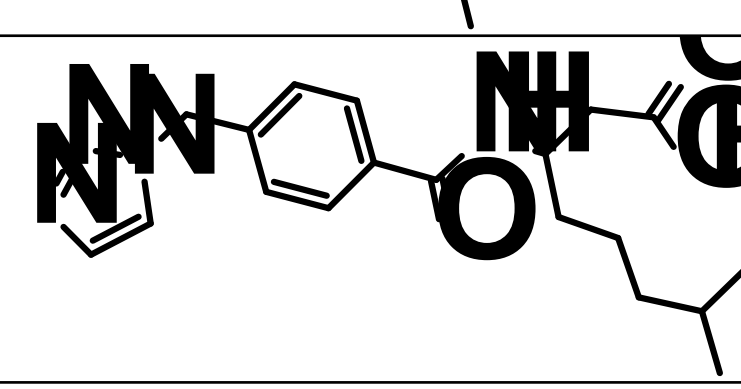	Z5292659966

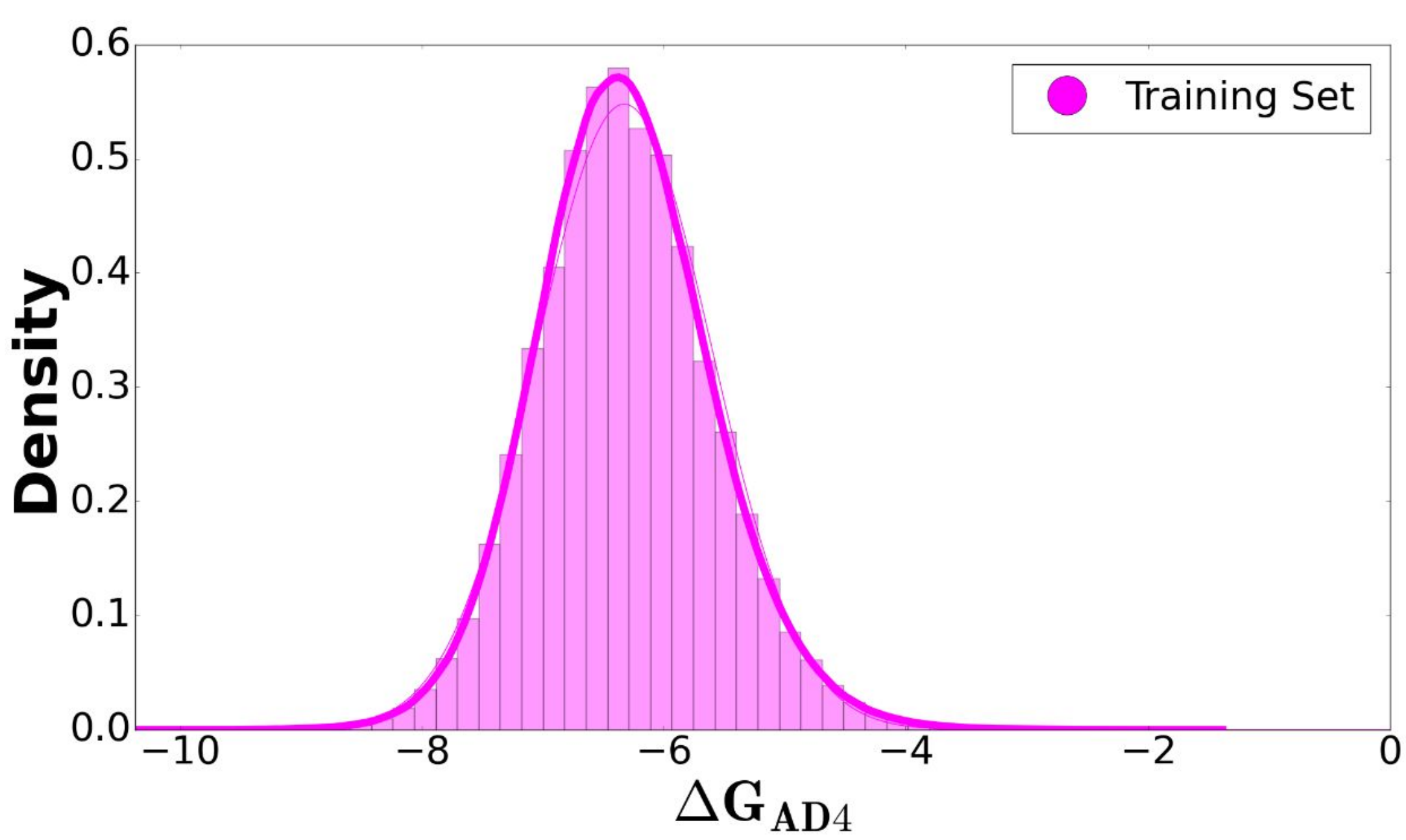

Figure S1. Distribution plot of the 2.4M molecules docked for training purposes.

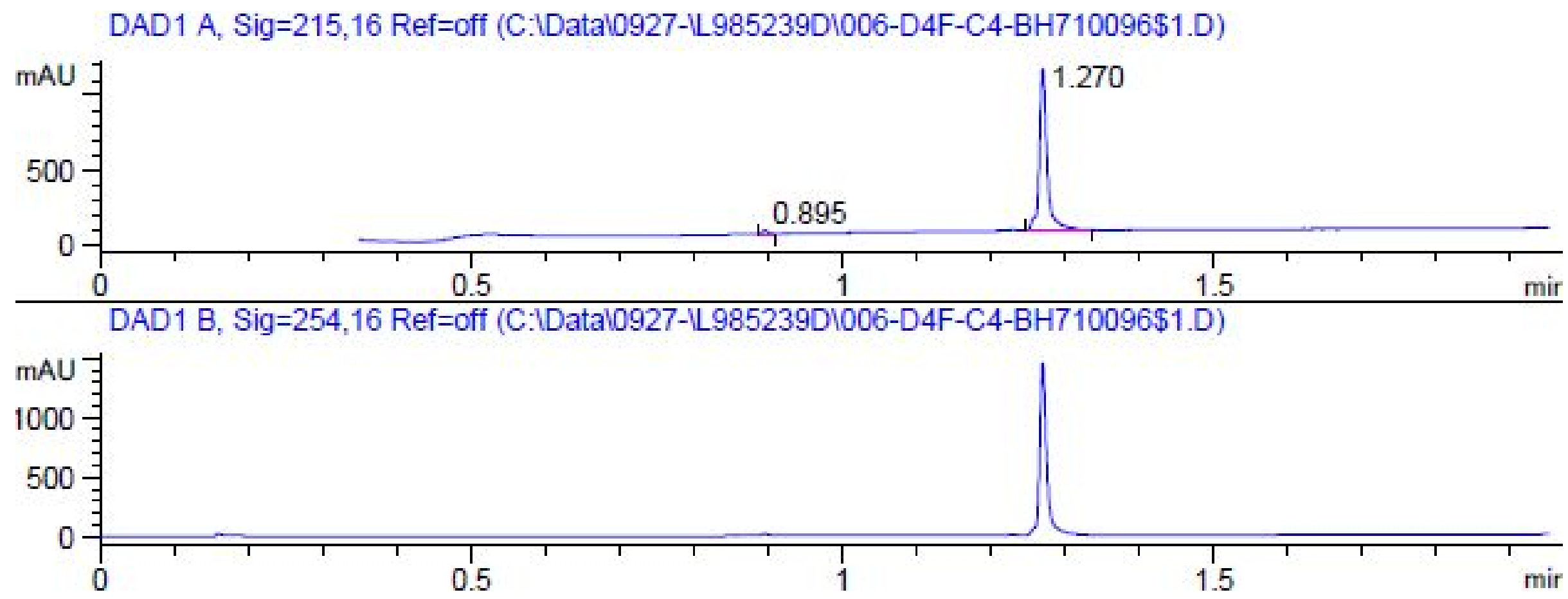

#	Time	Area%
1	0.895	1.44
2	1.270	98.56

Figure S2. HPLC Spectra for **1.**

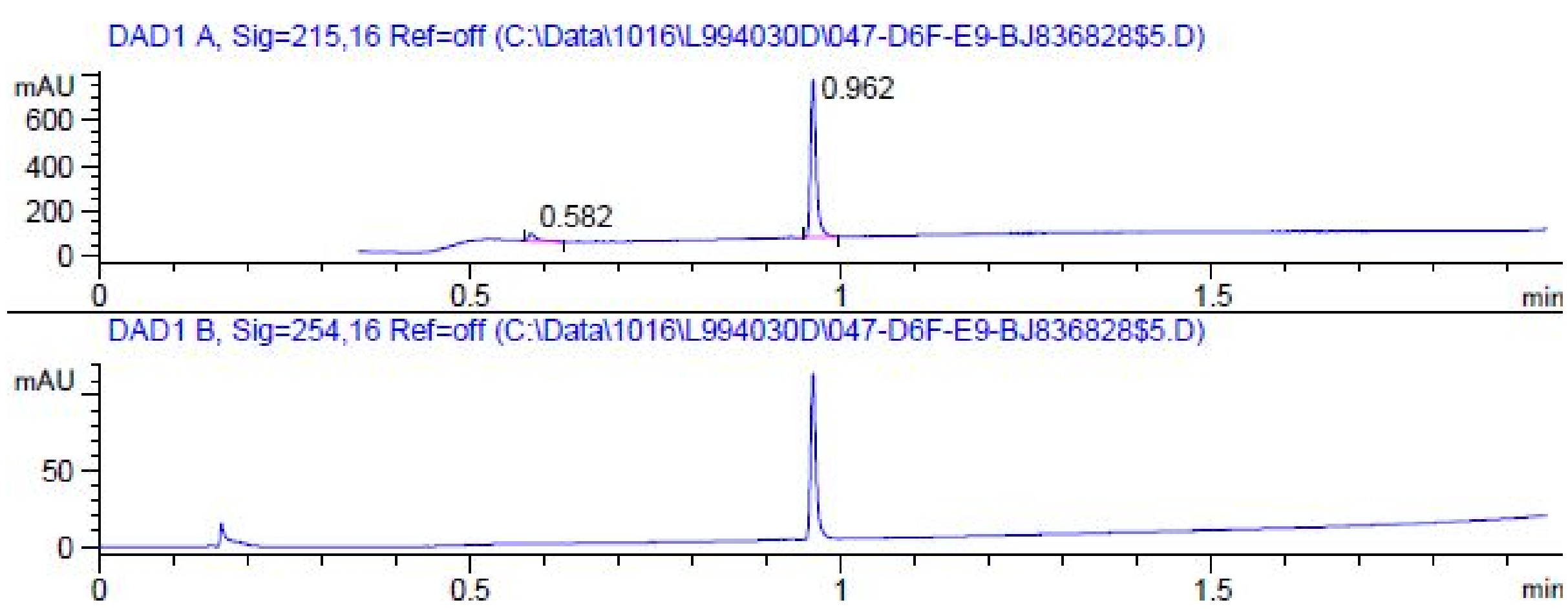

#	Time	Area%
1	0.582	5.87
2	0.962	94.13

Figure S3. HPLC Spectra for **5**

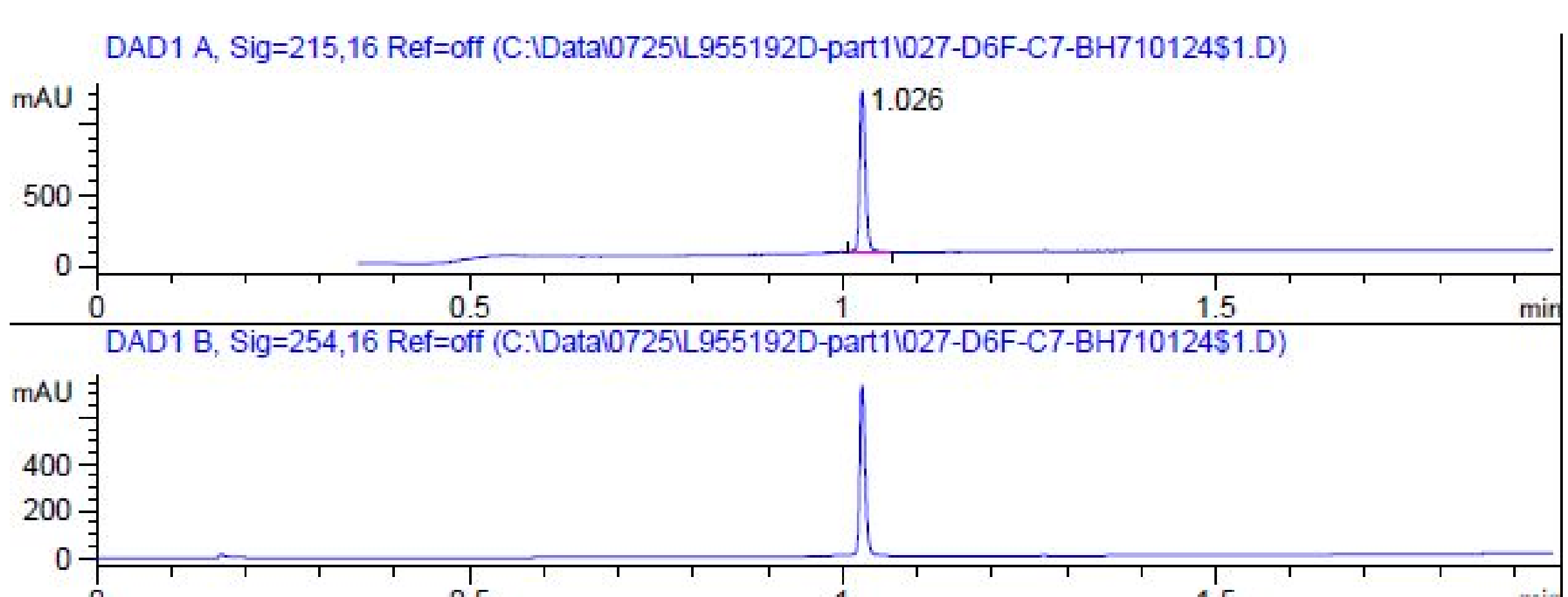

#	Time	Area%
1	1.026	100.00

Figure S4. HPLC Spectra for **10**

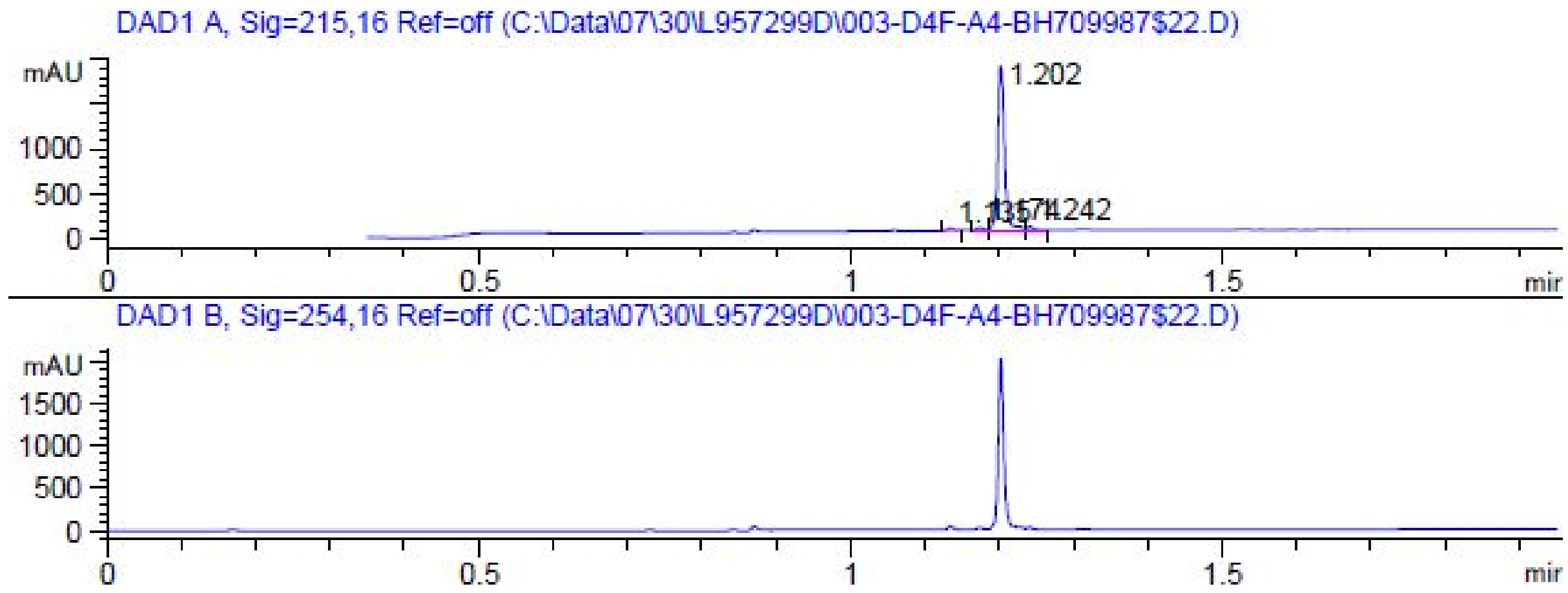

#	Time	Area%
1	1.135	1.24
2	1.174	1.30
3	1.202	95.54
4	1.242	1.91

Figure S5. HPLC Spectra for **24**

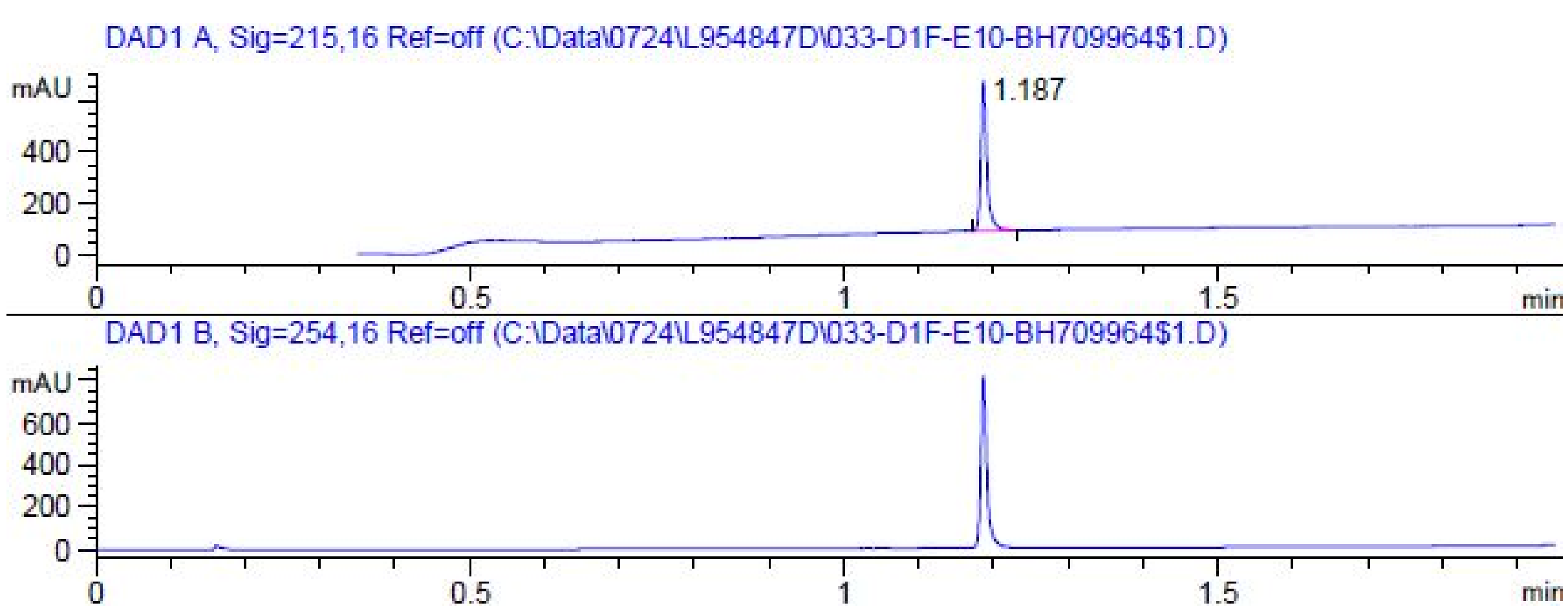

#	Time	Area%
1	1.187	100.00

Figure S6. HPLC Spectra for **28**

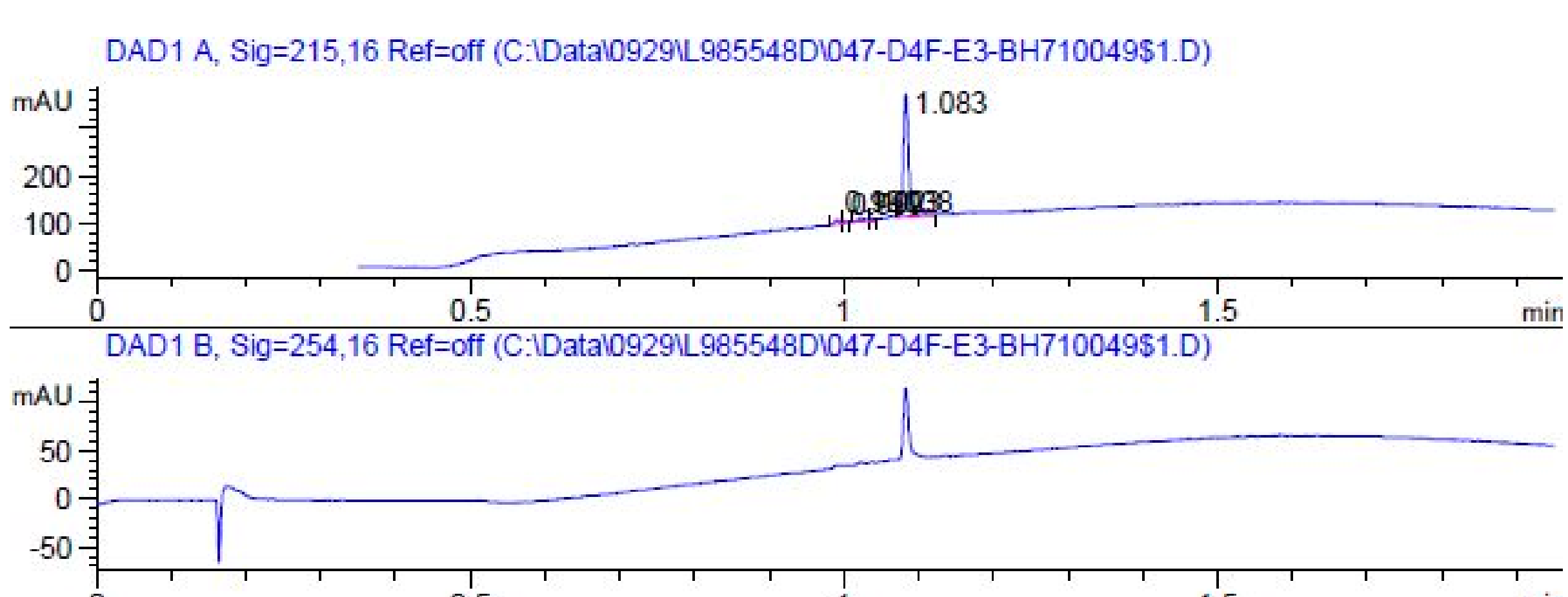

#	Time	Area%
1	0.991	3.25
2	0.999	2.03
3	1.023	2.76
4	1.038	1.62
5	1.083	90.34

Figure S7. HPLC Spectra for **50**

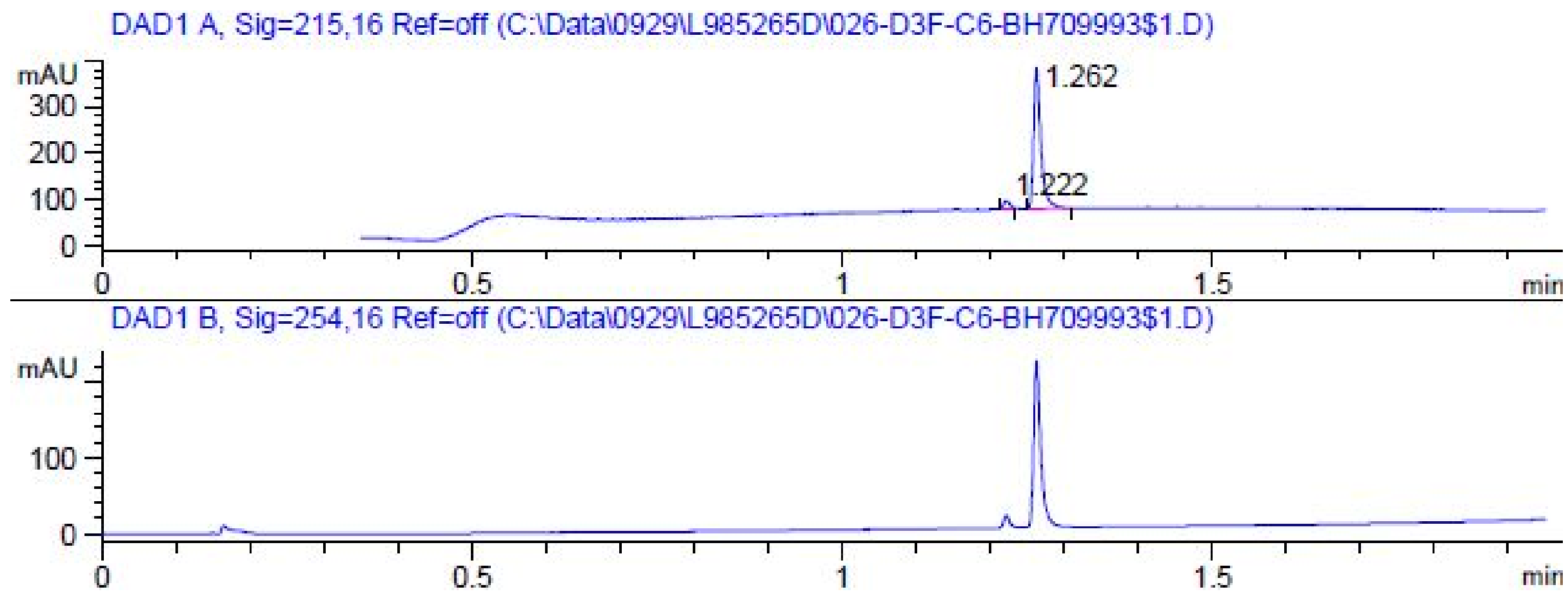

#	Time	Area%
1	1.222	4.80
2	1.262	95.20

Figure S8. PLC Spectra for **58**

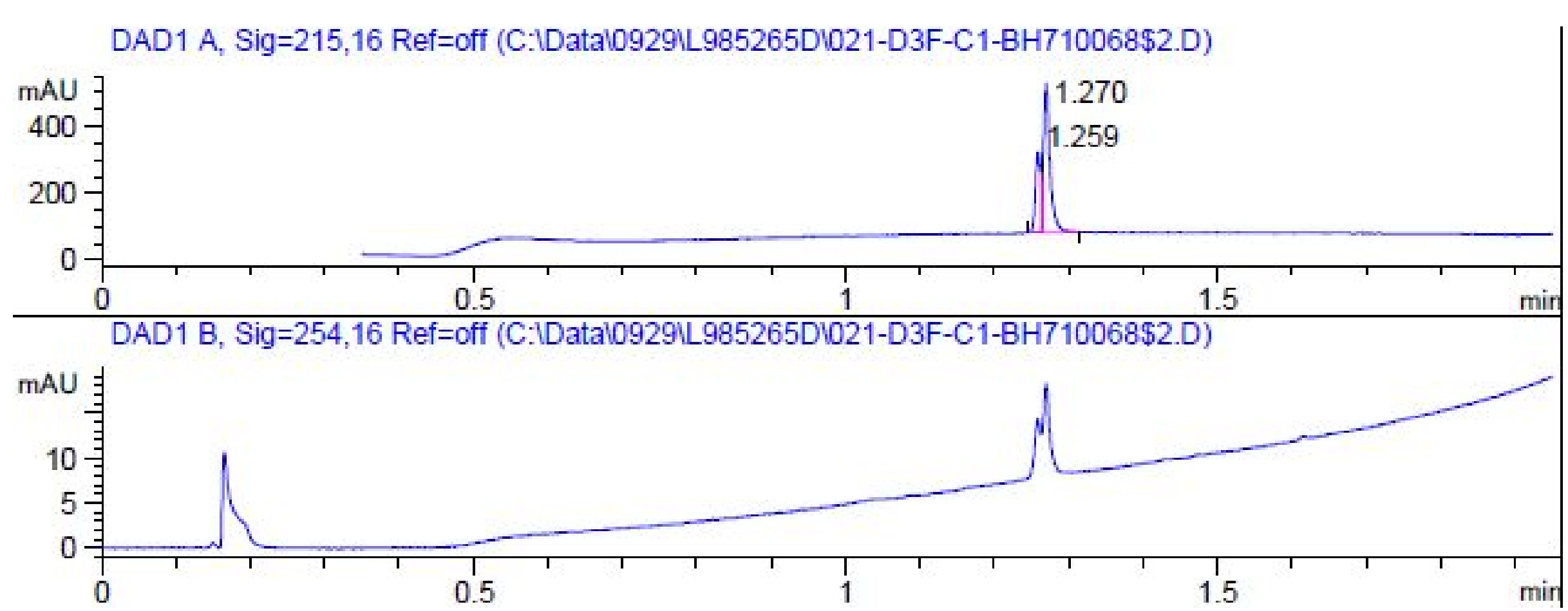

#	Time	Area%
1	1.259	27.96
2	1.270	72.04

Figure S9. HPLC Spectra for **63**

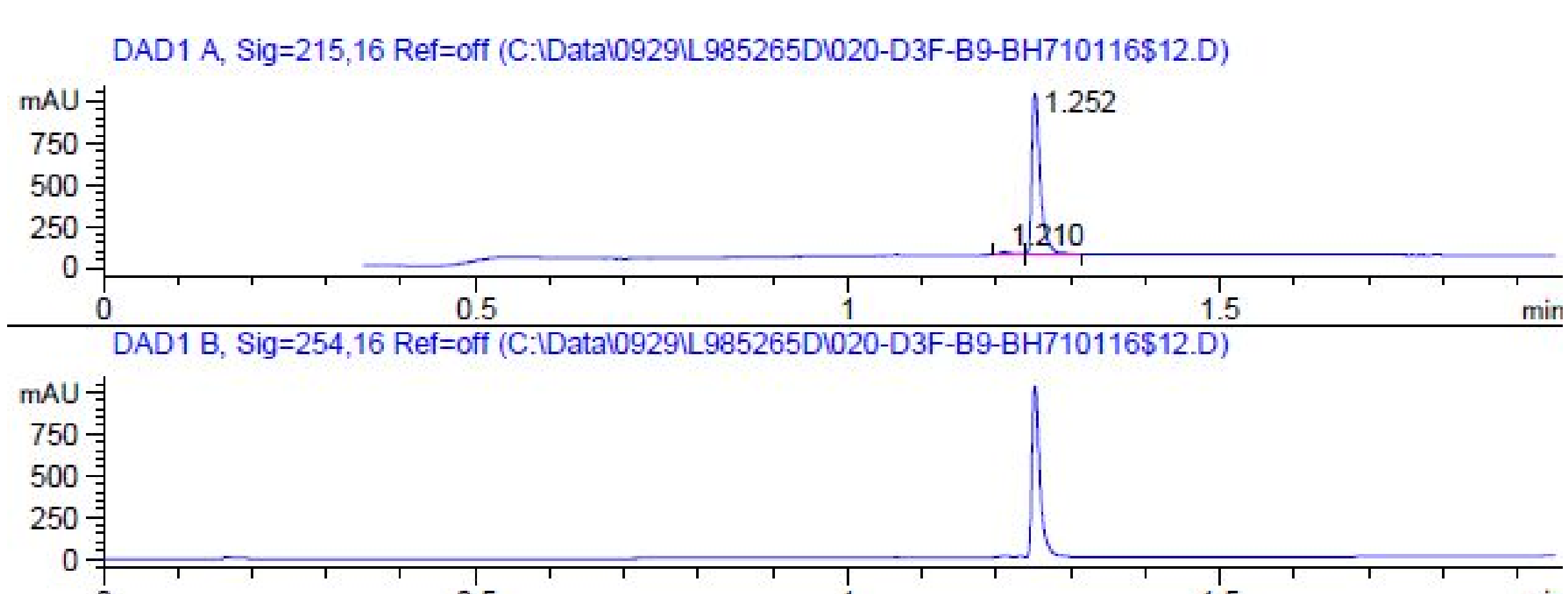

#	Time	Area%
1	1.210	2.40
2	1.252	97.60

Figure S10. HPLC Spectra for **64**

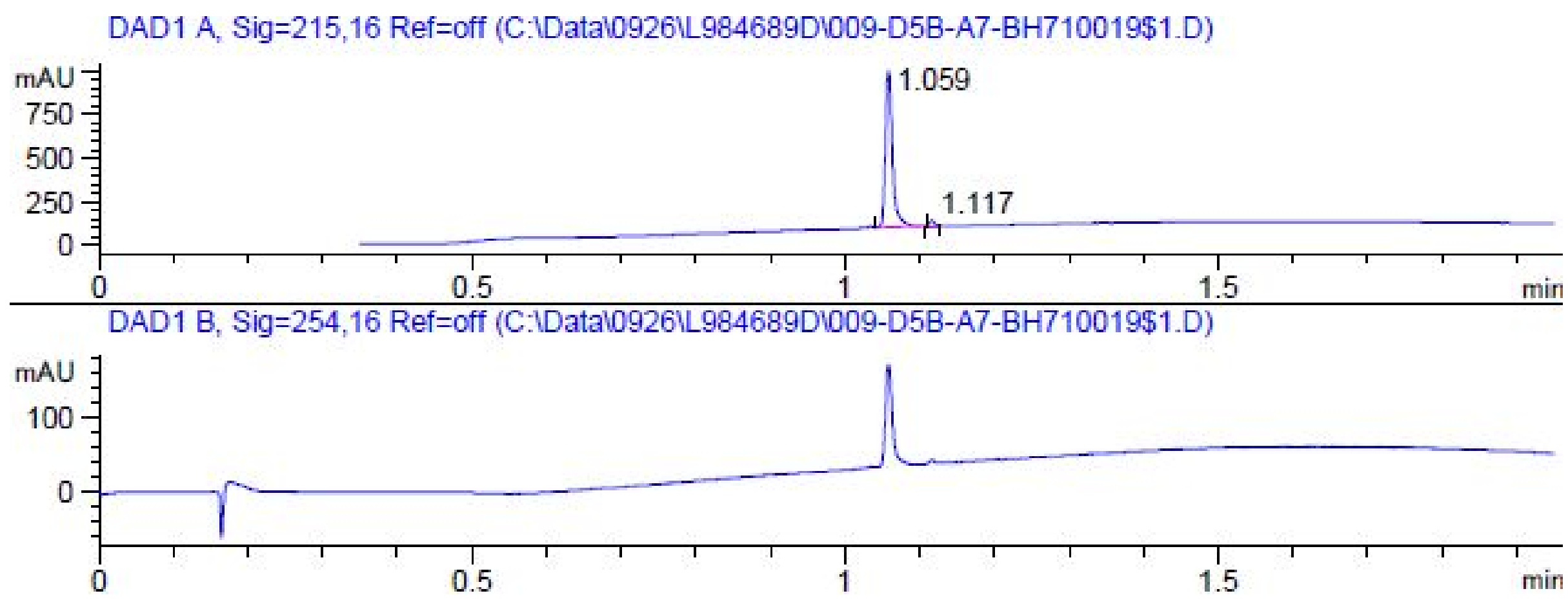

#	Time	Area%
1	1.059	97.56
2	1.117	2.44

Figure S11. HPLC Spectra for **68**

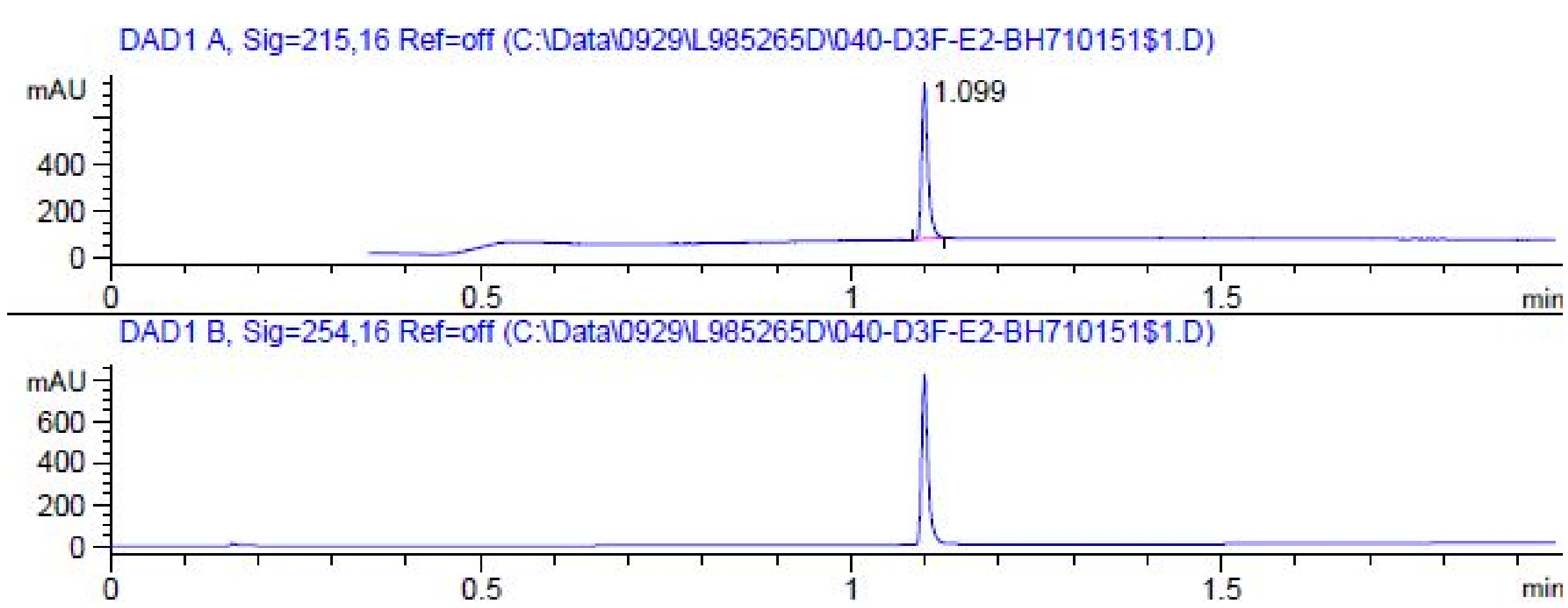

#	Time	Area%
1	1.099	100.00

Figure S12. HPLC Spectra for **100**

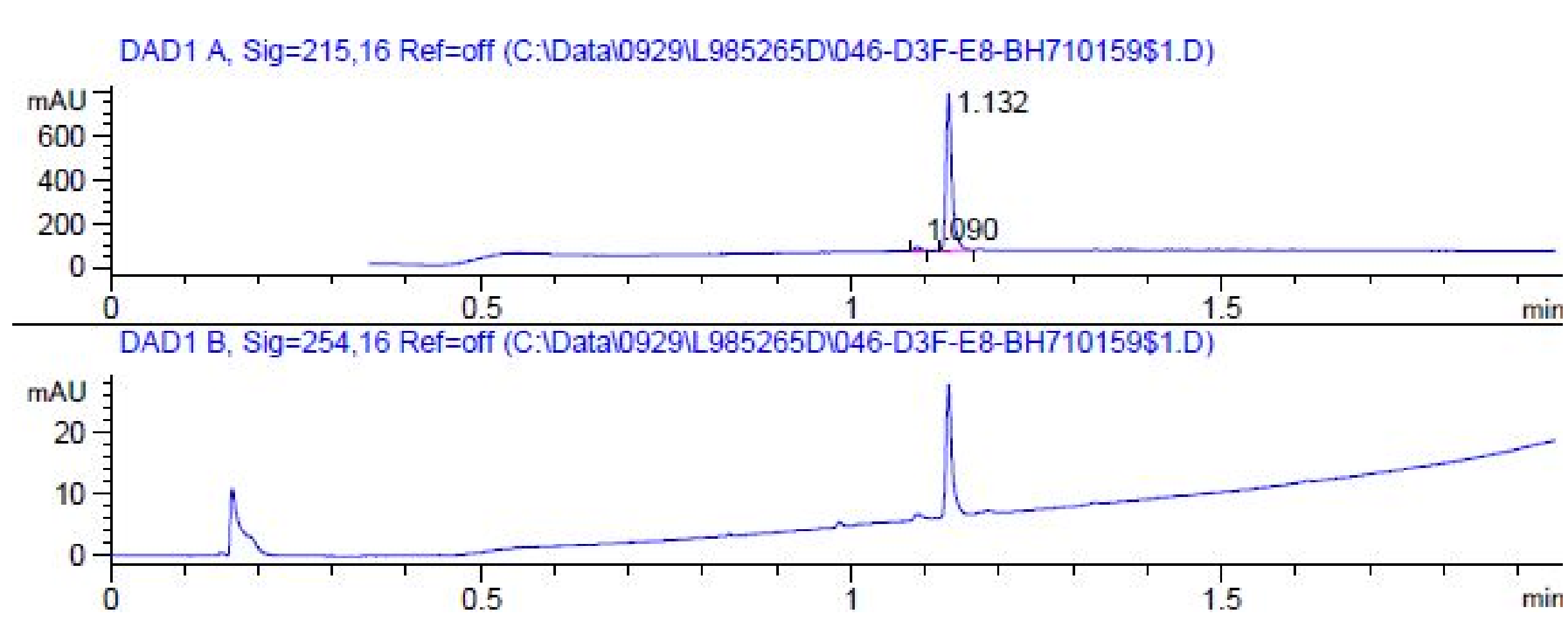

#	Time	Area%
1	1.090	2.31
2	1.132	97.69

Figure S13. HPLC Spectra for **104**

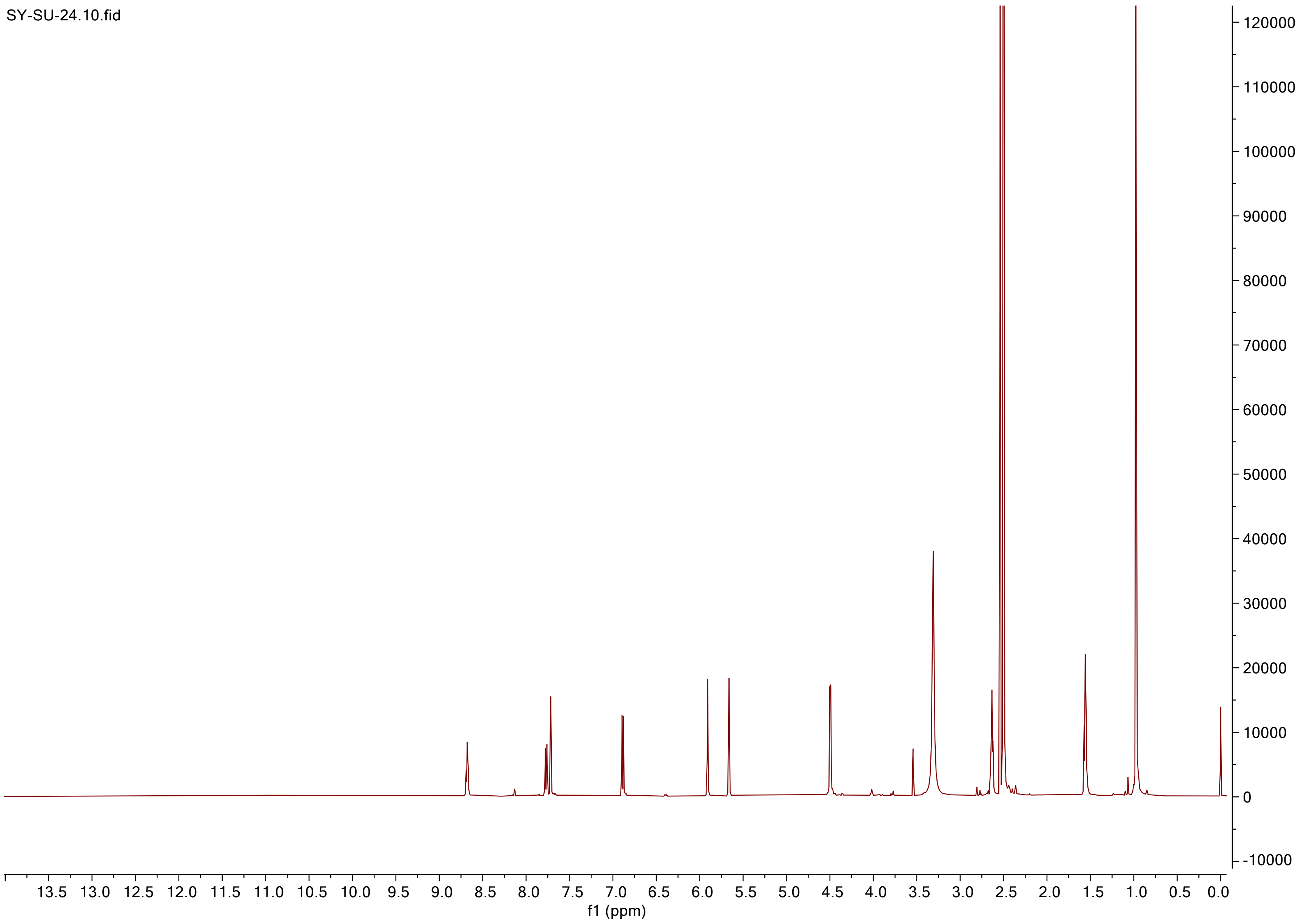

Figure S14. 1H-NMR Spectra for **24**

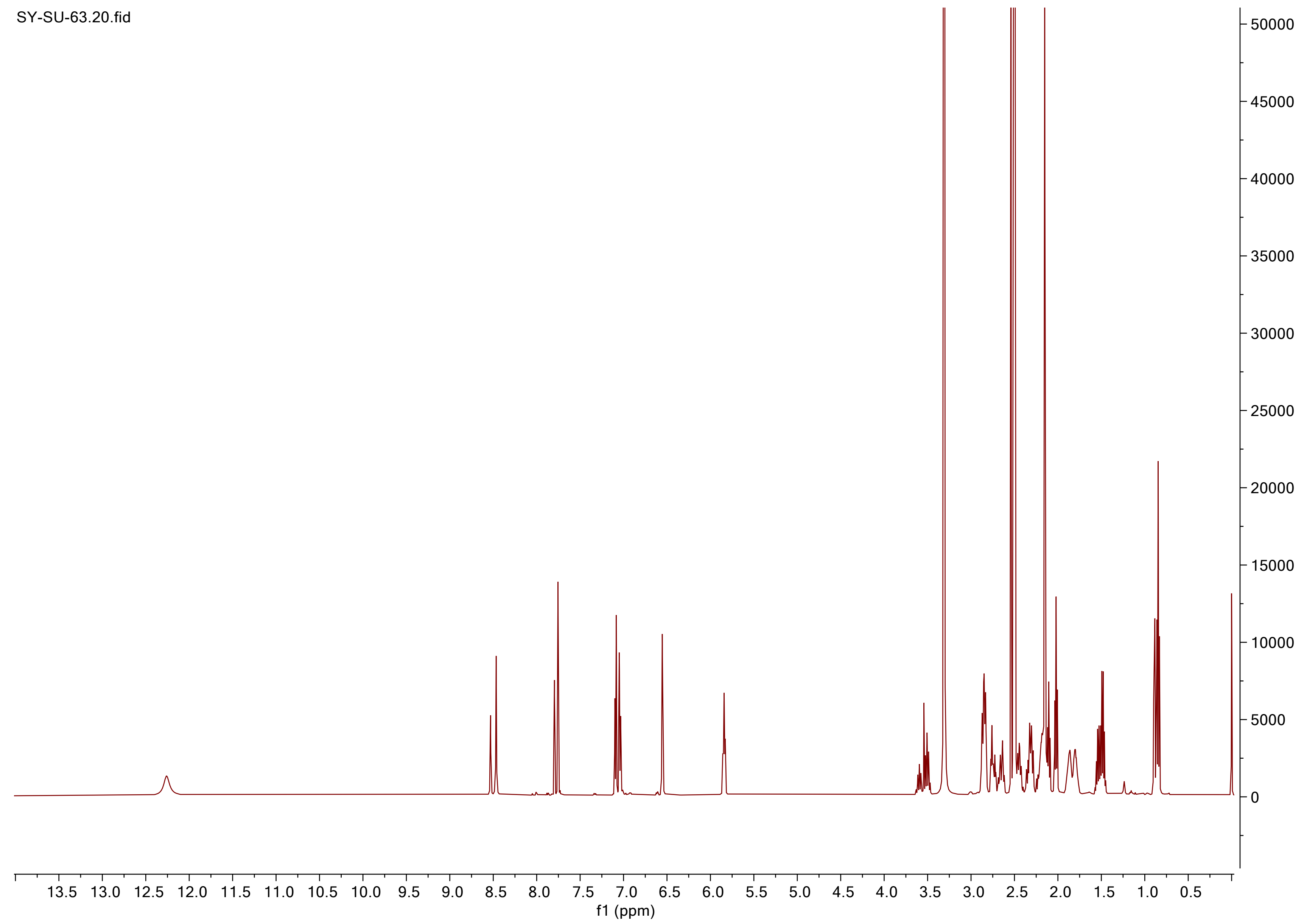

Figure S15. : 1H-NMR Spectra for **63**

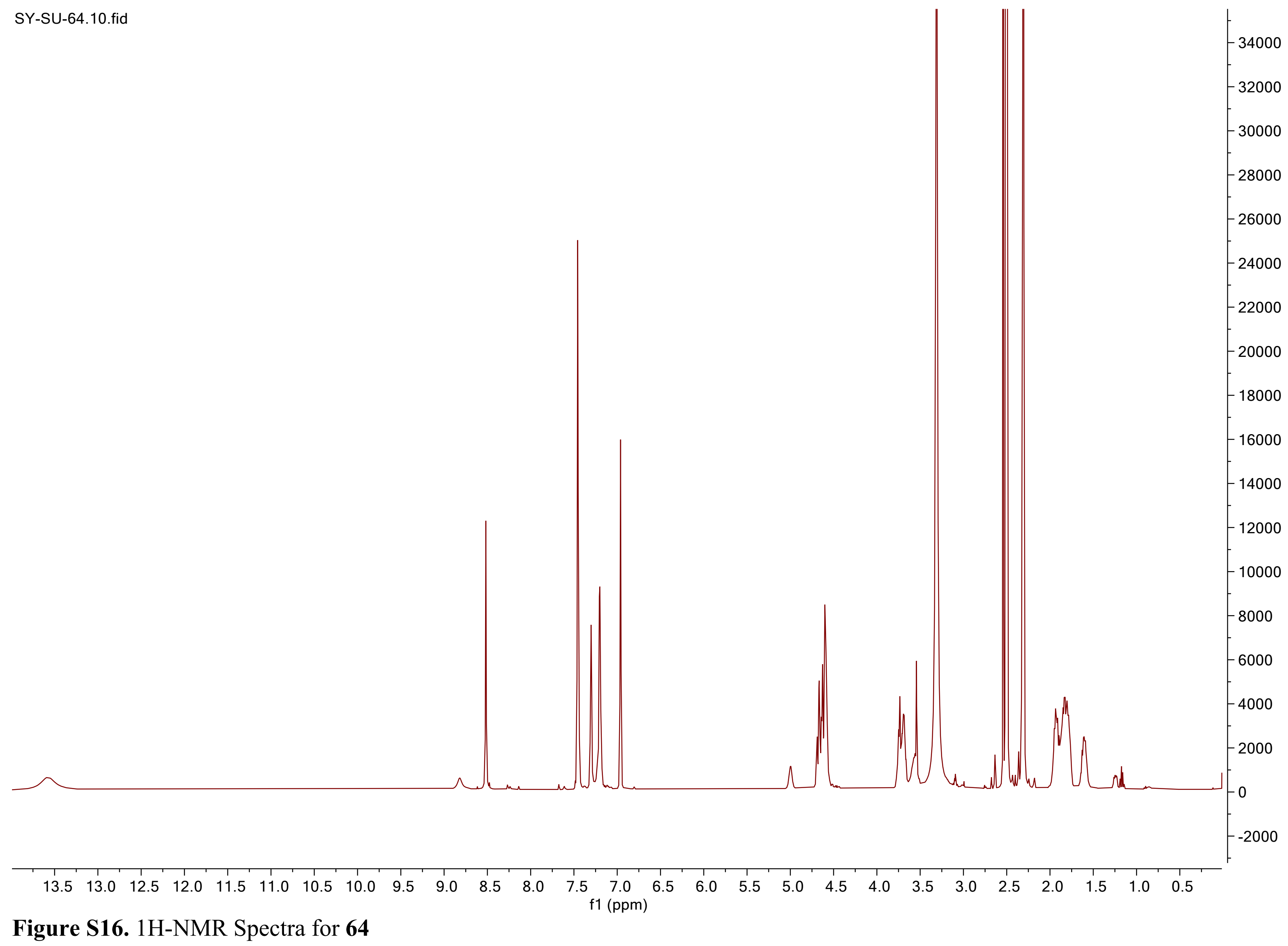

Figure S16. 1H-NMR Spectra for **64**

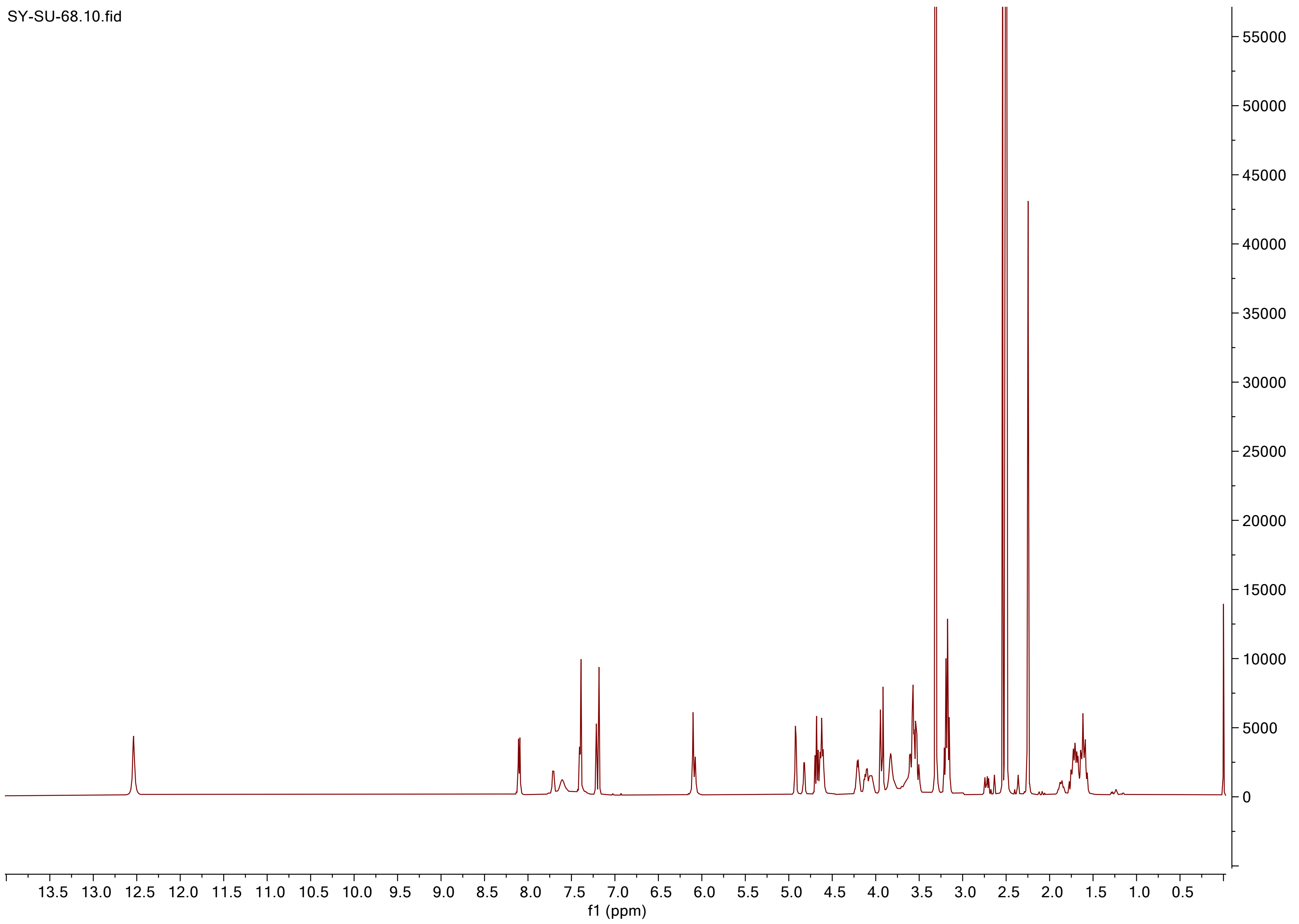

Figure S17. 1H-NMR Spectra for **68**

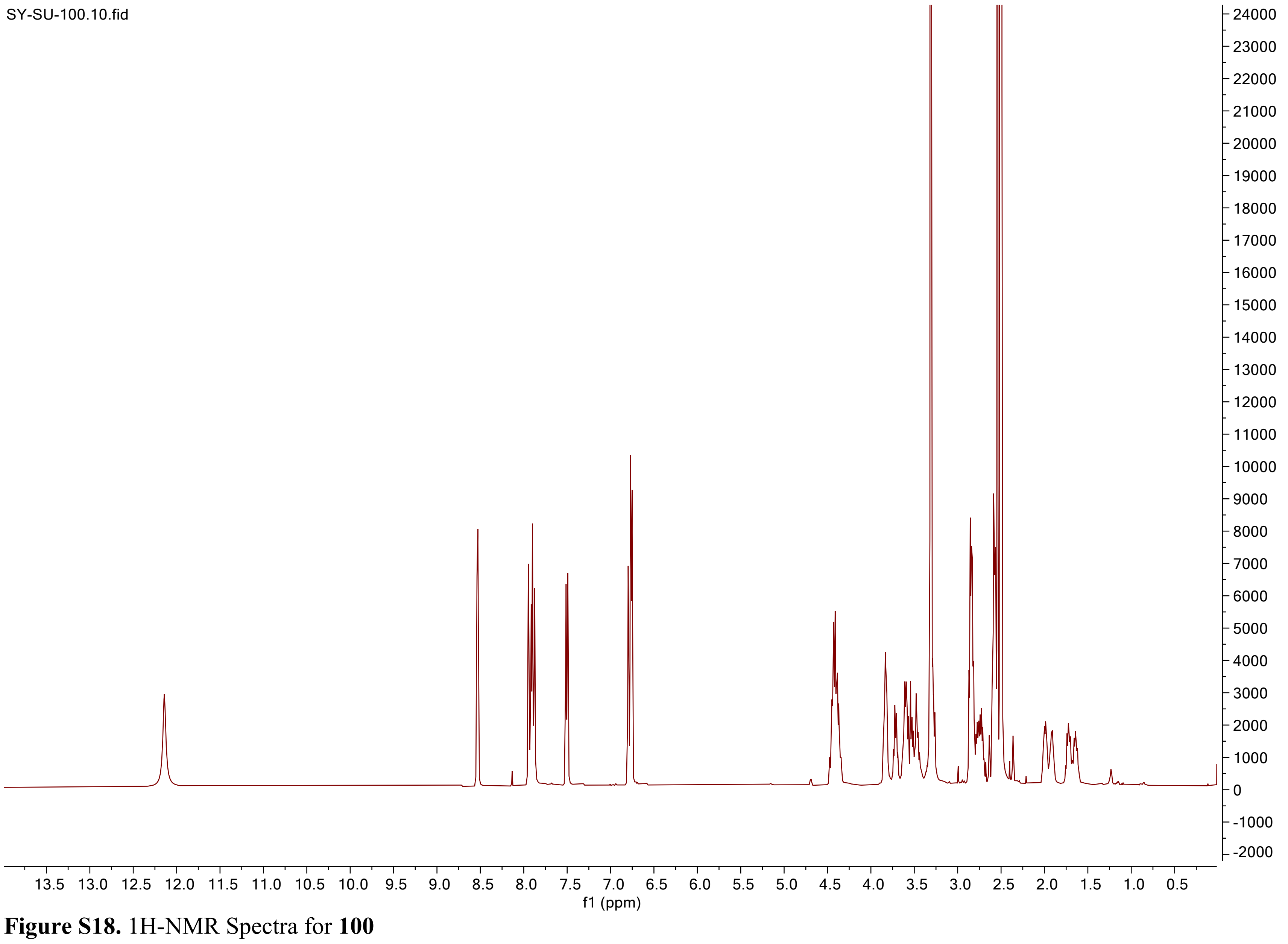

Figure S18. 1H-NMR Spectra for **100**

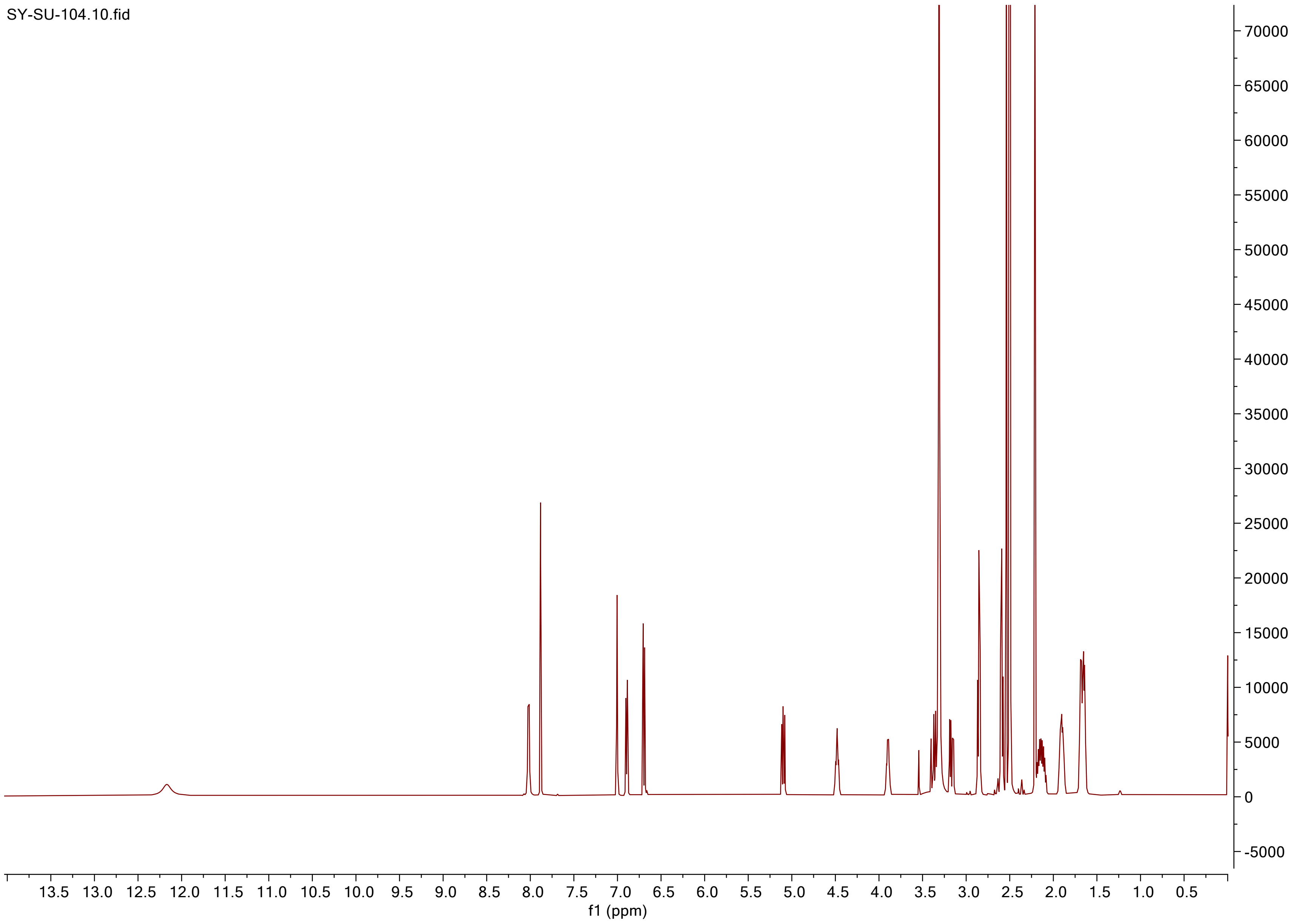

Figure S19. 1H-NMR Spectra for **104**

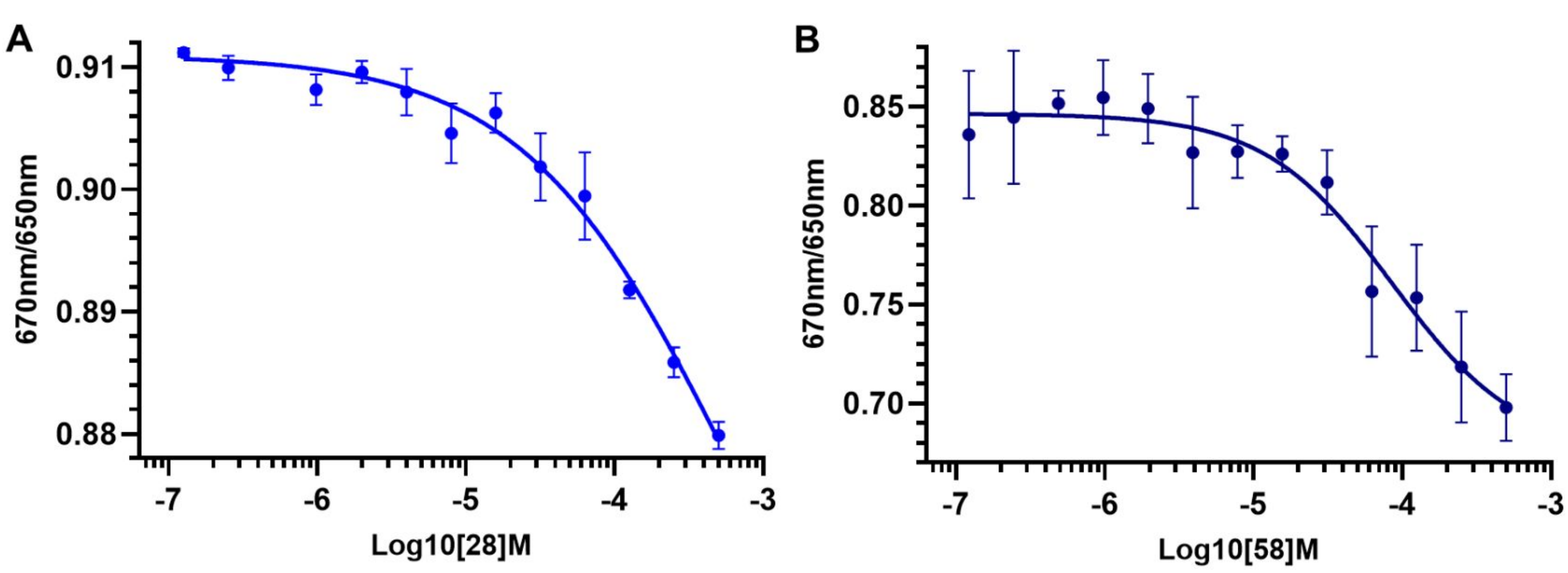

Figure S20. Dose–response curves obtained using Monolith X (F670/F650 spectral shift mode). His-tagged CD28 was incubated with serial dilutions of compounds, and normalized fluorescence ratios were plotted against concentration. Data represent mean ± SEM. Solid lines indicate nonlinear regression fits used to calculate dissociation constants.(A) AU28, Kd = 426.93 µM and (B) AU58, Kd = 85.14 µM.

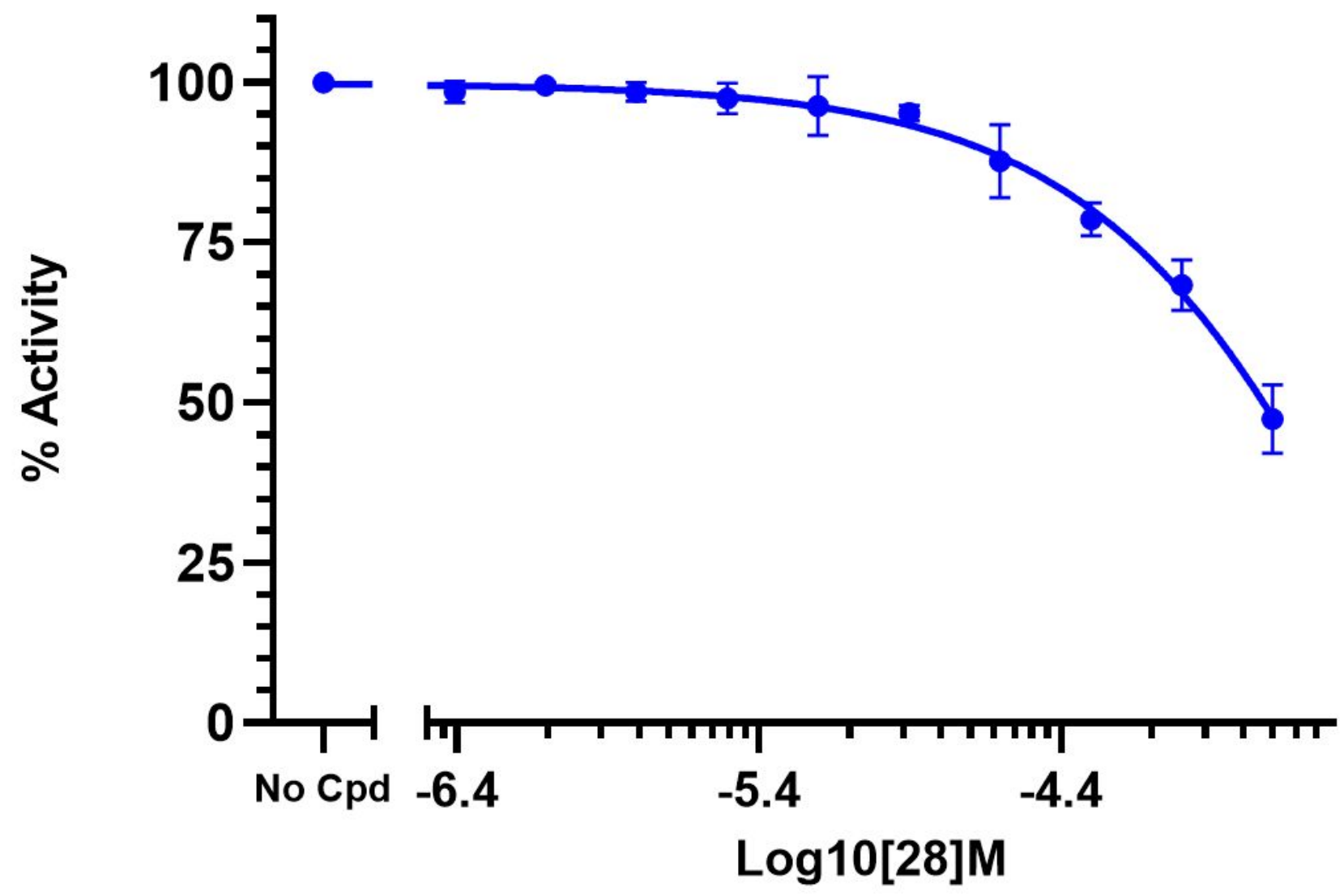

Figure S21. Dose–response inhibition curve of AU28 in a competitive CD28–CD80 ELISA, with an IC_{50} of 411.3 μM determined by nonlinear regression.

MST-Based Selectivity Assessment of Compound 100

To evaluate selectivity, MST-based binding assays were performed for compound 100 against related immune targets, including ICOS, CD80, and CD86. Recombinant human extracellular domains (ECDs) of ICOS, CD80, and CD86 (Acro Biosystems) were used. Each protein was labeled with RED-tris-NTA 2nd Generation dye (NanoTemper Technologies) according to the manufacturer's instructions in assay buffer (PBS, pH 7.4, supplemented with 0.05% Tween-20) and incubated for 30 min at room temperature in the dark.

Compound 100 was prepared as a 1:1 serial dilution series spanning concentrations from 500 μM to 1 nM in assay buffer containing 2% DMSO. Labeled protein was mixed 1:1 with compound solutions to yield a final protein concentration of 100 nM and incubated for 15 min at room temperature prior to measurement.

Samples were loaded into Monolith premium capillaries and measured at 25 °C using a Monolith X instrument (NanoTemper Technologies) in spectral shift detection mode. Binding was assessed by monitoring changes in the normalized fluorescence emission ratio (F670/F650) upon IR-laser excitation, and data were analyzed using MO.Affinity Analysis software.

Under the conditions tested, no concentration-dependent thermophoretic response or detectable binding was observed for ICOS, CD80, or CD86.

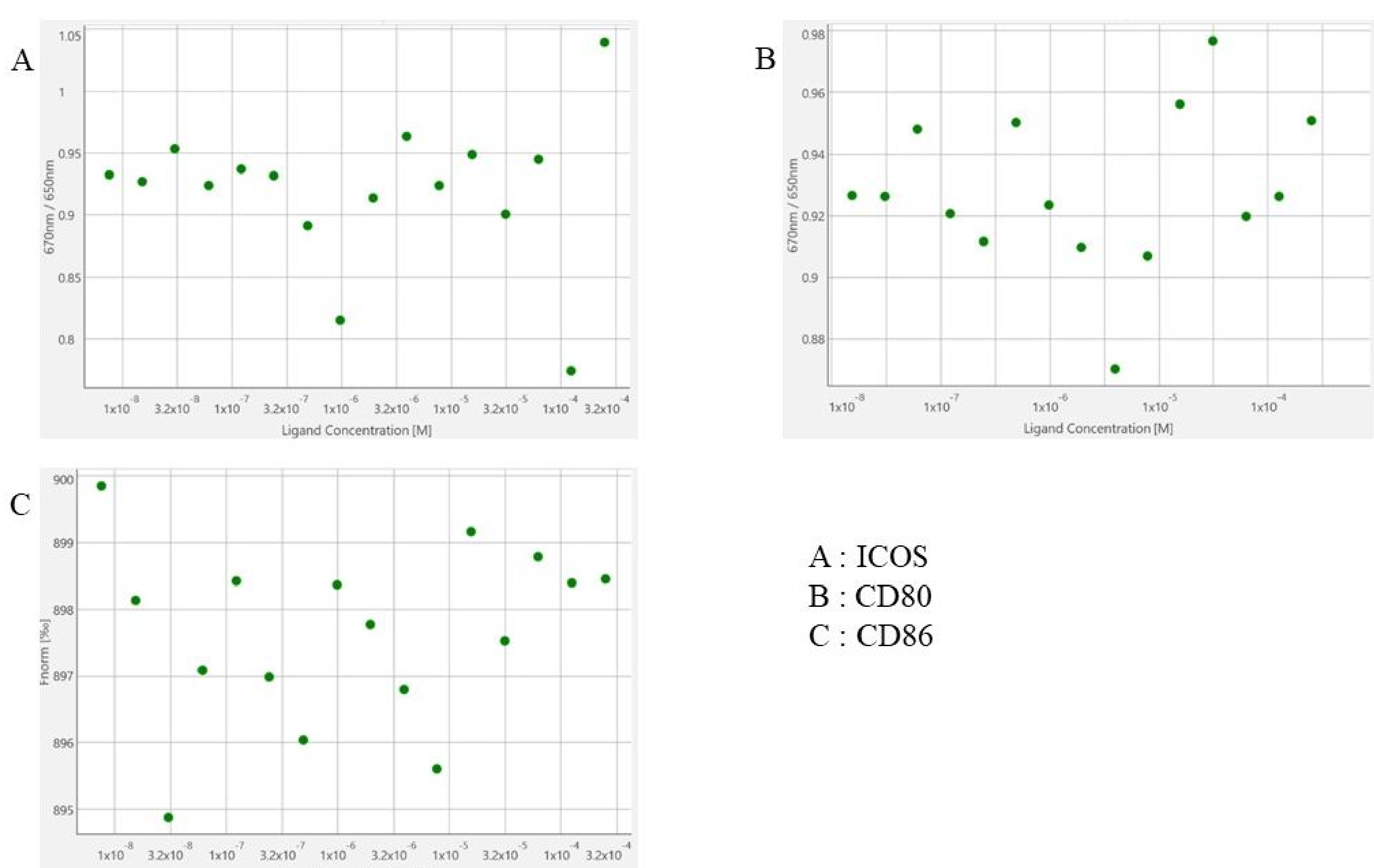

Figure S22. MST-based selectivity assessment of compound 100 against related immune targets. MST measurements using recombinant human ICOS (A), CD80 (B), and CD86 (C) ECDs showed no concentration-dependent signal change, indicating no detectable binding under the conditions tested.
